# What are the risk factors for death among children with pneumonia in low- and middle-income countries? A systematic review

**DOI:** 10.7189/jogh.13.05003

**Published:** 2023-02-24

**Authors:** Chris Wilkes, Mohamed Bava, Hamish R Graham, Trevor Duke, ARI Review group, Trevor Duke, Hamish Graham, Steve Graham, Amy Gray, Amanda Gwee, Claire von Mollendorf, Kim Mulholland, Fiona Russell, Maeve Hume-Nixon, Saniya Kazi, Priya Kevat, Eleanor Neal, Cattram Nguyen, Alicia Quach, Rita Reyburn, Kathleen Ryan, Patrick Walker, Chris Wilkes, Poh Chua, Yasir Nisar, Jonathon Simon, Wilson Were

**Affiliations:** 1 Murdoch Children’s Research Institution, Royal Children’s Hospital, Parkville, Victoria, Australia; 2 Department of Paediatrics, University of Melbourne, Royal Children’s Hospital, Parkville, Victoria, Australia

## Abstract

**Background:**

Knowledge of the risk factors for and causes of treatment failure and mortality in childhood pneumonia is important for prevention, diagnosis, and treatment at an individual and population level. This review aimed to identify the most important risk factors for mortality among children aged under ten years with pneumonia.

**Methods:**

We systematically searched MEDLINE, EMBASE, and PubMed for observational and interventional studies reporting risk factors for mortality in children (aged two months to nine years) in low- and middle-income countries (LMICs). We screened articles according to specified inclusion and exclusion criteria, assessed risk of bias using the EPHPP framework, and extracted data on demographic, clinical, and laboratory risk factors for death. We synthesized data descriptively and using Forest plots and did not attempt meta-analysis due to the heterogeneity in study design, definitions, and populations.

**Findings:**

We included 143 studies in this review. Hypoxaemia (low blood oxygen level), decreased conscious state, severe acute malnutrition, and the presence of an underlying chronic condition were the risk factors most strongly and consistently associated with increased mortality in children with pneumonia. Additional important clinical factors that were associated with mortality in the majority of studies included particular clinical signs (cyanosis, pallor, tachypnoea, chest indrawing, convulsions, diarrhoea), chronic comorbidities (anaemia, HIV infection, congenital heart disease, heart failure), as well as other non-severe forms of malnutrition. Important demographic factors associated with mortality in the majority of studies included age <12 months and inadequate immunisation. Important laboratory and investigation findings associated with mortality in the majority of studies included: confirmed Pneumocystis jirovecii pneumonia (PJP), consolidation on chest x-ray, pleural effusion on chest x-ray, and leukopenia. Several other demographic, clinical and laboratory findings were associated with mortality less consistently or in a small numbers of studies.

**Conclusions:**

Risk assessment for children with pneumonia should include routine evaluation for hypoxaemia (pulse oximetry), decreased conscious state (e.g. AVPU), malnutrition (severe, moderate, and stunting), and the presence of an underlying chronic condition as these are strongly and consistently associated with increased mortality. Other potentially useful risk factors include the presence of pallor or anaemia, chest indrawing, young age (<12 months), inadequate immunisation, and leukopenia.

Knowledge of the risk factors for and causes of treatment failure and mortality in childhood pneumonia is important for prevention, diagnosis, and treatment at an individual and population level [1]. From a preventive perspective it enables health care workers (HCWs) and public health workers to identify individuals and populations with modifiable risk factors for poor outcomes (e.g. malnutrition) and intervene. From a diagnosis and treatment perspective, risk-stratification is critical to ensure the right patients are prioritised for the right care (e.g. who needs admission vs home treatment) and encourages the most efficient use of available resources (e.g. money, staff time, medication).

One example of risk stratification is the World Health Organization (WHO) guidelines for childhood pneumonia which use known clinical risk factors to stratify patients into “severe” and “non-severe” classifications and provide practical guidance on referral and treatment [2-4]. In the WHO guidelines, stratification is based on presence or absence of central cyanosis, hypoxaemia (defined as oxygen saturations less than 90%), severe respiratory distress, and general danger signs including inability to drink or breastfeed, lethargy or unconsciousness, or convulsions [2].

The potential value of understanding risk factors goes beyond their utility in standardised clinical guidelines, offering opportunity to better understand population vulnerabilities and inform responses both at an individual patient and population level. The unacceptably high mortality rates among children with pneumonia (including those without signs of WHO severe pneumonia) makes understanding these risk factors even more important – particularly for children in low- and middle-income countries (LMICs) where the greatest burden lies [5]. Recent developments in clinical medicine and public health now offer a greater range of possible demographic, clinical, aetiological, and laboratory risk factors to consider [5]. While the advent of widespread vaccination against Streptococcus pneumoniae and Haemophilus influenzae type B (Hib), previously two of the most common pathogenic causes of pneumonia, has changed the aetiology of pneumonia and potentially further altered the causes of treatment failure and mortality [6].

This review aimed to identify clinical, demographic, and investigation findings that are associated with treatment failure or mortality in children (aged two months to nine years) with pneumonia in LMICs.

## METHODS

We conducted a systematic search of medical databases MEDLINE, EMBASE, and PubMed (for recent studies not yet indexed on MEDLINE) for studies reporting on factors associated with child pneumonia mortality (August 30, 2020). We mapped search terms to medical subject headings where possible, using Boolean operators to combine searches into our final systematic search query. We used synonyms of “pneumonia”, “mortality”, and “child” to target our search strategy, with oversight from an experienced Health Service Librarian to ensure all relevant papers were identified. We also searched reference lists of all included references for eligible studies. Additional methodological detail, including the Medline search strategy, information sources and data collection processes are included in Text S1 in the **Online Supplementary Document**.

### Assessment of study eligibility

We included observational and interventional studies from LMICs involving children aged 28 days to nine years with pneumonia and reporting data on risk factors for death. We focussed on studies published since 2010 to reflect current aetiology and diagnostic / treatment approaches and limited to English language. We included studies involving children older than five years of age as this is an important neglected child population but expected most studies to focus on the traditional under five-year population. Two reviewers (CW and MB) independently screened the titles and abstracts of all returned studies, obtained full text for studies that were screened in by either reviewer, then independently assessed them for inclusion. We resolved disagreements by discussion and, where appropriate, review by a third reviewer (HG). None of the reviewers were blind to the journal titles, study authors, or affiliated institutions.

### Data management, extraction, and synthesis

Two reviewers (CW and MB) independently extracted data from each eligible study and entered data into standardised data extraction spreadsheet using Excel (Microsoft, Redmond, US). We resolved disagreements by discussion and contacted study authors where appropriate to resolve uncertainties. We extracted data on study design, context, population, mortality and treatment failure rates, and associations with various risk factors (categorised as demographic, clinical, and laboratory risk factors).

We categorised context, population and outcome data, then qualitatively synthesised results to determine the consistently reported risk factors for mortality in children with pneumonia including summary data on odds ratios and case fatality rates. To account for the possibility of type 1 or type 2 errors clouding the true significance of factors that were only analysed in a small number of studies, we disaggregated results to show which factors were examined in at least four studies. We focus our reporting on those factors that have been studied in at least four studies and found to be universally (in all studies) or consistently (in the majority of studies) associated with mortality.

We reported crude and adjusted estimates of associations with mortality, as per individual studies, recognising that there was variable use of odds ratios, risk ratios (relative risk), and hazards ratios. To enable comparison between studies we summarised crude estimates (usually odds ratio) for the risk factors identified to be most consistently and strongly associated with death reporting them as median and range. We did not attempt meta-analysis as our primary goal was to understand treatment outcomes with respect to a range of risk factors with variability in definition and heterogeneity in population and context. We did not attempt quantitative examination of heterogeneity or certainty but reported all outcomes of interest by individual study in supplemental material for completeness and transparency.

In addition to mortality as a hard endpoint of treatment failure, we also reviewed other treatment failure endpoints (variously defined as clinical deterioration requiring a change in management, or persistence of particular symptoms), hypoxaemia (an objective sign of respiratory failure), and the need for high dependency unit / intensive care unit (HDU / ICU) admission.

### Assessment of study quality and risk of bias

We assessed the quality and risk of bias of all included studies by using the Effective Public Health Practice Project (EPHPP) Quality Assessment Tool [7,8]. Two reviewers (CW and MB) independently rated studies as strong, moderate, or weak with respect to selection bias, study design, confounders, blinding, data collection method, withdrawals and dropouts, and a global rating. Where disagreements occurred, a third reviewer (HG) carried out a final assessment.

We report our findings according to PRISMA and SWiM standards [9,10].

## RESULTS

**Figure 1:**
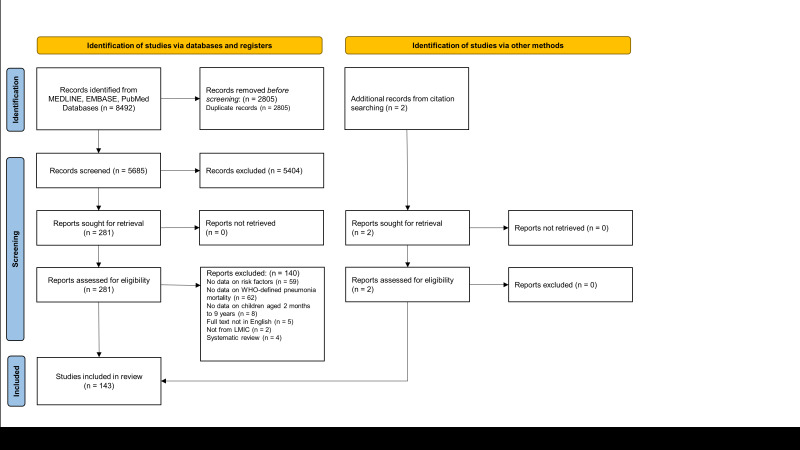
Preferred Reporting Items for Systematic Reviews and Meta-Analyses (PRISMA) flow diagram [9].

A total of 8492 references were retrieved through the search, and two additional relevant papers were identified on review of references. After duplicates were removed 5687 references were screened, including full text screening of 283 publications. We excluded 140 papers on full text review (Text S2 in the **Online Supplementary Document**), primarily due to lack of relevant data on risk factors (n = 59) or not relating to children with pneumonia (n = 50) (**Figure 1**). We included 143 studies in qualitative synthesis [11-153].

### Characteristics of studies

Included study details and participant characteristics are reported in **Table 1** and details are presented in Table S4 in the **Online Supplementary Document**. Papers were included from all WHO regions and all World Bank income levels, including 36 (25%) studies involving low-income countries (LICs), 70 (49%) LMICs, and 53 (37%) upper-middle income countries (UMICs). One hundred and thirty-six (95%) studies were observational, including prospective and retrospective cohort studies, cross-sectional studies, case-control studies, surveillance studies, with seven (5%) interventional studies. Study quality was typically low to moderate with seven (5%) studies rating as “strong” on EPHPP (Table S1 in the **Online Supplementary Document**). Thirty-five (25%) studies addressed risk factors for mortality in childhood pneumonia as their primary aim.

**Table 1:** Characteristics of included studies and participant characteristics

Author, year	Study type	Time period	Setting	Country (city or region)	No. of participants	Median age, months	% male	% severe pneumonia	% SpO_2_<90%	% PICU	No. of deaths	Case fatality rate %
Abdulkadir, 2015 [11]	Cross-sectional study		Urban tertiary hospital	Nigeria (Ilorin)	200	14	60		41		17	8.5
Acuna, 2018 [12]*	Retrospective observational	2004-2016	Urban tertiary hospital	Paraguay (Asuncion)	222	38	63			40 MV	42	18.9
Adewuyi, 2012 [13]	Observational		Urban tertiary hospital	South Africa (Pretoria)	107						18	16.8
Agweyu, 2015 [14]	Randomised control trial	09/2011-08/2013	Six hospitals with paediatric inpatient units including four district hospitals and two provincial general hospitals	Kenya (three sites in central Kenya, three sites in Western Kenya)	527	13	57	6.3			4	0.8
Agweyu, 2018 [15]*	Retrospective cohort	03/2014-02/2016	14 district hospitals	Kenya (14 sites)	16 162	13					18	1.1
Agweyu, 2018 [16]	Retrospective cohort	09/2011-08/2013	Six hospitals with paediatric inpatient units including four district hospitals and two provincial general hospitals	Kenya (three sites in central Kenya, three sites in western Kenya)	1709	12	56	22			832	5.2
Ahmed, 2016 [17]	Prospective cross-sectional	01/2014-12/2014	Urban tertiary hospital	Pakistan (Karachi)	540		61				46	8.5
Ahmed, 2018 [18]	Retrospective cohort	01/2011-12/2014	National referral hospital	Mauritania (Nouakchott)	665		45				120	18.1
Al Amad, 2019 [19]	Retrospective cohort	2011-2016	Two urban hospitals	Yemen (Aden, Sana'a)	1413					40	126	9
Ali, 2013 [20]	Prospective surveillance study	08/2009-09/2011	Tertiary private hospital	Pakistan (Karachi)	812	9.7					13	1.6
Alohan, 2019 [21]	Cross-sectional study	09/2016-09/2017	Three secondary health facilities	Nigeria (southwest)	379	14	48		36		25	6.6
Araya, 2016 [22]*	Retrospective observational	01/2004-06/2013	Urban tertiary hospital	Paraguay (Asuncion)	860	34	56			12, 9 MV	56	6.5
Atwa, 2015 [23]*	Prospective cross-sectional	10/2012-08/2014	Tertiary hospital	Egypt (Fayoum)	242	17	56				9	3.7
Awad, 2020 [24]	Prospective surveillance study	15/1/2016-15/4/2016	Two tertiary referral hospitals	Jordan (Irbid)	479	10.4	63		8.1	10	3	0.6
Awasthi, 2018 [25]	Prospective cohort study with nested case-control	02/2014-06/2016	Urban tertiary hospital	India (Lucknow)	350						24	6.9
Ayieko, 2012 [26]	Cross-sectional study	03/2007-03/2008	Nine rural district hospitals	Kenya (multiple districts)	3372	12	53	16			195	5.9
Azab, 2016 [27]	Case-control	08/2013-10/2015	University hospital (tertiary urban)	Egypt (Zagazig)	100	41	53			25	8	8
Azab, 2014 [28]	Prospective longitudinal cohort study	08/2009-06/2013	University hospital (tertiary urban)	Egypt (Zagazig)	1470	65	61		16		237	16
Barger-Kamate, 2016 [29]	Case-control	08/2011-01/2014	Nine centres	Kenya (Kilifi), Zambia (Lusaka), South Africa (Soweto), Mali (Bamako), The Gambia (Basse), Bangladesh (Dhaka and Matlab), Thailand (Nakhon Phanom and Sa Kaeo)	4200							
Basnet, 2015 [30]	Prospective cohort study	02/2006-06/2008	One tertiary referral urban children's hospital	Nepal (Kathmandu)	610	6	61	49	62	1.1	4	0.7
Bekele, 2017 [31]*	Cross-sectional study		Urban tertiary hospital	Ethiopia (Jimma)	107		54				5	4.7
Benet, 2017 [32]*	Prospective longitudinal	05/2010-06/2013	Five hospitals	India (Lucknow and Vadu), Madagascar (Antananarivo), Mali (Bamako), Paraguay (San Lorenzo)	405	14	58		17		14	3.5
Berkley, 2010 [33]	Prospective observational and case-control study	01/2007-12/2007	Rural district hospital	Kenya (Kilifi)	759	9	59				24	3.2
Bezerra, 2011 [34]	Prospective cross-sectional	04/2008-03/2009	One public teaching hospital	Brazil (Recife, Pernambuco)	407	8	58	10	10		3	0.7
Bills, 2020 [35]*	Prospective observational	06/2016-09/2016	Public ambulances	India (Andhra Pradesh, Assam, Gujarat, Himachal Pradesh, Karnataka, Meghalaya, Telangana)	1433	24	54				94	7.7
Bjorklund, 2019 [36]	Prospective, non-blinded, non-randomised interventional study	04/2015-06/2015, 07/2015-06/2016	One public government referral hospital		83	15.6	53				8	9.6
Bokade, 2015 [37]*	Observational	2010-2012	Tertiary hospital	India	290		66			24	25	8.6
Boukari, 2011 [38]	Retrospective observational	10/2008-10/2010	Urban hospital	Algeria (Blida)	221	11.9	54				7	3.2
Caggiano, 2017 [39]	Prospective observational study		Rural hospital	Tanzania (Itigi)	100	33	47	24			11	11
Champatiray, 2017 [40]	Prospective observational	09/2013-08/214	Urban tertiary hospital	India (Cuttack)	141	5	61	40			31	22
Chisti, 2010 [41]	Retrospective chart review	05/2005-04/2006	Urban tertiary hospital	Bangladesh (Dhaka)	48	3					7	14.6
Chowdhury, 2020 [42]	Case-control	04/2015-12/2017	One urban referral hospital	Bangladesh (Dhaka)	360	8	62				40	11.1
Cohen, 2015 [43]	Prospective surveillance study	02/2009-12/2012	One urban, one periurban and two rural hospitals	South Africa (Gauteng Province, KwaZulu-Natal Province, Mpumalanga Province)	8723			53	35 given O_2_		150	1.8
Cotes, 2012 [44]	Retrospective case study	04/2000-11/2006	One urban paediatric hospital and two urban general hospitals	Colombia (Bogota, Manizales)	535	8	54				19	3.6
Daga, 2014 [45]	Observational		Tertiary care centre	India (Pune)	616						140	22.2
Dembele, 2019 [46]*	Prospective observational	06/2008-03/2016	Two secondary-care hospitals, one tertiary-care hospital and one urban research centre	Philippines (Biliran, Palawan, Manila, Tacloban)	4305						198	4.6
Diez-Padrisa, 2010 [47]	Prospective observational	09/2006-09/2007	Rural district hospital	Mozambique (Manhiça District)	176		64				17	9.7
Divecha, 2019 [48]*	Prospective observational	01/2011-06/2012	Tertiary hospital PICU	India (Mumbai)	293	18.7	62			100, 63 MV	90	30.7
Do, 2011 [49]	Prospective descriptive	11/2004-01/2008	Urban referral hospital	Vietnam (Ho Chi Minh City)	309				12.6	26, 0.3 MV	2	0.6
Durigon, 2015 [50]	Prospective surveillance study	03/2008-02/2010	Urban tertiary referral hospital	Brazil (São Paolo)	622					14, 13 MV	9	1.2
Emukule, 2014 [51]*	Observational	08/2009-07/2012	Rural district hospital	Kenya (Siaya)	3581						218	6
Enarson, 2015 [52]*	Prospective cohort	10/2000-06/2003	16 district hospitals	Malawi	15 709						1633	10.4
Enarson, 2014 [53]	Prospective interventional, non-randomised stepped wedge design	10/2000-12/2005	24 district hospitals including three tertiary referral hospitals	Malawi	47 228						4605	9.8
Evelyn, 2019 [54]	Cross-sectional study	10/2015-12/2017	Urban tertiary hospital	Colombia (Bogota)	420	43	56			24 MV	30	7.1
Ezeonu, 2015 [55]	Retrospective case note review	01/2005-01/2010	Rural tertiary hospital	Nigeria (Abakaliki)	239		58				18	7.5
Fagbohun, 2020 [56]	Observational		Secondary health centres with limited facilities	Nigeria (southwest)	519		50				43	8.3
Fancourt, 2017 [57]	Observational	08/2011-01/2014	Nine hospitals	Kenya (Kilifi), Zambia (Lusaka), South Africa (Soweto), Mali (Bamako), The Gambia (Basse), Bangladesh (Dhaka and Matlab), Thailand (Nakhon Phanom and Sa Kaeo), Madagascar (Antananarivo)	3587			31			373	8.8
Feikin, 2017 [58]	Observational	08/2011-01/2014	Nine hospitals	Kenya (Kilifi), Zambia (Lusaka), South Africa (Soweto), Mali (Bamako), The Gambia (Basse), Bangladesh (Dhaka and Matlab), Thailand (Nakhon Phanom and Sa Kaeo)	1733		56	29				
Ferolla, 2013 [59]	Prospective observational		Urban hospitals	Argentina (Buenos Aires, La Plata)	1293		54		13		22	1.7
Ferreira, 2014 [60]*	Longitudinal, hospital-based observational study,	01/1996-12/2011	Urban tertiary referral hospital	Brazil (Rio de Janeiro)	871		55				26	3
Fischer Langley, 2013 [61]	Prospective, active hospital-based surveillance	11/2007-07/2010	Three referral hospitals	Guatemala (Guatemala City, Queteltenango, Santa Rosa)	2193	9.2					70	3.2
Gallagher, 2020 [62]*	Observational	08/2011-11/2012	Nine hospitals	Kenya (Kilifi), Zambia (Lusaka), South Africa (Soweto), Mali (Bamako), The Gambia (Basse), Bangladesh (Dhaka and Matlab), Thailand (Nakhon Phanom and Sa Kaeo)	1802	9	57				120	6.6
Gowraiah, 2014 [63]	Prospective observational	10/2012-04/2013	Four urban public hospitals - outpatients and inpatients	India (Lucknow, Bangalore)	524	11	64				8	1.6
Graham, 2019 [64]*	Prospective cohort study nested within a stepped-wedge trial	11/2015-10/2017	12 secondary-level hospitals	Nigeria (Southwest)	2073	11	54				33	10.1
Graham, 2011 [65]	Prospective, clinical descriptive		Urban tertiary referral hospital	Malawi (Blantyre)	327				37		149	7.2
Hasan, 2014 [66]	Surveillance	2005-2010	Two provincial, 16 district, two military hospitals (10-140 inpatient beds)	Thailand (Sa Kaeo and Nakhon Phanom provinces)	28 543		59		9, 22 given O_2_	1.1 MV	98	0.3
Hatem, 2019 [67]	Surveillance	02/2010-02/2014	Urban tertiary referral hospital	Egypt (Cairo)	961					18, 6.7 MV	13	1.3
Hooli, 2016 [68]	Retrospective observational	10/2011-06/2014	Seven district hospitals	Malawi (Mchinji and Lilongwe)	14 665						464	3.2
Hutton, 2019 [69]*	Retrospective cohort study	01/2012-12/2012	Urban tertiary hospital, PICU	South Africa (Cape Town)	358	4	59			100, 73 MV	34	12.8
Ibraheem, 2020 [70]	Retrospective descriptive cross-sectional study	01/2013-12/2017	Urban teaching hospital	Nigeria (Ilorin)	971		51				81	8.3
Indriyani, 2018 [71]	Prospective observational	01-2018-10/2018	Urban regional hospital	Indonesia (West Nusa Tenggara Province)	90		57	2.3			24	6.1
Indriyani, 2019 [72]	Retrospective observational	01/2015-12/2016	Urban regional hospital	Indonesia (West Nusa Tenggara Province)	392		59					
Iroh Tam, 2018 [73]	Analysis of data from 2 existing cohort studies	11/2012-12/2013	Urban regional referral hospital	Uganda (Mbarara)	382							
Jain, 2018 [74]*	Prospective observational	08/2014-07/2015	Urban tertiary hospital	India (Lucknow)	152	19.5	61				11	7.2
Jakhar, 2018 [75]	Prospective cohort	10/2012-09/2013	Urban tertiary hospital	India (Delhi)	120	11.9	62				0	
Jroundi, 2014 [76]	Surveillance	10/2010-12/2011	Urban tertiary referral hospital	Morocco (Rabat)	700	21.4	64	27		8	28	4.1
Jroundi, 2014 [77]	Prospective observational	10/2010-12/2011	Urban tertiary referral hospital	Morocco (Rabat)	689	21.4	64				28	4
Jullien, 2020 [78]	Prospective observational	07/2017-06/2018	Urban referral hospital	Bhutan (Thimpu)	189	10.8	58	79	75 given O_2_	16	6	3.2
Kelly, 2015 [79]	Prospective cohort and case-control studies	04/2012-08/2014	Urban referral hospital	Botswana (Gaborone)	310	6	55		61	2.0 MV	14	5.9
Kelly, 2015 [80]	Prospective cohort	04/2012-10/2013	Urban referral hospital	Botswana (Gaborone)	238	6.1	55	34	60	2.0 MV	18	5.8
Kelly, 2019 [81]	Prospective cohort	04/2012-06/2016	Urban referral hospital	Botswana (Gaborone)	390	7.4	57	33	36, 61 given O_2_	3.0 MV	19	5.4
Khuri-Bulos, 2020 [82]	Surveillance	03/2010-03/2013		Jordan	3168	3.5	60		32 given O_2_	9.0, 4.0 MV	31	1
Kim, 2019 [83]	Prospective cohort		Provincial hospital	Vietnam (central)	3817						189 death or ICU	8.6
King, 2015 [84]	Prospective cohort study (nested within a larger parent study assessing the impact of PCV introduction in Malawi)	09/2013-06/2014	Rural primary care and community health workers	Malawi (Mchinji and Lilongwe districts)	769	21.7						
Korkmaz, 2018 [85]	Prospective observational	01/2014-02/2015	Tertiary hospital	Turkey (Samsun)	66	42	59			20, 9.1 MV	1	1.5
Ku, 2020 [86]	Retrospective observational	01/2010-07/2014	Rural emergency department	Uganda (Rukungiri district)	1238							
Kuti, 2013 [87]*	Observational	11/2010-04/2011	Rural referral hospital	Gambia (Basse)	420	18	55				15	3.6
Laman, 2013 [88]*	Observational		Urban tertiary referral hospital	Papua New Guinea (Port Moresby)	77				26		4	5.2
Lanaspa, 2015 [89]	Observational / surveillance	09/2006-09/2007	District hospital	Mozambique (Manhiça)	926	10.5					102	12.2
Lazzerini, 2016 [90]*	Retrospective observational	2001-2012	22 of 23 district hospitals, three of four central hospitals, and 16 of 37 Christian Hospital Association of Malawi facilities	Malawi	113 154						6903	6.6 in 2001, 4.5 in 2012
le Roux, 2015 [91]	Prospective cohort	05/2012-05/2014	Community	South Africa (Cape Town)	109						2	1.4
Lima, 2015 [92]	Prospective and descriptive study	10/2010-09/2013	Teaching hospital	Brazil (Recife)	452		52		48	3.8	7	1.5
Lozano-Espinosa, 2019 [93]	Cohort-type analytical study	08/2017-06/2018	Paediatric referral hospital	Colombia (Bogota)	217			2.4			NA	
Lufesi, 2015 [94]*	Prospective cohort	2001-2012	40 facilities	Malawi (multiple sites)	105 413						6903	6.6
Ma, 2019 [95]	Prospective cohort study	09/2013-07/2015	One regional referral hospital and one district hospital	Uganda (Jinja, Kambuga)	155	11	42				22	14.2
Macpherson, 2019 [96]*	Retrospective cohort	3/2014-02/2018	13 purposely selected public county hospitals, situated in regions of high and low malaria transmission, which are representative of district-level health facilities in Kenya	Kenya (13 sites)	1832	84	56	33	22		145	7.9
Mathew, 2015 [97]	Surveillance	04/2011-03/2013	Urban tertiary hospital	India (Chandigarh)	2345		72	13			108	4.6
McCollum, 2019 [98]	Open-label, randomised, superiority trial	06/2015-03-2018	Rural district hospital	Malawi (Salima)	644	7.7	54				88	13.7
McCollum, 2020 [99]	Case-control	12/2012-01/2014	Hospitals	Kenya (Kilifi), Zambia (Lusaka), South Africa (Soweto), Mali (Bamako), The Gambia (Basse), Bangladesh (Dhaka and Matlab), Thailand (Nakhon Phanom and Sa Kaeo)	618			35			69	11.2
Meligy, 2016 [100]	Prospective descriptive	10/2013-03/2014	Urban university hospital	Egypt (Cairo)	44	9	55		100	73, 50 MV	11	25
Mildemberger, 2017 [101]	Observational	2010-2015	Urban university hospital	Brazil (Curitiba)	184	Approx. 2 y				22		
Mohamed, 2017 [102]	Prospective case-control	01/2016-12/2016	Urban tertiary hospital	Egypt (Minia)	40	12.6	58					
Morrow, 2014 [103]	Prospective observational	11/2006-08/2008	Urban tertiary hospital	South Africa (Cape Town)	202		46		100		51	25
Moschovis, 2015 [104]	Secondary analysis of clinical trial data	08/2000-04/2004	Eight urban tertiary hospitals	Bangladesh (Dhaka), Ecuador (Guayaquil), Pakistan (Multan, Rawalpindi), India (Chandigarh), Zambia (Lusaka), Yemen (Sana’a), Mexico (Mexico City)	958	7.5	62					
Moschovis, 2013 [105]	Secondary analysis of clinical trial data (APPIS and SPEAR trials)	05/1999-04/2004	16 tertiary hospitals	Columbia (Bogota), South Africa (Cape Town), South Africa (Durban), Vietnam (Ho Chi Minh City), Pakistan (Islamabad), Ghana (Kumasi), Mexico (Mexico City), India (Nagpur), Zambia (Ndola), India (Chandigarh), Bangladesh (Dhaka), Ecuador (Guayaquil), Zambia (Lusaka), Mexico (Mexico City), Pakistan (Multan), Pakistan (Rawalpindi), Yemen (Sana’a)	2542	5.4	61		26			
Myers, 2019 [106]	Observational	02/2014-04/2014	Urban tertiary hospital	Malawi (Lilongwe)	62			62			8	12.9
Naheed, 2019 [107]	Surveillance	05/2004-12/2008	Seven tertiary teaching hospitals, six urban, one rural, three government, three private	Bangladesh (Dhaka, Chittagong, Tangail)	6856	10.1	65	20	66	4.7	276	4
Nantanda, 2014 [108]	Prospective observational	08/2011-06/2012	Urban tertiary referral hospital	Uganda (Kampala)	614		57				22	3.6
Nathan, 2014 [109]*	Retrospective observational	11/2010-11/2011	Urban tertiary referral hospital	Malaysia (Kuala Lumpur)	391	8			59	4.3 MV	5	1.3
Negash, 2019 [110]	Prospective observational study	09/2016-08-2017	Two large urban hospitals	Ethiopia (Addis Ababa)	549	9	59				13	2.37
Nemani, 2016 [111]	Prospective observational	07/2013-06/2014	Urban tertiary teaching hospital	India (Lucknow)	135	18.3		25	40			
Nguyen, 2019 [112]	Prospective descriptive	07/2017-06/2018	Urban secondary referral hospital	Vietnam (Da Nang)	4206				11	8.0, 2.0 MV	16	0.4
Nimdet, 2017 [113]	Retrospective cohort	06/2011-06/2014	Urban provincial hospital	Thaialnd (Surat Thani)	135					100	3	2.2
O'Callaghan-Gordo, 2011 [114]	Surveillance	09/2006-09/2007	Rural district hospital	Mozambique (Manhiça District, Maputo Province)	835	11					33	9
Ofman, 2020 [115]*	prospective, population-based, cross-sectional study	2011-2013	Urban hospitals	Argentina (Buenos Aires, La Plata)	664					9.0	15	2.26
Olsen, 2010 [116]	Surveillance	09/2003-08/2005	Two provincial hospital, sixteen district hospitals, two military hospitals in two provinces (mix of urban and rural)	Thailand (Sa Kaeo and Nakhon Provinces)	1325						3	0.23
Onyango, 2012 [117]	Surveillance	01/2007-12/2010	Rural district hospital and health clinics	Kenya (Kilifi)	2429	9	50	21			158	6.5
Orimadegun, 2013 [118]	Cross-sectional study	04/2010-03/2011	Urban tertiary hospital	Nigeria (Ibadan)	333		60		49		33	10.5
Pagano, 2018 [119]	Observational		Urban hospitals x 2	Uganda (Mbarara)	185						6	3.24
Pale, 2017 [120]	Surveillance	01/2015-01/2016	Urban tertiary hospital	Mozambique (Maputo)	450	6	52		4.2		2	0.44
Pedraza-Bernal, 2016 [121]*	Prospective cohort study	01/2014-01/2015	Urban tertiary university hospital	Colombia (Bogota)	416	6	51			21, 16 MV	0	0
Pulsan, 2019 [122]	Prospective observational study	03/2014-08/2016	Urban tertiary referral hospital	Papua New Guinea (Port Moresby)	64	3		75			27	56.3
Rajatonirina, 2013 [123]*	Prospective cohort study	11/2010-07/2012	Urban tertiary referral hospital	Madagascar (Antanarivo)	290	13	56				9	3
Ramachandran, 2012 [124]*	Retrospective chart review	01/2006-12/2008	Urban tertiary referral hospital	India (Chennai)	4375		58				357	8.2
Ramakrishna, 2012 [125]	Analysis of data from a prospective cohort study	07/2005-11/2006	Urban referral hospital	Malawi (Blantyre)	233	11		26			25	10.7
Reed, 2012 [126]*	Secondary analysis of clinical trial data	1998-2001	Urban tertiary hospital	South Africa (Soweto)	4148						298	7.18
Rose, 2010 [127]	Prospective cohort study		Urban paediatric hospital	Brazil (Recife)	457							
Saghafian-Hedengren, 2017 [128]	Nested cohort study in broader surveillance study	04/2011-02/2013	Community and hospital	India (Chandigarh)	196			50			9	4.6
Saha, 2016 [129]*	Surveillance	01/2011-12/2013	Two urban children´s referral hospitals	Bangladesh (Dhaka)	3639			61			63	2
Saleh, 2018 [130]	Prospective cohort study	01/2016-10/2017	Urban university hospital	Egypt (Menoufia)	480		44	67		67 MV	128	26.7
Shan, 2019 [131]*	Retrospective observational	01/2010-12/2014	Urban tertiary children's hospital	China (Suzhou)	28 043		62			2.4	359 (incl. not cured)	1.28
Solis-Chaves, 2018 [132]	Case-control	01/2010-01/2015	Urban tertiary children's hospital	Costa Rica (San Jose)	160	18.2 (mean)	57	50			3	1.9
Srinivasan, 2012 [133]	Randomized double-blind placebo-controlled clinical trial	09/2006-03/2007	Urban national teaching and referral hospital, paediatric ward	Uganda (Kampala)	352	17.9 (mean)	56				28	8
Sudarwati, 2014 [134]	Surveillance	2007-2009	Hospital	Indonesia (Bandung)	160						6	3.75
Suntarattiwong, 2011 [135]	Prospective observational	12/2007-08/2009	Paediatric hospital Thailand (Bangkok)	Thailand (Bangkok)	354	7 (mean)	64		70		3	0.85
Sutcliffe, 2016 [136]	Prospective interventional	10/2011-03/2014	Urban university teaching hospital	Zambia (Lusaka)	693		53				126	18.2
Suzuki, 2012 [137]	Prospective observational	05/2008-05/2009	Tertiary government hospital	Philippines (Tacloban City)	819	9	55	44			88 (incl. 18 likely died at home)	10.7 (incl. 2.2 likely died at home)
Tapisiz, 2011 [138]	Retrospective study	2000-2008	Urban university teaching hospital	Turkey (Ankara)	501	37	55			3.0	1	0.2
Tomczyk, 2019 [139]*	Retrospective review	09/2007-12/2013	Three hospitals (two urban, one rural)	Guatemala (Guatemala City, Queteltenango, Santa Rosa)	4109						174	4
Tuti, 2017 [140]*	Retrospective cohort	02/2014-02/2016	14 public hospitals	Kenya (multiple districts)	10 687		55				252	2.36
Walk, 2016 [141]	Prospective observational	07/2012-09/2012	Urban referral hospital	Malawi (Kamazu)	77	5	43				36	47
Wandeler, 2015 [142]	Prospective observational	06/2002-01/2003	Rural hospital	Senegal (Ndioum)	70	17.4	53		43		7	10
Webb, 2012 [143]	Prospective cohort study	05/2007-05/2008	Rural district hospital	Kenya (Kilifi)	568	11	57	29			34	6
Wilson, 2017 [144]	Open-label, cluster, crossover trial	01/2014-12/2015	Two non-tertiary hospitals (one district hospital, one municipal hospital)	Ghana (Mampong, Kintampo)	2200						70	3.21
Zabihullah, 2017 [145]*	Prospective observational	12/2012-03/2013	700 bed regional referral hospital	Afghanistan (Mazar-e Sharif)	639	5	64				75	12.1
Zampoli, 2011 [146]	Prospective observational	12/2006-06/2008	Urban tertiary referral hospital	South Africa (Cape Town)	202	3.1	45			54	51	25
Zeeshan, 2020 [147]*	Retrospective cohort	01/2013-03/2018	PICU in university hospital	Pakistan (Karachi)	187		65					
Zhang, 2013 [148]*	Prospective observational	01/2007-12/2010	10 bed PICU in urban university children's hospital	China (Suzhou)	10 836	19	57		100		4	6.2
Zhang, 2011 [149]	Prospective, observational	10/2004-10/2005	University hospital, paediatric department	China (Lanzhou)	853	28	56			12, 2.6 MV	5	0.6
Zhang, 2020 [150]	Prospective cohort study	09/2013-07/2015	One district and one regional hospital	Uganda (Jinja, Kambuga)	65	4	63		100		41	5.8
Zhu, 2012 [151]	Prospective descriptive	01/2009-12/2009	23 PICUs	China, 23 urban PICUs	276						72	26.1
Zidan, 2014 [152]	Prospective cohort	05/2011-06/2013	Urban university hospital	Egypt (Zagazig)	100	25	52			27	7	7
Zurita-Cruz, 2020 [153]	Cross-sectional study	01/2013-12/2017	All IMSS health facilities in the country, including 1^st^, 2^nd^ and 3^rd^ level facilities	Mexico	66 304	3.5 (mean)	61			0.5	371	0.56

CAP – community acquired pneumonia, ICU – intensive care unit, MV – mechanical ventilation, PICU – paediatric intensive care unit, IMSS – Instituto Mexicano de Seguro Social (eng. Mexican Institute of Social Security)

*These studies had risk factor identification as an explicit objective.

## DISCUSSION

Number of child participants in each study ranged from 40 to 113 154 (median = 519). Median age ranged from three to 65 months and most studies (n = 83 / 92, 90%) that reported the participant sex included more males than females (percentage of males ranged from 44.50% to 71.70%). Vaccination status was reported in 23 (16%) studies, with 52% to 97% of children fully vaccinated according to national schedules. Many studies were conducted prior to introduction of Hib or pneumococcal conjugate vaccine (PCV) and there was enormous variability in PCV and Hib vaccination status (from 0.00 to 99.40%). Case fatality rates ranged from 0.20% to 56.30% which in part reflects different inclusion criteria and context of the different studies, with some including all pneumonia patients, some only including severe pneumonia patients, and some only patients admitted to ICU.

### Risk factors for mortality

**Table 2** summarises the demographic, clinical, and laboratory factors investigated for association with mortality in included studies, restricted to factors reported in at least four studies. Additional factors studied in three or fewer studies are reported in Tables S2a-S2d in the **Online Supplementary Document**. Most risk factors of interest were reported in a small minority of studies, with only two demographic (age, sex), seven clinical (hypoxaemia, HIV infection, WHO severe pneumonia, severe malnutrition, comorbidities, tachypnoea), and two laboratory or aetiology (viral, bacterial) risk factors reported in more than 10% of included studies.

**Table 2:** Associations of demographic, clinical, and laboratory factors with mortality, ranked by the proportion of studies finding an association (limited to factors reported in ≥4 studies)

Risk factor	Studies showing association, n/N (%)	Median OR/RR/HR* (range)	Median CFR (range)
**Demographic factors**			
Age <12 months	27/41 (66%)	2.62 (1.37-28.5)	8.0% (1.9%-11.9%)
Inadequate immunization	9/14 (64%)	3.33 (1.6-12.3)	28%
Low socio-economic status	4/8 (50%)	2.54 (2.2-2.87)	-
Smoking at home	3/6 (50%)	-	-
Indoor air pollution	2/4 (50%)	2.4	-
Low parental education	4/7 (43%)	1.79 (1.43-4.35)	-
Suboptimal breastfeeding	4/7 (43%)	1.79 (1.67-4.38)	-
Female sex	9/36 (25%)	1.34 (1.13-1.76)	5.4% (4.7%-6.1%)
Crowding at home	0/4 (0%)	-	-
**Clinical signs and comorbidities**			
Decreased conscious state	17/17 (100%)	7.39 (5.1-324.0)	-
Hypoxaemia†	32/34 (94%)	5.52 (1.8-48.1)	17.2% (11.9%-21.2%)
WHO “severe pneumonia”‡	26/28 (93%)	4.25 (1.3-26.5)	15.1% (4.37%-38.6%)
Malnutrition WFAZ<-3	13/14 (93%)	4.0 (2.9-15.5)	11.2% (8.9%-22.5%)
Malnutrition WFHZ<-3	8/9 (90%)	6.2 (2.23-10.0)	29.9% (25%-34.8%)
Cyanosis	7/8 (88%)	3.44 (2.76-4.09)	13.97%
Pallor	6/7 (86%)	6.97 (4.37-9.57)	24.0% (17.8%-30.1%)
Malnutrition WFHZ -2 to -3	6/7 (86%)	2.54 (1.7-6.4)	-
Wheeze	12/14 (86%)¶	0.43 (0.1-2.3)	3.59%
Congenital heart disease	11/13 (85%)	3.83 (2.89-6.25)	36.4% (20%-52.7%)
Tachycardia§	5/6 (83%)**	1.08	-
Malnutrition (severe other)	16/20 (80%)	3.71 (1.71-4.63)	20.4% (8.7%-80%)
HIV infected	23/29 (79%)	3.7 (2.7-12.8)	17.9% (6.6%-40%)
Tachypnoea‖	13/17 (76%)	2.35 (0.7-3.2)	9.2 (8.35%-10.0%)
Convulsions	6/8 (75%)	8.72 (1.73-16.6)	-
Heart failure	3/4 (75%)	-	77.8%
Malnutrition HFAZ<-3	3/4 (75%)	2.3 (1.72-2.5)	-
Comorbidities (any)	14/19 (74%)	5.5 (1.91-18.3)	15.0% (2.04%-21.4%)
Malnutrition WFAZ -2 to -3	10/14 (71%)	2.5 (1.41-9.0)	20.6% (7.6%-86%)
Diarrhoea	5/8 (63%)	1.89 (1.8-2.37)	13.46%
Chest indrawing	8/13 (62%)**	2.57 (0.31-6.8)	9.14% (6.3%-20%)
Anaemia	9/15 (60%)	4.39 (1.99-4.6)	35.3%
Crackles on auscultation	3/6 (50%)	2.2 (1.59-2.6)	14.3% (9.62%-18.9%)
HIV exposed (uninfected)	3/6 (50%)	1.73	-
Asthma	2/4 (50%)¶	0.12 (0.11-0.23)	-
Non-asthma lung condition	2/4 (50%)	1.69	-
Neurodevelopmental condition	2/4 (50%)	6.82 (0.62-8.69)	5.2%
Malaria	6/13 (46%)**	0.84 (0.3-1.52)	11.2% (3.0%-19.3%)
Fever (≥38^○^ Celsius)	4/14 (29%)**	1.94 (0.72-2.3)	7.8%
Ex-low birth weight	2/4 (25%)	-	-
Ex-preterm	1/6 (17%)	2.2	-
Malnutrition HFAZ -2 to -3	1/6 (17%)	2.32 (1.28-2.7)	-
Clinical investigations and aetiology			
Pneumocystis jirovecii	4/4 (100%)	3.57 (1.87-5.26)	52.6% (32.1%-73%)
Bacterial (undifferentiated)	9/22 (41%)	4.01 (2.88-4.6)	16.7% (3.5%-24%)
Viral (undifferentiated)	5/16 (31%)¶	0.5	0.05% (0.0%-0.1%)
*RSV*	5/10 (50%)¶	0.33 0.09-0.6)	-
*Influenza (undifferentiated)*	0/4 (0%)	-	-
*Adenovirus*	1/4 (25%)	4.18	-
*Parainfluenza*	2/4 (50%)	12.75 1.9-23.6)	-
*Human metapneumovirus*	2/4 (50%)¶	0.34 (0.25-0.5)	-
CXR consolidation	7/9 (78%)	4.40 (2.6-4.9)	13.5%
Leukopenia (low WCC)	4/4 (100%)	2.34 (2.12-6.5)	-
Leucocytosis (high WCC)	5/9 (56%)**	0.96 (0.15-1.77)	19.2%
Raised CRP	2/6 (33%)	1.14 (0.84-1.44)	-

CFR – case fatality rate, CRP – C-reactive protein, CXR – chest x-ray, HFAZ – height for age z-score, HIV – human immunodeficiency virus, HR – hazards ratio, OR – odds ratio, RR – risk ratio, RSV – respiratory syncytial virus, SpO_2_ – peripheral blood oxygen saturation, WCC – white cell count, WFAZ – weight for age z-score, WFHZ – weight for height z-score, WHO – World Health Organization

*Median OR/RR/HR and CFR reported where available, with ranges if reported in more than a single study.

†Estimates restricted to studies which defined hypoxaemia as SpO_2_<90%.

‡WHO severe pneumonia includes a composite of any of the following clinical signs: SpO_2_<90%, severe respiratory distress, altered conscious state, unable to feed/drink, convulsions, severe malnutrition, severe anaemia/pallor.

§Tachycardia typically defined as heart rate >160/minutes aged <12 months, >120/minutes aged 1-4 years, >100/minutes aged over 5 years.

‖Tachypnoea typically defined by age-specific respiratory rate: ≥60 aged <2 months, ≥50 aged 2-11 months, ≥40 aged 1-4 years, and ≥30 aged over 5 years.

¶Predominate negative association with mortality.

**Mixed positive and negative association.

#### Demographic and household factors

The demographic factors found to be most clearly associated with pneumonia mortality included younger age and inadequate immunization (**Table 2**). Data from 27 / 41 (66%) studies reporting on risks of death by age showed higher risk of death for younger children. Children aged under one year of age had a 2.5-fold higher risk of death (range from 1.4 to 29) than children aged one-four years and risk of death for infants under six months of age was particularly high. Fewer studies reported on the association between immunisation status and pneumonia mortality (n = 14), suggesting that under-immunised children have a 3-fold higher risk of death (range from 1.6 to 12.3) than fully immunised.

Female sex, inadequate breastfeeding, lower parental education level, lower socio-economic status, smoking exposure at home, and indoor pollution at home were all examined in at least four studies and found to be associated with mortality in up to half of those studies (**Table 2**). Data from eight studies reported that children from poorer families had a 2-fold higher risk of death (range from 2.2 to 2.9) than children from wealthier families, with similar increased risk for those with low parental education level compared to higher education (range from 1.4 to 4.4). Half the studies reporting smoking at home (n = 3 / 6) or indoor air pollution (n = 2 / 4) found an association with increased mortality. Suboptimal breastfeeding was found to be associated with mortality in infants with pneumonia in 4 / 7 (43%) of studies with fold higher risk of death (range from 1.7 to 4.4). Girls with pneumonia were found to have an approximate 30% increased risk of death compared to boys with a positive association between sex and mortality reported in one-quarter or 25% of studies (n = 9 / 36) that evaluated an association. This association between sex and mortality was found in diverse studies from Africa, Asia, and South America, with the elevated risk of death typically remaining after adjusting for other factors (e.g. age). Crowding at home was examined in four studies which did not find an association with pneumonia mortality.

Other demographic factors that were found to be associated with mortality in at least one study but reported in three or fewer studies included: low maternal age, past experience of child loss, living in a malaria endemic area, poor quality drinking water, late referral, lack of adequate sewage or latrine at home, and living in a rural area (Table S2a and Table S3a in the **Online Supplementary Document**). Antenatal care and birth spacing was only examined in one study and was associated with a lower risk of mortality.

#### Clinical factors

**Figure 2:**
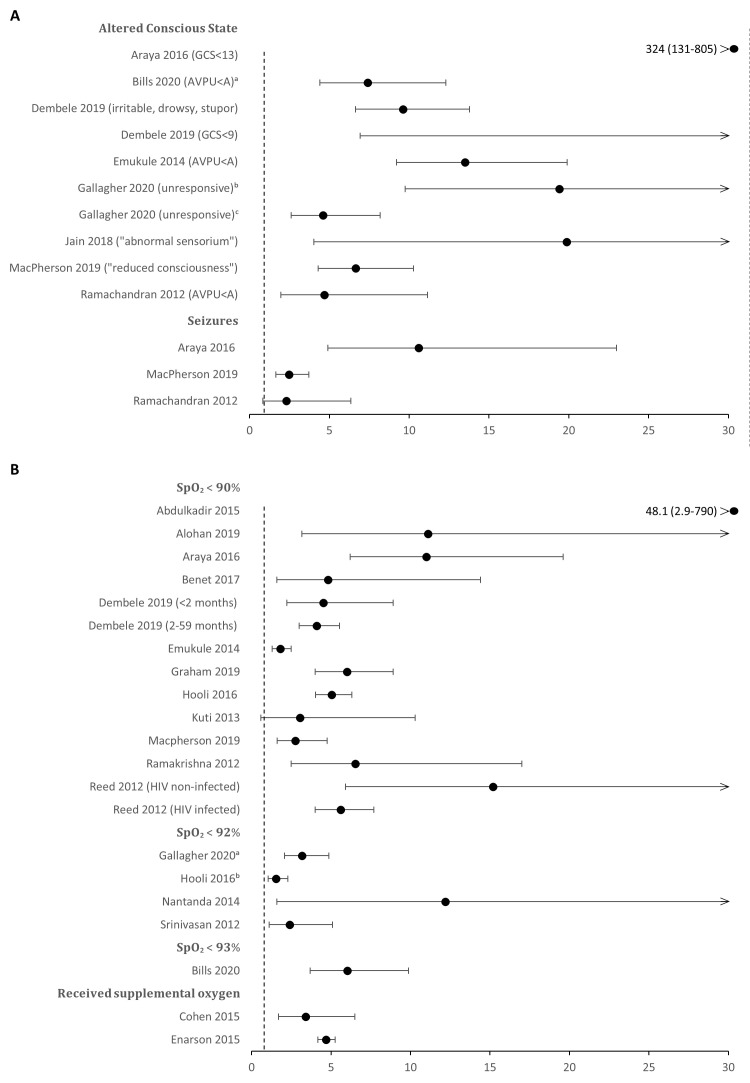
Forest plot showing the unadjusted odds ratios (OR) for death among children with pneumonia from studies in low and middle-income countries (LMICs), comparing those with and without altered conscious state in **Panel A** or hypoxaemia in **Panel B**.

The clinical signs that were most clearly and strongly associated with mortality were decreased conscious state, hypoxaemia, cyanosis, malnutrition, severe pallor, and the presence of comorbid conditions (including HIV infection) (**Table 2**). Decreased conscious state was found to be associated with increased risk of death in all 12 studies that reported, associated with 7-fold higher risk of death (range from 3.1 to 324), with similar estimates from the fewer studies reporting on convulsions (**Figure 2**, panel A). Severe hypoxaemia (SpO_2_<90%) was associated with a 5.5-fold increased risk of death (range from 1.8 to 48.1) in 32 / 34 (94%) studies that reported it, while less severe hypoxaemia (e.g. SpO_2_ = 90%-93%) was also consistently associated with mortality (**Figure 2****,** panel B, Table S3b in the **Online Supplementary Document**).

**Figure 3:**
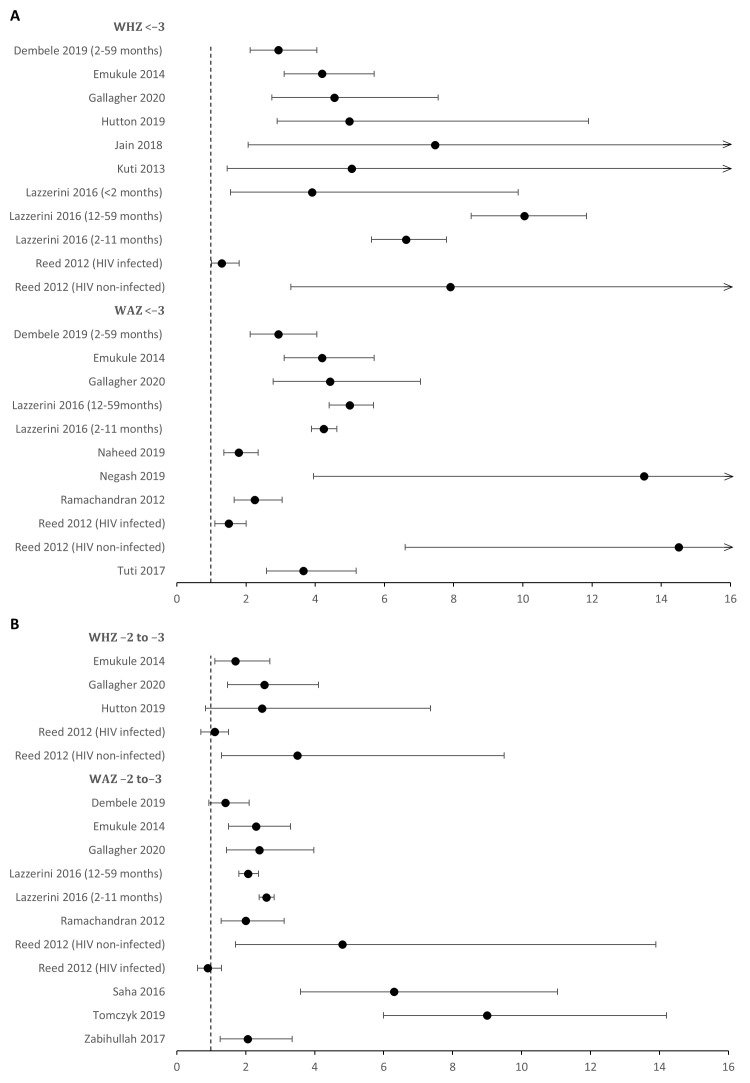
Forest plot showing the unadjusted odds ratios (OR) for death among children with pneumonia from studies in low and middle-income countries (LMICs), comparing those with and without severe acute malnutrition in **Panel A** or moderate acute malnutrition in **Panel B**.

Severe acute malnutrition (based on weight for age / height) was found to increase risk of death 5-fold, while moderate wasting and stunting were also consistently associated with a 2-fold increase in risk of death (**Figure 3**, panel A and panel B, **Table 2**). The WHO “severe pneumonia” classification (cough or difficult breathing with hypoxaemia, SpO_2_<90%, or any danger sign – altered conscious state, convulsions, inability unable to feed/drink), was associated with a 4-fold increase in risk of death in 27 / 29 studies that reported it.

The presence of any chronic condition or comorbidity increased risk of death 5-fold (range from 1.9 to 18), with particular conditions such as congenital heart disease, HIV infection, neurodevelopmental conditions, anaemia, pre-term / low birth weight, being of particular concern. Conversely, the presence of asthma (or wheeze) was generally found to be associated with lower risk of death, with mixed findings about the impact of malaria co-infection. The presence of tachypnoea (fast breathing), diarrhoea, chest indrawing, and positive findings on chest auscultation were each found to be associated with a 2 to 3-fold increased risk of death. Fever and tachycardia were associated with increased risk of mortality in some studies, and decreased risk in others.

Other clinical factors that were found to be associated with mortality in at least one study but reported in three or fewer studies included: hypothermia *t* < 36.50, hypotension, signs of shock, meningitis, previous pneumonia admission, maternal disease in pregnancy, maternal tuberculosis, sepsis and stridor (Table S2a and Table S3b in the **Online Supplementary Document**).

#### Clinical investigations and aetiology

We generally found weak or inconsistent associations with mortality for aetiology other investigations (**Table 2**). While some studies found increased risk of death from bacterial causes of pneumonia and / or decreased risk of death from viral causes, most studies that explored the contribution of bacterial vs viral aetiologies found no association with risk of death. Of the individual bacterial aetiologies, the presence of PJP was found to be associated with 4-fold increase in risk of death (range from 1.9 to 5.3), from a small number of studies (n = 4). Of the viral aetiologies, there is some evidence for increased risk of death from parainfluenza and decreased risk of death from respiratory syncytial virus (RSV) and human metapneumovirus. Consolidation or pleural effusion on chest x-ray (CXR) were associated with a 4-fold increased risk of death compared to children with normal CXR. Data from a small number of studies suggested that high white cell count (leucocytosis) was not associated with mortality but low white cell count (leukopenia) was associated with increased risk of death. We found mixed findings regarding the association between mortality and elevation in C-reactive protein (CRP), a widely used inflammatory marker.

Other aetiological or clinical investigation factors that were found to be associated with mortality in at least one study but were examined in three or fewer studies included: pneumococcus, staphylococcus, influenza A, influenza B, H1N1 influenza, pneumothorax on CXR, electrolyte abnormality, hypoglycaemia, hyperglycaemia, thrombocytopaenia, low zinc, high lactate, acidaemia, low serum bicarbonate, and various markers of infection or inflammatory response (high procalcitonin, high ESR, high IL-1RA, high IL-6, high IL-8, high IL-17, high MIP-1a, high CK-MB fraction, high copeptin, high angiopoietin 2:angiopoietin 1 ratio, low angiopoietin 1, and low CCL22) (Table S2a, Table S3e and Table S3f in the **Online Supplementary Document**).

### Risk factors for other types of severe treatment failure

Tables S2b-2d in the **Online Supplementary Document** summarise the associations between risk factors and hypoxaemia, intensive care unit (ICU) admission, and other forms of treatment failure for all factors in which the association with mortality was reviewed in at least four studies. Additional details are presented in Tables S3g-S3x in the **Online Supplementary Document**.

The numbers of papers reviewing these other endpoints was lower (which may reflect the search strategy’s focus on the primary endpoint of mortality). While there was general concordance in whether different factors were associated each of these endpoints, there were some notable differences. For example, while girls tended to have higher mortality than boys, sex was not associated with hypoxaemia or treatment failure, and girls were less likely to be admitted to ICU than boys. Similarly, while viral aetiology (e.g. influenza, RSV) was associated with hypoxaemia, it was not associated with ICU admission or other treatment failure. Among the clinical risk factors, hypoxaemia, tachypnoea, chest indrawing, and the presence of comorbidities were all consistently associated with hypoxaemia, ICU admission, and other treatment failure. Malnutrition was not clearly associated with hypoxaemia but was associated with ICU admission and other treatment failure, perhaps indicating that children with malnutrition present with similar pneumonia severity but have worse disease progression and recovery.

## DISCUSSION

Our systematic review identified important risk factors for treatment failure or mortality in children with pneumonia. We found many factors that may be associated with mortality, including demographic, maternal, socioeconomic, environmental, laboratory, and radiological factors. The most consistently demonstrated risk factors for child pneumonia death are almost all clinical – young age, inadequate immunization, hypoxaemia, altered conscious state, malnutrition, anaemia, and comorbid conditions. However, clinical risk factors were also the most commonly reported and it is possible that other less-studied risk factors could emerge as important in future studies.

Our findings share similarities to Sonego et al.’s (2015) review of risk factors for child pneumonia mortality [154]. While Sonego et al. included studies as far back as 1988 (n = 61/77 were published before 2010), we focussed on recent studies (published since 2010), identifying many papers not included in the Sonego review and excluding some that the Sonego review included (mostly because they restricted to a narrow population, such as RSV or HIV-infected). Like our review, the Sonego review identified young age, comorbid chronic conditions, malnutrition, unimmunised status, and Pneumocystis jirovecii as major risk factors for child pneumonia death. Two of the major clinical risk factors we identified (hypoxaemia and altered conscious state) were not examined in the Sonego review, with a subsequent review focussed on hypoxaemia clearly confirming it as a major risk factor for death [155]. Compared to the Sonego review, we found less consistent association between socio-economic status, smoking or indoor air pollution and child pneumonia mortality. Both our review and Sonego’s review found evidence of girls with pneumonia having poorer outcomes than boys, possibly related to differential care-seeking and treatment practices, although results from individual studies were mixed. The Sonego review did not explore laboratory and radiological predictors of mortality or individual clinical signs.

### Implications for policy and practice

Many child deaths are predictable based on a few specific risk factors and there are increasing calls to improve risk stratification of existing child health guidelines [1]. Our review highlights risk factors for mortality among children with pneumonia and presents opportunities to improve current guidance and strategies for pneumonia control.

Current pneumonia control strategies identify particular priorities for protection (breastfeeding, supplemental feeding), prevention (immunization, handwashing, HIV-prevention, reduced household air pollution) and treatment (care seeking, case management, antibiotics, oxygen) [156]. Our review supports an emphasis on prevention and treatment of malnutrition and the critical role of immunization and HIV-prevention. While factors such as indoor air pollution and breastfeeding did not emerge as major risk factors for death among children with pneumonia, they remain important in reducing pneumonia incidence in the first place. In addition, we urge added attention to the prevention and treatment of anaemia, and the identification and preventive management of other chronic conditions and comorbidities such as congenital and neurodevelopmental conditions.

In terms of diagnosis and treatment, our review highlights clear opportunities to improve the identification of children with pneumonia who are at high risk of death. First, while malnutrition and hypoxaemia are included in the WHO severe pneumonia classification, current clinical guidelines generally focus on severe forms – severe acute malnutrition (SAM) and SpO_2_<90%. Our review shows than even moderate malnutrition and hypoxaemia (SpO_2_<94%) are associated with increased mortality. Furthermore, conscious state, nutritional status, and blood oxygen levels are poorly assessed in routine clinical care. Routine pulse oximetry, anthropometric measurement (e.g. MUAC), and assessment of conscious state (e.g. AVPU) is critical to identify high-risk patients.

Second, chronic underlying health conditions are not routinely assessed in current treatment algorithms and guidelines, which have traditionally focused on acute infectious killers. Chronic conditions are common and important to be addressed in their own right [157], but should also be considered a major risk factor for children presenting with acute illness. So, while continued emphasis on particular conditions such as malnutrition, anaemia, and HIV infection is warranted, guidelines should also encourage routine identification of any chronic illness or comorbidity and recognise that this puts children at higher risk of poor outcome.

Third, anaemia is a major risk factor for death but is difficult to detect clinically with current WHO guidelines relying on the clinical finding of “severe pallor”. Technology exists for point-of-care haemoglobin measurement, including non-invasive devices integrated with pulse oximetry [158]. Increased access to low-cost point-of-care haemoglobin tools may be particularly useful in malaria-endemic settings where anaemia is particularly common and deadly.

Future research may further define the role of additional laboratory investigations, such as lactate or procalcitonin, in defined populations (e.g. HDU/ICU).

Improved risk assessment and stratification has potential benefits for patients, health care workers, and broader health systems and communities. Severely ill patients can benefit from more prompt and timely diagnosis and access to appropriate treatment. Healthcare workers can more easily decide where to care for patients (e.g. inpatient, outpatient, HDU/ICU), how frequently to monitor and review patients, and when to escalate therapy. Health managers can make better decisions about which populations should be cared for in what level of facility and allocate resources accordingly. The wider community can have greater confidence that their loved ones will be cared for according to need.

### Limitations

We used broad search criteria to capture studies reporting a wide range of potential risk factors for child pneumonia death and included studies from diverse geographical contexts. However, the quality of included studies was generally low and many risk factors were only examined in a small number of studies and limited geographical contexts. For reporting integrity we have included all results in supplemental material but focused our results on risk factors that have been consistently reported in multiple studies. It is possible that risk factors that have not been well explored in the literature to date will emerge with more data. Most included studies were hospital based, with over-representation of large urban hospitals. A high proportion of pneumonia deaths in low- and middle-income countries happen outside of hospital and examining pneumonia at the community or primary care level may reveal different risk factors or risk estimates [1,5]. Furthermore, by only comparing risk factors between patients who have already been admitted to hospital we risk over- or under- emphasising factors that are commonly used to decide about admission (e.g. chest indrawing). Most included studies used the WHO clinical case definition for pneumonia, with few including radiographic evidence or distinguishing pneumonia from conditions such as bronchiolitis or asthma. We know that clinical pneumonia overlaps in features with other common conditions, such as malaria, and this can result in both over-diagnosis and under-diagnosis [159]. However, this represents the case mix reality faced in many health facilities in low- and middle-income countries where clinical features must be relied upon. Our study reviewed the association with mortality among patients with pneumonia, and therefore factors which increase the risk of acquiring pneumonia in the first place (and therefore lead to an increased lifetime risk of dying from pneumonia) may not be highlighted.

## CONCLUSIONS

Many studies of children with pneumonia in LMICs have investigated the association between clinical factors and mortality. A smaller number of studies have investigated sociodemographic, epidemiological and laboratory factors and their relationship to mortality.

Hypoxaemia (low blood oxygen level), decreased conscious state, severe acute malnutrition, and the presence of an underlying chronic condition were the risk factors most strongly and consistently associated with increased mortality in children with pneumonia. Additional important clinical factors that were associated with mortality in the majority of studies included particular clinical signs (cyanosis, pallor, tachypnoea, chest indrawing, convulsions, diarrhoea), chronic comorbidities (anaemia, HIV infection, congenital heart disease, heart failure), as well as other non-severe forms of malnutrition. Important demographic factors associated with mortality in the majority of studies included age <12 months and inadequate immunisation. Important laboratory and investigation findings associated with mortality in the majority of studies included: confirmed PJP, consolidation on chest x-ray, pleural effusion on chest x-ray, and leukopenia. Several other demographic, clinical and laboratory findings were associated with mortality less consistently or in a small numbers of studies.

Routine assessment of blood oxygen levels, nutritional status, conscious state and comorbidities are critical for effective risk assessment and clinical decision making. The strong association of HIV, anaemia, comorbidities, and malnutrition with mortality highlights the importance of good quality primary and preventative care, and early care seeking.

## Additional material


Online Supplementary Document
