# Supplementary material for: What are the risk factors for death among children with pneumonia in low- and middle-income countries? A systematic review
Source: J Glob Health. 2023 Feb 24;13:05003. doi: 10.7189/jogh.13.05003 (PMC9951126; doi:10.7189/jogh.13.05003)
Supplement: Online Supplementary Document [file jogh-13-05003-s001.pdf]

ONLINE SUPPLEMENTAL MATERIAL

Title: What are the risk factors for death among children with pneumonia in low and middle income countries? A systematic review

Authors: Chris Wilkes, Mohamed Bava, Hamish Graham, Trevor Duke, for the ARI Review group

CONTENTS

Text S1 – Further details of study methods including search strategy, data extraction and data management 3

Text S2 – Studies excluded at full-text review..... 10

Table S1 – EPHPP quality assessment of included studies 18

Table S2 – Extended results tables showing summary associations of demographic, clinical and laboratory factors with mortality, treatment failure, hypoxaemia and ICU admission. 22

Table S2a - Summary of number of studies showing positive, no, or negative correlation of specific factors with MORTALITY..... 23

Table S2b – Summary of number of studies showing positive, no, or negative correlation of specific factors with TREATMENT FAILURE 28

Table S2c – Summary of number of studies showing positive, no, or negative correlation of specific factors with HYPOXAEMIA..... 31

Table S2d – Summary of number of studies showing positive, no, or negative correlation of specific factors with ICU ADMISSION 34

Table S3 –Extended results tables showing associations of demographic, clinical and laboratory factors with mortality, treatment failure, hypoxaemia and ICU admission, by individual study..... 37

Table S3a – Associations of mortality with demographic features 38

Table S3b - Associations of mortality with clinical features..... 44

Table S3c - Associations of mortality with comorbidities 54

Table S3d - Associations of mortality with markers of malnutrition..... 63

Table S3e - Associations of mortality with aetiology and laboratory findings..... 68

Table S3f - Other investigated factors for associations with mortality..... 77

Table S3g – Associations of treatment failure with demographic features..... 82

Table S3h - Associations of treatment failure with clinical features..... 84

Table S3i - Associations of treatment failure with comorbidities 86

Table S3j - Associations of treatment failure with markers of malnutrition..... 88

Table S3k - Associations of treatment failure with aetiology and laboratory findings 90

Table S3l - Other investigated factors for associations with treatment failure 91

Table S3m – Associations of hypoxaemia with demographic features..... 92

Table S3n - Associations of hypoxaemia with clinical features 93

Table S3o - Associations of hypoxaemia with comorbidities 94
Table S3p - Associations of hypoxaemia with markers of malnutrition 95
Table S3q - Associations of hypoxaemia with aetiology and laboratory findings 96
Table S3r - Other investigated factors for associations with hypoxaemia 98
Table S3s – Associations of ICU admission with demographic features 99
Table S3t - Associations of ICU admission with clinical features 101
Table S3u - Associations of ICU admission with comorbidities 103
Table S3v - Associations of ICU admission with markers of malnutrition 104
Table S3w - Associations of ICU admission with aetiology and laboratory findings 105
Table S3x - Other investigated factors for associations with ICU admission 107
Table S4 – Additional characteristics of studies 108

Text S1 – Further details of study methods including search strategy, data extraction and data management

PICOTS

- Population – Children aged 28 days - 9 years in low and middle income countries (as defined by the World Bank) with acute lower respiratory tract infection.
- Intervention – Any
- Comparison – Routine care, either following WHO or local guidelines, or study protocol.
- Outcomes addressed – Primary outcome is mortality, defined as mortality in children during or within 30 days of an episode of ALRI. Secondary outcomes are treatment failure as defined by the paper, hypoxaemia, and ICU admission.
- Time Period – 2010 to present
- Setting - Hospitals and community

Study types – All relevant published interventional and observational studies, including randomised controlled trials, cohort studies, case control, cross sectional and relevant case series.

Information sources
Literature search strategies were developed using medical subject headings (MeSH) and text words related to mortality, pneumonia and lower respiratory tract infection, and children. We searched MEDLINE (OVID interface), EMBASE (OVID interface) and PubMed for all relevant published papers.
We scanned the reference lists of included studies and relevant reviews identified through the search.

Table 1. Inclusion and exclusion criteria for studies in this review

Inclusion criteria	*Exclusion criteria*
1. Observational or interventional study or meta-analysis involving original data or analysis<br>2. Published in the year 2010 or later<br>3. Published in English<br>4. Includes children aged between 28 days and 9 years of age and it is possible to extract data specifically relating to children within these age groups from the data available.<br>5. Included children whose primary presentation is pneumonia, as defined by WHO criteria, which may include other types of acute respiratory infection such as bronchiolitis<br>6. One or more children with a primary presentation of pneumonia died, and it is possible to extract data specifically relating to causes or risk factors for death in these children, relative to children with a diagnosis of pneumonia who did not die.<br>7. Wholly or partially undertaken in low or middle-income countries (LMICs), defined by World Bank	1. Does not provide original data or analysis (e.g. review articles, editorials)<br>2. Conducted in a neonatal unit/neonatal ICU, or focuses only on neonates below 40 weeks' gestation<br>3. Study only includes or examines mortality risk factors for patients diagnosed with one particular aetiological cause of pneumonia, rather than undifferentiated pneumonia presentations<br>4. Study only includes a subset of children with known underlying conditions or predisposing factors (e.g. only includes oncology / haematology patients)

Search strategy

Both qualitative and quantitative studies were be sought. No study design or language limits were imposed on the search. Medline, EMBASE, and PubMed were searched for studies from 2010 onwards. The specific search strategies were created by a Health Sciences Librarian with expertise in systematic review searching. The MEDLINE strategy was developed with input from the project team. MEDLINE search strategy is included below. After the MEDLINE strategy is finalized, it was adapted to the syntax and subject headings of the other databases.

Data management

All search results were saved in a shared file accessible to all members of the study team, and citations saved in EndNote and/or Covidence. When reviewing papers for inclusion and exclusion records accessible to all team members were kept in the shared folder and on password protected computers.

Selection process

The titles and abstracts of all papers identified though the search were reviewed by two independent reviewers for inclusion or exclusion according to the above criteria. We obtained full reports for all titles that appeared to meet the inclusion criteria or where there was any uncertainty.

Two independent reviewers then screened the full text reports and decided whether these met the inclusion criteria. Any disagreements were resolved through discussion with the team. Reasons for excluding studies were documented. Neither of the review authors were blind to the journal titles or to the study authors or institutions.

Data collection process

We had a standardised form of specific items (see below) with which 2 independent reviewers extracted data from each eligible study and entered into an excel spreadsheet. Reviewers resolved disagreements by discussion, and unresolved disagreements discussed with the whole team.

Data Items

For all papers included in the study we reviewed the following information:

Size of study (how many patients, how many centres)

Type of study

Year of study (date range)

Purpose of study

Inclusion criteria

Exclusion criteria

Median age of patients included

Age range of patients included

Setting

- Geographic location(s) – WHO region, country, sub-national region
- Urban or rural or both
- Hospital or community or both
- Rates of Hib / pneumococcal vaccination in population studied

First line treatment

Number of deaths

Mortality rate of enrolled patients

Outcomes

For all papers included in the study the primary outcome reviewed was what risk and potential causative factors were identified in patients who died.

Factors reviewed were:

Demographic & Socio-economic:

Age

Sex

Breastfeeding

Maternal Age

Maternal education level

Smoke exposure at home

Indoor air pollution

Socio-economic status

Distance to nearest health facility

Distance to nearest hospital

Clinical:

Severe pneumonia (WHO criteria)

Non-severe pneumonia (WHO criteria)

Temperature ≥38°C

Temperature ≥39°C

Apnoea

Poor feeding

Severe chest in-drawing

Tachycardia

Respiratory Rate>70

Cyanosis

SpO2 <92%

SpO2 <80%

Chest x-ray done

- Radiographic signs present
- Consolidation
- Other

Cough

Wheeze

Stridor

Other documented auscultation findings

Weight for age < -3 SD

Weight for age –2 to -3 SD

Weight for age ≥–2 SD

Weight for age –2 to -3 SD

Weight for age ≥–2 SD

Pallor (any)

Dehydration
- Some dehydration
- Severe dehydration

Days of illness prior to presentation

Days of antibiotic use prior to presentation

Comorbidities:
- Anaemia
- HIV infected
- HIV exposed but uninfected
- Other immunodeficiency
- Cardiac
- Severe malnutrition
- Malaria
- Diarrhoeal illness
- Neurodevelopmental
- Chronic respiratory disease (e.g. bronchiectasis)

Medical history (other than above):

Vaccination status

Ex-preterm

Ex-low-birth-weight

History of asthma

History of previous hospitalisations with pneumonia

Other significant medical history (specify if possible)

Biomarkers:

White cell count

Neutrophil count

CRP

Procalcitonin

Lactate

Aetiological:

Identified organisms

Antibiotic resistance patterns

Break down by
- collection method (NPA, Induced sputum, BAL, Aspiration, blood)
- identification method (PCR, Culture, serology)

Secondary outcomes reviewed were risk factors associated with and likely causes of treatment failure as defined by the paper being reviewed, risk factors for hypoxaemia and for ICU admission. These were reviewed according to the same criteria as for mortality.

Risk of Bias in individual studies

Included studies may or may not have a comparison group. To assess the quality of and risk of bias within included studies, the methodological quality of potential studies was assessed by using the Effective Public Healthcare Panacea Project (EPHPP) QA Tool. Using this tool, studies were rated as strong, moderate or weak with respect to selection bias, study design, confounders, blinding, data collection method, withdrawals and dropouts, and a global rating assigned. This was undertaken by two separate reviewers. Where there was disagreement, a third reviewer was used as an arbitrator.

Medline search strategy:

1. exp *Pneumonia/
2. ((respiratory adj3 (infection* or distress or failure or disease* or illness*)) or pneumonia or pneumonias or lung-inflammation* or lobitis or nonspecific-inflammatory-lung-disease* or peripneumonia or pleuropneumonia or pleuropneumonitis or pneumonic-lung* or pneumonic-pleurisy or pneumonic-pleuritis or pneumonitides or pneumonitis or pulmonal-inflammation* or pulmonary-inflammation* or pulmonic-inflammation* or bronchiolitis).tw,kf.
3. *Pneumococcal Infections/
4. exp *Bronchiolitis/
5. 1 or 2 or 3 or 4
6. *risk/ or exp *risk assessment/ or *risk factors/
7. (risk or risks or determinant* or predict* or cause* or causal* or causative or etiolog* or aetiolog* or odds or variation* or clinical-factor*).tw,kf.
8. et.fs.
9. odds ratio/
10. 6 or 7 or 8 or 9
11. exp *mortality/
12. *Death/ or *infant death/
13. mo.fs.
14. (mortalit* or death* or surviv* or fatal*).tw,kf.
15. 11 or 12 or 13 or 14
16. (infan* or toddler* or pre-schooler* or preschooler* or kinder or kinders or kindergarten* or kinder-aged or boy or boys or girl or girls or child or children or childhood or youngster* or kid or kids or pediatric* or paediatric* or school-age* or schoolage* or schoolchild* or schoolgirl* or schoolboy*).tw,kf,hw.
17. developing countries/
18. (austere or (limited adj2 resource*) or (low adj2 resource*) or (transitioning adj econom*) or (third adj world) or LMIC or LMICs or (lami adj countr*) or (transitional adj countr*) or (low adj gdp) or (low adj gnp) or (low adj gross adj domestic) or (low adj gross adj national) or ((emerging or developing or (low adj income) or (middle adj income) or (low adj3 middle) or underdeveloped or under-developed or (less* adj developed) or underserved or under-served or deprived or poor*) and (countr* or nation*1 or econom* or population or world))).tw,kf.
19. exp africa/
20. americas/ or exp caribbean region/ or exp central america/ or latin america/ or mexico/ or exp south america/

21. europe/ or exp europe, eastern/ or exp transcaucasia/
22. antarctic regions/ or exp atlantic islands/ or exp indian ocean islands/ or exp pacific islands/
23. New Guinea/
24. asia/ or exp asia, central/ or asia, southeastern/ or borneo/ or cambodia/ or east timor/ or indonesia/ or laos/ or malaysia/ or mekong valley/ or myanmar/ or philippines/ or thailand/ or vietnam/ or asia, western/ or bangladesh/ or bhutan/ or india/ or middle east/ or afghanistan/ or iran/ or iraq/ or jordan/ or lebanon/ or oman/ or saudi arabia/ or syria/ or turkey/ or yemen/ or nepal/ or pakistan/ or sri lanka/ or far east/ or china/ or tibet/ or exp korea/ or mongolia/
25. (Afghanistan or Albania or Algeria or Angola or Antigua or Argentina or Armenia* or Aruba or Azerbaijan or Bahrain or Bangladesh or Barbados or Barbuda or Belarus or Byelarus* or Byelorussian or Belorussian or Belorus* or Belize or Benin or Bhutan or Bolivia or Bosnia or Botswana or Brasil or Brazil or Bulgaria or (Burkina adj Fas*) or (Upper adj Volta) or Burma or Burundi or Cambodia or Khmer or Kampuchea or Cameron* or Cameroon* or (Cape adj Verde) or (Cabo adj Verde) or (Central adj African adj Republic) or Chad or Chile or China or Colombia or Comoros or (Comoro adj Island*) or Comores or Mayotte or Congo or Kongo or (Cook adj Island*) or (Costa adj Rica) or (Cote adj D'ivoire) or Croatia or Cuba or Cyprus or Czech* or Djibouti or Dominica or Dominican or (East adj Timor) or (East adj Timur) or Ecuador or Egypt or El-Salvador or (Equatorial adj Guinea) or Eritrea or Estonia or Ethiopia or Fiji or (French adj Somaliland) or Futuna or Gabon or (Gabonese adj Republic) or Gambia or Gaza or (Georgia* adj Republic) or Ghana or Grenada or Guam or Guatemala or Guinea or Guiana or Guyana or Haiti or Herzeg* or Hercegovina or Honduras or Hungary or India or Indonesia or Iran or Iraq or (Ivory adj Coast) or Jamaica or Jordan or Kazakh* or Kenya or Kiribati or Korea or Kosovo or (Kyrgyz adj Republic) or Kyrgyzstan or Kirghizia or Kirghiz or Kirgizstan or Laos or (Lao* adj2 Democratic adj Republic) or (Lao* adj PDR) or Latvia or Lebanon or Lesotho or Basutoland or Liberia or Libya or Lithuania or Macedonia or Madagascar or (Magalasy adj Republic) or Malawi or Malay* or Sabah or Sarawak or Maldives or Mali or (Marshall adj Island*) or Mauritania or Mauritius or (Agalega adj Island*) or Mexico or Micronesia or Moldov* or Mongolia or Montserrat or Montenegro or Morocco or Ifni or Mozambique or Myanma* or Namibia or Nauru or Nepal or (Netherlands adj Antilles) or (Dutch adj Antilles) or (New adj Guinea) or (New adj Caledonia) or Nicaragua or Niue or Niger or Nigeria or (Northern adj Mariana adj Island*) or Nyasaland or Oman or Pakistan or Palau or Panama or (Papua adj New adj Guinea) or PNG or Palestine or Paraguay or Peru or Philipines or Philippines or Phillipines or Phillippines or Poland or (Puerto adj Rico) or Yemen or Romania or Roumania or Rumania or Russia* or Rwanda or Ruanda or (Saint adj Kitts) or (St adj Kitts) or Nevis or (Saint adj Vincent) or (St adj Vincent) or Grenadines or Samoa* or (Navigator adj Island*) or (Saint adj Lucia) or (St adj Lucia) or (Saint adj Helena) or (St adj Helena) or (Sao adj Tome) or (Saudi adj Arabia) or Senegal or Serbia or Seychelles or (Sierra adj Leone) or Slovenia or Slovak* or (South adj Africa) or (Solomon adj Island*) or Somalia or (Sri adj Lanka) or Ceylon or Sudan or Surinam* or Swaziland or Syria or Tajikistan or Tadzhikistan or Tadjikistan or Tadzhik or Tanzania or Thailand or Tibet or Timor-Leste or Togo or (Togolese adj Republic) or Tokelau or Tonga or Trinidad or Tobago or Tunisia or Turkey or Turkmenistan or Turkmen or Tuvalu or Uganda or Ukraine or Uruguay or Urundi or USSR or (Soviet adj Union) or "Union of Soviet Socialist Republics" or Uzbekistan or Vanuatu or (New adj Hebrides) or Venezuela or Vietnam or (Viet adj Nam) or (Wallis adj2 Futuna) or (United adj Arab adj Republic) or (West adj Bank) or (West adj Indies) or Yemen or Yugoslavia or Zaire or Zambia or Zimbabwe or Rhodesia).tw,kf.
26. (africa or americas or caribbean or (central adj America) or (latin adj America) or (south adj America) or (eastern adj Europe) or Transcaucasia or antarctic or (atlantic adj island*) or (indian adj ocean adj island*) or

(pacific adj island*) or polynesia or (central adj asia) or (southeast* adj asia) or (south-east* adj asia) or borneo or mekong or (western adj asia) or (middle adj east) or (far adj east)).tw,kf.
27. 17 or 18 or 19 or 20 or 21 or 22 or 23 or 24 or 25 or 26
28. 5 and 10 and 15 and 16 and 27
29. (exp animals/ or (rat or rats or mouse or mice or swine or porcine or murine or sheep or lamb or lambs or pig or pigs or piglet or piglets or rabbit or rabbits or cat or cats or dog or dogs or cattle or bovine or monkey or monkeys or trout or marmoset or marmosets).ti.) not human*.sh.
30. 28 not 29
31. (((Coronavirus* or corona-virus* or nCov) and ("2019" or wuhan or china or chinese or hubei)) or COV-2 or COV2 or COVID-19 or COVID19 or COVID-2019 or COVID2019 or 2019-nCoV or nCov-2019 or Coronavirus-2 or Coronavirus2 or coronavirus-disease-2019 or corona-virus-disease-2019).tw,kf.
32. Betacoronavirus/ or Coronavirus Infections/
33. limit 30 to covid-19
34. 30 not (31 or 32 or 33)
35. limit 34 to yr="2010 -Current"
36. limit 35 to (case reports or comment or editorial or guideline or letter or practice guideline)
37. 35 not 36
38. limit 37 to english language

Text S2 – Studies excluded at full-text review

Abbas Q, Jamil MT, Jafri L, Haque AU, Khetpal V. Hyperlactetemia And Its Trends In Critically Ill Children Admitted In Pediatric Intensive Care Unit Of A Developing Country. J Ayub Med Coll Abbottabad. 2016;28:660-3.

Adhikari DD, Mahathi K, Ghosh U, Agarwal I, Chacko A, Jacob E, et al. Impact of pre-hospital care on the outcome of children arriving with agonal breathing to a pediatric emergency service in South India. Journal of Family Medicine & Primary Care. 2016;5:625-30.

Afolabi BM, Clement CO, Ekundayo A, Dolapo D. A hospital-based estimate of major causes of death among under-five children from a health facility in Lagos, Southwest Nigeria: possible indicators of health inequality. Intern. 2012;11:39.

Ahrens JO, Morrow BM, Argent AC. Influenza A(H1N1)pdm09 in critically ill children admitted to a paediatric intensive care unit, South Africa. Southern African Journal of Critical Care. 2015;31:4-7.

Al-Biltagi MA, Abo-Elezz AA, Elshafiey RM, Suliman GA, Mabrouk MM, Mourad HA. The predictive value of soluble endothelial selectin plasma levels in children with acute lung injury. J Crit Care. 2016;32:31-5.

Alam T, Ahmed T, Sarmin M, Shahrin L, Afroze F, Sharifuzzaman, et al. Risk Factors for Death in Bangladeshi Children Under 5 Years of Age Hospitalized for Diarrhea and Severe Respiratory Distress in an Urban Critical Care Ward. Glob. 2017;4:2333794X17696685.

Assane D, Makhtar C, Abdoulaye D, Amary F, Djibril B, Amadou D, et al. Viral and Bacterial Etiologies of Acute Respiratory Infections Among Children Under 5 Years in Senegal. Microbiol. 2018;11:1178636118758651.

Ayieko P, Ogero M, Makone B, Julius T, Mbevi G, Nyachiro W, et al. Characteristics of admissions and variations in the use of basic investigations, treatments and outcomes in Kenyan hospitals within a new Clinical Information Network. Arch Dis Child. 2016;101:223-9.

Ballot DE, Davies VA, Cooper PA, Chirwa T, Argent A, Mer M. Retrospective cross-sectional review of survival rates in critically ill children admitted to a combined paediatric/neonatal intensive care unit in Johannesburg, South Africa, 2013-2015. BMJ Open. 2016;6:e010850.

Banajeh SM, Ashoor O, Al-Magramy AS. Childhood very severe pneumonia and meningitis-related hospitalization and death in Yemen, before and after introduction of H. influenzae type b (Hib) vaccine. East Mediterr Health J. 2014;20:431-41.

Bari A, Sadruddin S, Khan A, Khan I, Khan A, Lehri IA, et al. Community case management of severe pneumonia with oral amoxicillin in children aged 2-59 months in Haripur district, Pakistan: a cluster randomised trial. Lancet. 2011;378:1796-803.

Barrionuevo G, Fernandez M, Larcamon J, Ortiz AM, Priano R, Fink ML. Acute lower respiratory infections and post-neonatal mortality. [Spanish]. Salud(i)Ciencia. 2015;21:262-5.

Bashir U, Nisar N, Arshad Y, Alam MM, Ashraf A, Sadia H, et al. Respiratory syncytial virus and influenza are the key viral pathogens in children <2 years hospitalized with bronchiolitis and pneumonia in Islamabad Pakistan. Arch Virol. 2017;162:763-73.

Bates M, Shibemba A, Mudenda V, Chimoga C, Tembo J, Kabwe M, et al. Burden of respiratory tract infections at post mortem in Zambian children. BMC Med. 2016;14:99.

Becerra M, Fiestas Solorzano V, Tantalean J, Mallma G, Alvarado M, Gutierrez V, et al. Viral etiology of severe acute respiratory infections in a pediatric intensive care unit. [Spanish]. Revista Peruana de Medicina Experimental y Salud Publica. 2019;36:231-8.

Benet T, Sylla M, Messaoudi M, Picot VS, Telles JN, Diakite AA, et al. Etiology and factors associated with pneumonia in children under 5 years of age in Mali: A prospective case-control study. PLoS ONE. 2015;10.

Blyth CC, Ford R, Sapura J, Kumani T, Masiria G, Kave J, et al. Childhood pneumonia and meningitis in the Eastern Highlands Province, Papua New Guinea in the era of conjugate vaccines: study methods and challenges. Pneumonia (Nathan). 2017;9:5.

Bohn JA, Kassaye BM, Record D, Chou BC, Kraft IL, Purdy JC, et al. Demographic and mortality analysis of hospitalized children at a referral hospital in Addis Ababa, Ethiopia. BMC Pediatr. 2016;16:168.

Bouyou-Akotet MK, Mawili-Mboumba DP, Kendjo E, Eyang Ekouma A, Abdou Raouf O, Engohang Allogho E, et al. Complicated malaria and other severe febrile illness in a pediatric ward in Libreville, Gabon. BMC Infect Dis. 2012;12:216.

Bucens IK, Reid A, Barreto AC, Dwivedi V, Counahan M. Three years of paediatric morbidity and mortality at the National Hospital in Dili, East Timor. J Paediatr Child Health. 2013;49:1004-9.

Caballero MT, Bianchi AM, Nuno A, Ferretti AJP, Polack LM, Remondino I, et al. Mortality Associated With Acute Respiratory Infections Among Children at Home. J Infect Dis. 2019;219:358-64.

Cai XY, Wang Q, Lin GY, Cai ZW, Lin CX, Chen PZ, et al. Respiratory virus infections among children in South China. J Med Virol. 2014;86:1249-55.

Caini S, de Mora D, Olmedo M, Portugal D, Becerra MA, Mejia M, et al. The epidemiology and severity of respiratory viral infections in a tropical country: Ecuador, 2009-2016. J Infect Public Health. 2019;12:357-63.

Carugati M, Zhang HL, Kilonzo KG, Maze MJ, Maro VP, Rubach MP, et al. Predicting Mortality for Adolescent and Adult Patients with Fever in Resource-Limited Settings. Am J Trop Med Hyg. 2018;99:1246-54.

Charles NC, Chuku A, Anazodo NM. Childhood mortality in federal medical centre umuahia, South eastern Nigeria. Oman med. 2014;29:320-4.

Chelwa NM, Likwa RN, Banda J. Under-five mortality among displaced populations in Meheba refugee camp, Zambia, 2008-2014. Archives of Public Health. 2016;74:49.

Chheng K, Carter MJ, Emary K, Chanpheaktra N, Moore CE, Stoesser N, et al. A prospective study of the causes of febrile illness requiring hospitalization in children in Cambodia. PLoS ONE. 2013;8:e60634.

Chisti MJ, Ahmed T, Ashraf H, Faruque AS, Bardhan PK, Dey SK, et al. Clinical predictors and outcome of metabolic acidosis in under-five children admitted to an urban hospital in Bangladesh with diarrhea and pneumonia. PLoS ONE. 2012;7:e39164.

Chisti MJ, Duke T, Robertson CF, Ahmed T, Faruque AS, Ashraf H, et al. Clinical predictors and outcome of hypoxaemia among under-five diarrhoeal children with or without pneumonia in an urban hospital, Dhaka, Bangladesh. Trop Med Int Health. 2012;17:106-11.

Chisti MJ, Duke T, Robertson CF, Ahmed T, Faruque AS, Bardhan PK, et al. Co-morbidity: exploring the clinical overlap between pneumonia and diarrhoea in a hospital in Dhaka, Bangladesh. Ann Trop Paediatr. 2011;31:311-9.

Chisti MJ, Graham SM, Duke T, Ahmed T, Ashraf H, Faruque AS, et al. A prospective study of the prevalence of tuberculosis and bacteraemia in Bangladeshi children with severe malnutrition and pneumonia including an evaluation of Xpert MTB/RIF assay. PLoS ONE. 2014;9:e93776.

Chisti MJ, Pietroni MA, Smith JH, Bardhan PK, Salam MA. Predictors of death in under-five children with diarrhoea admitted to a critical care ward in an urban hospital in Bangladesh. Acta Paediatr. 2011;100:e275-9.

Chisti MJ, Salam MA, Ahmed T, Shahid AS, Shahunja KM, Faruque AS, et al. Lack of BCG vaccination and other risk factors for bacteraemia in severely malnourished children with pneumonia. Epidemiol Infect. 2015;143:799-803.

Chisti MJ, Salam MA, Ashraf H, Faruque AS, Bardhan PK, Das SK, et al. Clinical signs of radiologic pneumonia in under-five hypokalemic diarrheal children admitted to an urban hospital in bangladesh. PLoS ONE. 2013;8:e71911.

Chisti MJ, Salam MA, Ashraf H, Faruque AS, Bardhan PK, Hossain MI, et al. Clinical risk factors of death from pneumonia in children with severe acute malnutrition in an urban critical care ward of Bangladesh. PLoS ONE. 2013;8:e73728.

Chisti MJ, Salam MA, Ashraf H, Faruque AS, Bardhan PK, Shahid AS, et al. Predictors and outcome of hypoxemia in severely malnourished children under five with pneumonia: a case control design. PLoS ONE. 2013;8:e51376.

Chisti MJ, Salam MA, Bardhan PK, Faruque AS, Shahid AS, Shahunja KM, et al. Severe Sepsis in Severely Malnourished Young Bangladeshi Children with Pneumonia: A Retrospective Case Control Study. PLoS ONE. 2015;10:e0139966.

Chisti MJ, Salam MA, Bardhan PK, Faruque AS, Shahid AS, Shahunja KM, et al. Treatment Failure and Mortality amongst Children with Severe Acute Malnutrition Presenting with Cough or Respiratory Difficulty and Radiological Pneumonia. PLoS ONE. 2015;10:e0140327.

Chisti MJ, Salam MA, Smith JH, Ahmed T, Pietroni MA, Shahunja KM, et al. Bubble continuous positive airway pressure for children with severe pneumonia and hypoxaemia in Bangladesh: an open, randomised controlled trial. Lancet. 2015;386:1057-65.

Cohen C, Moyes J, Tempia S, Groome M, Walaza S, Pretorius M, et al. Epidemiology of Acute Lower Respiratory Tract Infection in HIV-Exposed Uninfected Infants. Pediatrics. 2016;137.

Cohen C, Walaza S, Moyes J, Groome M, Tempia S, Pretorius M, et al. Epidemiology of severe acute respiratory illness (SARI) among adults and children aged >=5 years in a high HIV-prevalence setting, 2009-2012. PLoS ONE. 2015;10:e0117716.

Cummings MJ, Bakamutumaho B, Kayiwa J, Byaruhanga T, Owor N, Namagambo B, et al. Epidemiologic and Spatiotemporal Characterization of Influenza and Severe Acute Respiratory Infection in Uganda, 2010-2015. Ann Am Thorac Soc. 2016;13:2159-68.

Dainguy M, Folquet A, Akaffou E, Sylla M, Kouadio E, Kouakou C, et al. Ambulatory Monitoring of Preterm Infant During Their First Year of Life at the Pediatric Deparment of Cocody Teaching Hospital. Mali med. 2011;26:25-9.

Dass R, Deka NM, Barman H, Duwarah SG, Khyriem AB, Saikia MK, et al. Empyema thoracis: analysis of 150 cases from a tertiary care centre in North East India. Indian J Pediatr. 2011;78:1371-7.

Deribew A, Tessema GA, Deribe K, Melaku YA, Lakew Y, Amare AT, et al. Trends, causes, and risk factors of mortality among children under 5 in Ethiopia, 1990-2013: findings from the Global Burden of Disease Study 2013. Popul Health Metr. 2016;14:42.

Derrar F, Izri K, Kaddache C, Boukari R, Hannoun D. Virologic study of acute lower respiratory tract infections in children admitted to the paediatric department of Blida University Hospital, Algeria. New microbes new infect. 2019;30:100536.

Duyu M, Karakaya Z. Viral Etiology and Outcome of Severe Lower Respiratory Tract Infections among Critically Ill Children Admitted to the Picu. Med. 2020;13:13.

El Basha NR, Marzouk H, Sherif MM, El Kholy AA. Prematurity, a significant predictor for worse outcome in viral bronchiolitis: a comparative study in infancy. J Egypt Public Health Assoc. 2019;94:15.

El Kholy AA, Mostafa NA, Ali AA, El-Sherbini SA, Ismail RI, Magdy RI, et al. Risk factors of prolonged hospital stay in children with viral severe acute respiratory infections. Journal of Infection in Developing Countries. 2014;8:1285-93.

El Sakka AS, Imam SS, Amer HA, Moustafa SA. Vitamin D deficiency and low hemoglobin level as risk factors for severity of acute lower respiratory tract infections in Egyptian children: A case-control study. Egyptian Pediatric Association Gazette. 2014;62:1-7.

Fallahzadeh MA, Abdehou ST, Hassanzadeh J, Fallhzadeh F, Fallahzadeh MH, Malekmakan L. Pattern of in-hospital pediatric mortality over a 3-year period at University teaching hospitals in Iran. Indian Journal of Critical Care Medicine. 2015;19:311-5.

Faneye A, Motayo BO, Adesanmi A, Onoja B. Evaluation of IgG Antibodies Against Respiratory Syncytial Virus (RSV), and Associated Risk Factors for Severe Respiratory Tract Infections in Pre-School Children in North-Central, Nigeria. African Journal of Infectious Diseases. 2014;8:36-9.

Ferdous F, Ahmed S, Das SK, Chisti MJ, Nasrin D, Kotloff KL, et al. Pneumonia mortality and healthcare utilization in young children in rural Bangladesh: a prospective verbal autopsy study. Trop. 2018;46:17.

Fernandez AL, Arias Lopez MP, Ratto ME, Saligari L, Siaba Serrate A, de la Rosa M, et al. Validation of the Pediatric Index of Mortality 2 (PIM2) in Argentina: a prospective, multicenter, observational study. Arch Argent Pediatr. 2015;113:221-8.

Ferrand RA, Bandason T, Musvaire P, Larke N, Nathoo K, Mujuru H, et al. Causes of acute hospitalization in adolescence: burden and spectrum of HIV-related morbidity in a country with an early-onset and severe HIV epidemic: a prospective survey. PLoS Med. 2010;7:e1000178.

Ferreira H, Costa KLP, Cariolano MS, Oliveira GS, Felipe KKP, Silva ESA, et al. High incidence of rhinovirus infection in children with community-acquired pneumonia from a city in the Brazilian pre-Amazon region. J Med Virol. 2019;91:1751-8.

Ferrero F. Lactate as a predictor of mortality in Malawian children with WHO-defined pneumonia. [Spanish]. Arch Argent Pediatr. 2012;110:449.

Fieldhouse JK, Toh TH, Lim WH, Ting J, Ha SJ, Hii KC, et al. Surveillance for respiratory syncytial virus and parainfluenza virus among patients hospitalized with pneumonia in Sarawak, Malaysia. PLoS ONE. 2018;13:e0202147.

Fischer Walker CL, Perin J, Liu JL, Katz J, Tielsch JM, Black R. Does comorbidity increase the risk of mortality among children under 3 years of age? BMJ Open. 2013;3.

Gathara D, Irimu G, Kihara H, Maina C, Mbori-Ngacha D, Mwangi J, et al. Hospital outcomes for paediatric pneumonia and diarrhoea patients admitted in a tertiary hospital on weekdays versus weekends: a retrospective study. BMC Pediatr. 2013;13:74.

Gladstone BP, Das AR, Rehman AM, Jaffar S, Estes MK, Muliyil J, et al. Burden of illness in the first 3 years of life in an Indian slum. J Trop Pediatr. 2010;56:221-6.

Gordon DM, Frenning S, Draper HR, Kokeb M. Prevalence and burden of diseases presenting to a general pediatrics ward in Gondar, Ethiopia. J Trop Pediatr. 2013;59:350-7.

Graham H, Bakare AA, Ayede AI, Oyewole OB, Gray A, Neal E, et al. Diagnosis of pneumonia and malaria in Nigerian hospitals: A prospective cohort study. Pediatr Pulmonol. 2020;55 Suppl 1:S37-S50.

Graham HR, Bakare AA, Ayede AI, Gray AZ, McPake B, Peel D, et al. Oxygen systems to improve clinical care and outcomes for children and neonates: A stepped-wedge cluster-randomised trial in Nigeria. PLoS Med. 2019;16:e1002951.

Grassi T, Mancini F, Ciervo A, Vescio MF, Ghazal A, Ashour H, et al. Chlamydophila pneumoniae, Mycoplasma pneumoniae, and influenza in children with respiratory infections in Alexandria, Egypt. Journal of Infection in Developing Countries. 2014;8:379-83.

Gray AZ, Morpeth M, Duke T, Peel D, Winter C, Satvady M, et al. Improved oxygen systems in district hospitals in Lao PDR: a prospective field trial of the impact on outcomes for childhood pneumonia and equipment sustainability. BMJ paediatr. 2017;1:e000083.

Gupta N, Hirschhorn LR, Rwabukwisi FC, Drobac P, Sayinzoga F, Mugeni C, et al. Causes of death and predictors of childhood mortality in Rwanda: a matched case-control study using verbal social autopsy. BMC Public Health. 2018;18:1378.

Hammitt LL, Kazungu S, Morpeth SC, Gibson DG, Mvera B, Brent AJ, et al. A preliminary study of pneumonia etiology among hospitalized children in Kenya. Clin Infect Dis. 2012;54 Suppl 2:S190-9.

Hariharan S, Krishnamurthy K, Grannum D. Validation of Pediatric Index of Mortality-2 scoring system in a pediatric intensive care unit, Barbados. J Trop Pediatr. 2011;57:9-13.

Harris C, Mills R, Seager E, Blackstock S, Hiwa T, Pumphrey J, et al. Paediatric deaths in a tertiary government hospital setting, Malawi. Paediatr Int Child Health. 2019;39:240-8.

Hooli S, King C, Zadutsa B, Nambiar B, Makwenda C, Masache G, et al. The epidemiology of hypoxemic pneumonia among young infants in Malawi. American Journal of Tropical Medicine and Hygiene. 2020;102:676-83.

Horton KC, Dueger EL, Kandeel A, Abdallat M, El-Kholy A, Al-Awaidy S, et al. Viral etiology, seasonality and severity of hospitalized patients with severe acute respiratory infections in the Eastern Mediterranean Region, 2007-2014. PLoS ONE. 2017;12:e0180954.

Hu X, Qian S, Xu F, Huang B, Zhou D, Wang Y, et al. Incidence, management and mortality of acute hypoxemic respiratory failure and acute respiratory distress syndrome from a prospective study of Chinese paediatric intensive care network. Acta Paediatr. 2010;99:715-21.

Jofiro G, Jemal K, Beza L, Bacha Heye T. Prevalence and associated factors of pediatric emergency mortality at Tikur Anbessa specialized tertiary hospital: a 5 year retrospective case review study. BMC Pediatr. 2018;18:316.

Kahabuka C, Kvale G, Hinderaker SG. Factors associated with severe disease from malaria, pneumonia and diarrhea among children in rural Tanzania - A hospital-based cross-sectional study. BMC Infect Dis. 2012;12 (no pagination).

Kasundriya SK, Dhaneria M, Mathur A, Pathak A. Incidence and Risk Factors for Severe Pneumonia in Children Hospitalized with Pneumonia in Ujjain, India. Int J Environ Res Public Health. 2020;17:27.

Keitel K, Kagoro F, Samaka J, Masimba J, Said Z, Temba H, et al. A novel electronic algorithm using host biomarker point-of-care tests for the management of febrile illnesses in Tanzanian children (e-POCT): A randomized, controlled non-inferiority trial. PLoS Med. 2017;14:e1002411.

Keitel K, Samaka J, Masimba J, Temba H, Said Z, Kagoro F, et al. Safety and Efficacy of C-reactive Protein-guided Antibiotic Use to Treat Acute Respiratory Infections in Tanzanian Children: A Planned Subgroup Analysis of a Randomized Controlled Noninferiority Trial Evaluating a Novel Electronic Clinical Decision Algorithm (ePOCT). Clin Infect Dis. 2019;69:1926-34.

Khuri-Bulos N, Lawrence L, Piya B, Wang L, Fonnesbeck C, Faouri S, et al. Severe outcomes associated with respiratory viruses in newborns and infants: a prospective viral surveillance study in Jordan. BMJ Open. 2018;8:e021898.

Kisenge RR, Rees CA, Lauer JM, Liu E, Fawzi WW, Manji KP, et al. Risk factors for mortality among infants and children. Trop. 2020;48:43.

Kitchin OP, Masekela R, Becker P, Moodley T, Risenga SM, Green RJ. Outcome of human immunodeficiency virus-exposed and -infected children admitted to a pediatric intensive care unit for respiratory failure. Pediatr Crit Care Med. 2012;13:516-9.

Kockuzu E, Bayrakci B, Kesici S, Citak A, Karapinar B, Emeksiz S, et al. Comprehensive Analysis of Severe Viral Infections of Respiratory Tract admitted to PICUs during the Winter Season in Turkey. Indian Journal of Critical Care Medicine. 2019;23:263-9.

Kruger I, Gie R, Kruger M, Harvey J. Outcome of children admitted to a general high-care unit in a regional hospital in the Western Cape, South Africa. SAJCH South African Journal of Child Health. 2016;10:156-60.

Kuti BP, Adegoke SA, Ebruke BE, Howie S, Oyelami OA, Ota M. Determinants of oxygen therapy in childhood pneumonia in a resource-constrained region. ISRN pediatr. 2013;2013:435976.

Kuti BP, Adegoke SA, Oyelami OA, Ota MO. Predictors of prolonged hospitalisation in childhood pneumonia in a rural health centre. SAJCH South African Journal of Child Health. 2014;8:11-5.

Lamarao LM, Ramos FL, Mello WA, Santos MC, Barbagelata LS, Justino MC, et al. Prevalence and clinical features of respiratory syncytial virus in children hospitalized for community-acquired pneumonia in northern Brazil. BMC Infect Dis. 2012;12:119.

Le Geyt J, Hauck S, Lee M, Mackintosh J, Slater J, Razon D, et al. Respiratory syncytial virus prevalence in children admitted to five Kenyan district hospitals: a cross-sectional study. BMJ paediatr. 2019;3:e000409.

le Roux DM, Nicol MP, Myer L, Vanker A, Stadler JAM, von Delft E, et al. Lower Respiratory Tract Infections in Children in a Well-vaccinated South African Birth Cohort: Spectrum of Disease and Risk Factors. Clin Infect Dis. 2019;69:1588-96.

Leotte J, Trombetta H, Faggion HZ, Almeida BM, Nogueira MB, Vidal LR, et al. Impact and seasonality of human rhinovirus infection in hospitalized patients for two consecutive years. J Pediatr (Rio J). 2017;93:294-300.

Leung DT, Das SK, Malek MA, Qadri F, Faruque AS, Chisti MJ, et al. Concurrent Pneumonia in Children Under 5 Years of Age Presenting to a Diarrheal Hospital in Dhaka, Bangladesh. Am J Trop Med Hyg. 2015;93:831-5.

Li Y, Wang Q, Chen H, Gao HM, Zhou T, Qian SY. Epidemiological features and risk factor analysis of children with acute lung injury. World J Pediatr. 2012;8:43-6.

Lonngren C, Morrow BM, Haynes S, Yusri T, Vyas H, Argent AC. North-South divide: distribution and outcome of respiratory viral infections in paediatric intensive care units in Cape Town (South Africa) and Nottingham (United Kingdom). J Paediatr Child Health. 2014;50:208-15.

Lowlaavar N, Larson CP, Kumbakumba E, Zhou G, Ansermino JM, Singer J, et al. Pediatric in-Hospital Death from Infectious Disease in Uganda: Derivation of Clinical Prediction Models. PLoS ONE. 2016;11:e0150683.

Malekshahi SS, Azad TM, Yavarian J, Shahmahmoodi S, Naseri M, Rezaei F. Molecular detection of respiratory viruses in clinical specimens from children with acute respiratory disease in Iran. Pediatr Infect Dis J. 2010;29:931-3.

Malla L, Perera-Salazar R, McFadden E, English M. Comparative effectiveness of injectable penicillin versus a combination of penicillin and gentamicin in children with pneumonia characterised by indrawing in Kenya: a retrospective observational study. BMJ Open. 2017;7:e019478.

Mathisen M, Strand TA, Sharma BN, Chandyo RK, Valentiner-Branth P, Basnet S, et al. Clinical presentation and severity of viral community-acquired pneumonia in young Nepalese children. Pediatr Infect Dis J. 2010;29:e1-6.

Matute SED, Donadi EA, Nunes AA, Martinez EZ. Clinical response to antibiotics in indigenous versus non-indigenous children under 5 years old with community-acquired pneumonia in Otavalo, Ecuador. Rev Soc Bras Med Trop. 2020;53:e20200038.

Mdala JF, Mash R. Causes of mortality and associated modifiable health care factors for children (< 5-years) admitted at Onandjokwe Hospital, Namibia. African Journal of Primary Health Care & Family Medicine. 2015;7:03.

Meerhoff TJ, Simaku A, Ulqinaku D, Torosyan L, Gribkova N, Shimanovich V, et al. Surveillance for severe acute respiratory infections (SARI) in hospitals in the WHO European region - an exploratory analysis of risk factors for a severe outcome in influenza-positive SARI cases. BMC Infect Dis. 2015;15:1.

Moreno-Valencia Y, Hernandez-Hernandez VA, Romero-Espinoza JAI, Coronel-Tellez RH, Castillejos-Lopez M, Hernandez A, et al. Detection and characterization of respiratory viruses causing acute respiratory illness and asthma exacerbation in children during three different seasons (2011-2014) in Mexico City. Influenza other respi. 2015;9:287-92.

Morrow BM, Hsaio NY, Zampoli M, Whitelaw A, Zar HJ. Pneumocystis pneumonia in South African children with and without human immunodeficiency virus infection in the era of highly active antiretroviral therapy. Pediatr Infect Dis J. 2010;29:535-9.

Muhanuzi B, Sawe HR, Kilindimo SS, Mfinanga JA, Weber EJ. Respiratory compromise in children presenting to an urban emergency department of a tertiary hospital in Tanzania: a descriptive cohort study. BMC emerg. 2019;19:21.

Mujuru HA, Kambarami RA. Mortality within 24 hours of admission to the Paediatric Unit, Harare Central Hospital, Zimbabwe. Cent Afr J Med. 2012;58:17-22.

Mukhtar B, Siddiqui NR, Haque A. Clinical Characteristics and Immediate-Outcome of Children Mechanically Ventilated in PICU of Pakistan. Pakistan Journal of Medical Sciences. 2014;30:927-30.

Naja Z, Fayad D, Khafaja S, Chamseddine S, Dbaibo G, Hanna-Wakim R. Bronchiolitis Admissions in a Lebanese Tertiary Medical Center: A 10 Years' Experience. Frontiers in Pediatrics. 2019;7:189.

Nguyen TKP, Nguyen DV, Truong TNH, Tran MD, Graham SM, Marais BJ. Disease spectrum and management of children admitted with acute respiratory infection in Viet Nam. Trop Med Int Health. 2017;22:688-95.

Njuguna HN, Chaves SS, Emukule GO, Nyawanda B, Omballa V, Juma B, et al. The contribution of respiratory pathogens to fatal and non-fatal respiratory hospitalizations: a pilot study of Taqman Array Cards (TAC) in Kenya. BMC Infect Dis. 2017;17:591.

O'Brien KL, Baggett HC, Brooks WA, Feikin DR, Hammitt LL, Higdon MM, et al. Causes of severe pneumonia requiring hospital admission in children without HIV infection from Africa and Asia: the PERCH multi-country case-control study. The Lancet. 2019;394:757-79.

Onyango D, Kikuvi G, Amukoye E, Omolo J. Risk factors of severe pneumonia among children aged 2-59 months in western Kenya: a case control study. Pan Afr Med J. 2012;13:45.

Patel AB, Bang A, Singh M, Dhande L, Chelliah LR, Malik A, et al. A randomized controlled trial of hospital versus home based therapy with oral amoxicillin for severe pneumonia in children aged 3 - 59 months: The IndiaCLEN Severe Pneumonia Oral Therapy (ISPOT) Study. BMC Pediatr. 2015;15.

Peng Z, Xu J, Yu Z, Sun Q, Li L, Yang P, et al. Clinical characteristics of hospitalized severe acute respiratory illnesses (SARI) in children and risk factors analysis of severe illness: results from SARI patients under 15-year-old of sentinel surveillance in 10 cities, China. [Chinese]. Zhonghua yu fang yi xue za zhi [Chinese journal of preventive medicine]. 2015;49:534-40.

Pham HT, Nguyen PTT, Tran ST, Phung TTB. Clinical and Pathogenic Characteristics of Lower Respiratory Tract Infection Treated at the Vietnam National Children's Hospital. Can. 2020;2020:7931950.

Pratheepamornkull T, Ratanakorn W, Samransamruajkit R, Poovorawan Y. Causative Agents of Severe Community Acquired Viral Pneumonia among Children in Eastern Thailand. The Southeast Asian journal of tropical medicine and public health. 2015;46:650-6.

PrayGod G, Mukerebe C, Magawa R, Jeremiah K, Torok ME. Indoor Air Pollution and Delayed Measles Vaccination Increase the Risk of Severe Pneumonia in Children: Results from a Case-Control Study in Mwanza, Tanzania. PLoS ONE. 2016;11:e0160804.

Rodriguez-Martinez CE, Acuna-Cordero R, Sossa-Briceno MP. Predictors of prolonged length of hospital stay or readmissions for acute viral lower respiratory tract infections among infants with a history of bronchopulmonary dysplasia. J Med Virol. 2018;90:405-11.

Rudan I, O'Brien KL, Nair H, Liu L, Theodoratou E, Qazi S, et al. Epidemiology and etiology of childhood pneumonia in 2010: estimates of incidence, severe morbidity, mortality, underlying risk factors and causative pathogens for 192 countries. J Glob Health. 2013;3:010401.

Saha H, Shahrin L, Sarmin M, Ahmed T, Chisti MJ. Bacteremia in Diarrheal Children With Severe Pneumonia. Glob. 2019;6:2333794X19862462.

Salih KE, Salih A, El Samani el FZ, Hussien KE, Ibrahim SA. Characteristics associated with severe pneumonia in under-five children admitted to emergency units of two teaching hospitals in Khartoum, Sudan. Sudan. 2011;11:25-31.

Salih KM, Bilal JA, Eldouch W, Abdin A. Assessment of Treatment of Community Acquired Severe Pneumonia by Two Different Antibiotics. J Clin Diagn Res. 2016;10:SC06-9.

Sentilhes AC, Choumlivong K, Celhay O, Sisouk T, Phonekeo D, Vongphrachanh P, et al. Respiratory virus infections in hospitalized children and adults in Lao PDR. Influenza other respi. 2013;7:1070-8.

Soofi S, Ahmed S, Fox MP, MacLeod WB, Thea DM, Qazi SA, et al. Effectiveness of community case management of severe pneumonia with oral amoxicillin in children aged 2-59 months in Matiari district, rural Pakistan: a cluster-randomised controlled trial. Lancet. 2012;379:729-37.

Teeratakulpisarn J, Uppala R, Thepsuthammarat K, Sutra S. Burden of acute lower respiratory infection in children in Thailand in 2010: have we achieved the national target in under-five morbidity and mortality? J Med Assoc Thai. 2012;95 Suppl 7:S87-96.

Thabet AA, Al-Kohani A, Shadoul A, Al-Mahaqri A, Bin Yahya M, Saleh AH, et al. Characteristics of severe acute respiratory infectionassociated hospitalization in Yemen, 2014/15. East Mediterr Health J. 2016;22:440-4.

Thomas DS, Anthamatten P, Root ED, Lucero M, Nohynek H, Tallo V, et al. Disease mapping for informing targeted health interventions: childhood pneumonia in Bohol, Philippines. Trop Med Int Health. 2015;20:1525-33.

Tran DN, Pham TM, Ha MT, Tran TT, Dang TK, Yoshida LM, et al. Molecular epidemiology and disease severity of human respiratory syncytial virus in Vietnam. PLoS ONE. 2013;8:e45436.

Turnbull H, Conroy A, Opoka RO, Namasopo S, Kain KC, Hawkes M. Solar-powered oxygen delivery: proof of concept. Int J Tuberc Lung Dis. 2016;20:696-703.

Turner P, Turner C, Watthanaworawit W, Carrara V, Cicelia N, Deglise C, et al. Respiratory virus surveillance in hospitalised pneumonia patients on the Thailand-Myanmar border. BMC Infect Dis. 2013;13:434.

Valentiner-Branth P, Shrestha PS, Chandyo RK, Mathisen M, Basnet S, Bhandari N, et al. A randomized controlled trial of the effect of zinc as adjuvant therapy in children 2-35 mo of age with severe or nonsevere pneumonia in Bhaktapur, Nepal. Am J Clin Nutr. 2010;91:1667-74.

Van de Steen O, Miri F, Gunjaca M, Klepac V, Gross B, Notario G, et al. The Burden of Severe Respiratory Syncytial Virus Disease Among Children Younger than 1 Year in Central and Eastern Europe. Infectious Diseases & Therapy. 2016;5:125-37.

Vasconcellos Â G, Clarêncio J, Andrade D, Cardoso MA, Barral A, Nascimento-Carvalho CM. Systemic cytokines and chemokines on admission of children hospitalized with community-acquired pneumonia. Cytokine. 2018;107:1-8.

Vilas-Boas AL, Fontoura MS, Xavier-Souza G, Araujo-Neto CA, Andrade SC, Brim RV, et al. Comparison of oral amoxicillin given thrice or twice daily to children between 2 and 59 months old with non-severe pneumonia: a randomized controlled trial. J Antimicrob Chemother. 2014;69:1954-9.

von Mollendorf C, La Vincente S, Ulziibayar M, Suuri B, Luvsantseren D, Narangerel D, et al. Epidemiology of pneumonia in the pre-pneumococcal conjugate vaccine era in children 2-59 months of age, in Ulaanbaatar, Mongolia, 2015-2016. PLoS ONE. 2019;14:e0222423.

Wadhwa N, Chandran A, Aneja S, Lodha R, Kabra SK, Chaturvedi MK, et al. Efficacy of zinc given as an adjunct in the treatment of severe and very severe pneumonia in hospitalized children 2-24 mo of age: a randomized, double-blind, placebo-controlled trial. Am J Clin Nutr. 2013;97:1387-94.

Weinberg A, Mussi-Pinhata MM, Yu Q, Cohen RA, Almeida VC, Amaral FR, et al. Factors Associated with Lower Respiratory Tract Infections in HIV-Exposed Uninfected Infants. AIDS Res Hum Retroviruses. 2018;34:527-35.

Wen Z, Wei J, Xue H, Chen Y, Melnick D, Gonzalez J, et al. Epidemiology, microbiology, and treatment patterns of pediatric patients hospitalized with pneumonia at two hospitals in China: a patient chart review study. Ther Clin Risk Manag. 2018;14:501-10.

Zar HJ, Barnett W, Stadler A, Gardner-Lubbe S, Myer L, Nicol MP. Aetiology of childhood pneumonia in a well vaccinated South African birth cohort: a nested case-control study of the Drakenstein Child Health Study. Lancet Respir Med. 2016;4:463-72.

Table S1 – EPHPP quality assessment of included studies

Author & Year	Selection bias	Study design	Confounders	Blinding	Data collection methods	Withdrawal and drop-outs	Analyses	OVERALL
Abdulkadir 2015	Mod	Mod	Mod	N/A	Mod	Strong	Mod	Mod
Acuna 2018	Can't tell	Mod	Can't tell	N/A	Can't tell	Can't tell	Can't tell	Weak
Adewuyi 2012	Can't tell	Mod	Can't tell	N/A	Can't tell	Can't tell	Can't tell	Weak
Agweyu 2015	Strong	Strong	Strong	Weak	Weak	Strong	Strong	Mod
Agweyu 2018	Strong	Mod	Strong	N/A	Weak	Strong	Strong	Mod
Agweyu 2018	Strong	Mod	Strong	N/A	Weak	Strong	Strong	Mod
Ahmed 2016	Strong	Mod	Mod	N/A	Mod	Strong	Mod	Mod
Ahmed 2018	Mod	Mod	Weak	N/A	Weak	Strong	Mod	Weak
Al Amad 2019	Mod	Mod	Weak	N/A	Weak	Strong	Mod	Weak
Ali 2013	Weak	Mod	Weak	N/A	Weak	Strong	Mod	Weak
Alohan 2019	Can't tell	Mod	Can't tell	N/A	Can't tell	Can't tell	Can't tell	Weak
Araya 2016	Mod	Mod	Mod	N/A	Mod	Strong	Strong	Mod
Atwa 2015	Mod	Mod	Mod	N/A	Mod	Strong	Strong	Mod
Awad 2020	Mod	Weak	Weak	N/A	Weak	Strong	Mod	Weak
Awasthi 2018	Strong	Mod	Mod	N/A	Mod	Strong	Mod	Mod
Ayieko 2012	Mod	Mod	Mod	N/A	Mod	Strong	Strong	Mod
Azab 2016	Mod	Mod	Mod	N/A	Weak	Strong	Strong	Mod
Azab 2014	Strong	Weak	Mod	N/A	Weak	Strong	Strong	Mod
Barger-Kamate 2016	Strong	Mod	Strong	N/A	Mod	Strong	Strong	Mod
Basnet 2015	Strong	Mod	Strong	N/A	Weak	Strong	Strong	Mod
Bekele 2017	Mod	Mod	Weak	N/A	Mod	Mod	Mod	Mod
Benet 2017	Strong	Mod	Strong	N/A	Mod	Strong	Strong	Mod
Berkley 2010	Mod	Mod	Mod	N/A	Mod	Mod	Mod	Mod
Bezerra 2011	Weak	Mod	Weak	N/A	Weak	Strong	Mod	Weak
Bills 2020	Mod	Mod	Strong	N/A	Mod	Strong	Strong	Mod
Bjorklund 2019	Strong	Mod	Strong	Weak	Weak	Strong	Strong	Mod
Bokade 2015	Mod	Mod	Mod	N/A	Mod	Mod	Mod	Mod
Boukari 2011	Can't tell	Mod	Can't tell	N/A	Can't tell	Can't tell	Can't tell	Weak
Caggiano 2017	Mod	Mod	Mod	N/A	Mod	Mod	Mod	Mod
Champatiray 2017	Mod	Mod	Mod	N/A	Mod	Mod	Mod	Mod
Chisti 2010	Mod	Mod	Mod	N/A	Weak	Mod	Strong	Mod
Chowdury 2020	Strong	Mod	Mod	N/A	Weak	Strong	Strong	Mod
Cohen 2015	Mod	Mod	Mod	N/A	Mod	Mod	Strong	Mod
Cotes 2015	Weak	Mod	Mod	N/A	Mod	Strong	Mod	Mod
Daga 2014	Can't tell	Mod	Can't tell	N/A	Can't tell	Can't tell	Can't tell	Weak
Dembele 2019	Strong	Mod	Strong	N/A	Mod	Strong	Strong	Mod
Diez-Padrisa 2010	Weak	Mod	Mod	N/A	Mod	Mod	Strong	Mod

Divecha 2019	Mod	Mod	Strong	N/A	Mod	Strong	Strong	Mod
Do 2011	Strong	Mod	Strong	N/A	Mod	Strong	Strong	Mod
Durigon 2015	Strong	Mod	Strong	N/A	Mod	Strong	Mod	Mod
Emukule 2014	Strong	Mod	Strong	N/A	Mod	Strong	Strong	Mod
Enarson 2015	Strong	Mod	Strong	N/A	Mod	Strong	Strong	Mod
Enarson 2014	Strong	Mod	Strong	N/A	Mod	Strong	Strong	Mod
Evelyn 2019	Mod	Mod	Mod	N/A	Mod	Mod	Mod	Mod
Ezeonu 2015	Weak	Mod	Weak	N/A	Weak	Weak	Mod	Weak
Fagbohun 2020	Can’t tell	Mod	Can’t tell	N/A	Can’t tell	Can’t tell	Can’t tell	Weak
Fancourt 2017	Strong	Mod	Strong	N/A	Mod	Strong	Strong	Mod
Feikin 2017	Strong	Mod	Strong	N/A	Mod	Strong	Strong	Mod
Ferolla 2013	Mod	Mod	Strong	N/A	Mod	Strong	Strong	Mod
Ferreira 2014	Strong	Mod	Mod	N/A	Mod	Strong	Strong	Mod
Fischer Langley 2013	Mod	Mod	Strong	N/A	Mod	Strong	Strong	Mod
Gallagher 2020	Strong	Mod	Strong	N/A	Mod	Strong	Strong	Mod
Gowraiah 2014	Strong	Mod	Strong	N/A	Mod	Strong	Mod	Mod
Graham 2019	Strong	Mod	Strong	N/A	Mod	Strong	Strong	Mod
Graham 2011	Strong	Mod	Mod	N/A	Mod	Strong	Strong	Mod
Hasan 2014	Weak	Mod	Mod	N/A	Mod	Strong	Mod	Mod
Hatem 2019	Weak	Weak	Weak	N/A	Mod	Strong	Mod	Weak
Hooli 2016	Mod	Mod	Mod	N/A	Mod	Mod	Strong	Mod
Hutton 2019	Mod	Mod	Mod	N/A	Mod	Strong	Strong	Mod
Ibraheem 2020	Weak	Mod	Weak	N/A	Mod	Strong	Mod	Mod
Indriyani 2018	Can’t tell	Mod	Can’t tell	N/A	Can’t tell	Can’t tell	Can’t tell	Weak
Indriyani 2019	Can’t tell	Mod	Can’t tell	N/A	Can’t tell	Can’t tell	Can’t tell	Weak
Iroh Tam 2018	Mod	Mod	Mod	N/A	Mod	Strong	Mod	Mod
Jain 2018	Mod	Mod	Mod	N/A	Mod	Strong	Strong	Mod
Jakhar 2018	Mod	Mod	Mod	N/A	Mod	Mod	Mod	Mod
Jroundi 2014	Mod	Mod	Mod	N/A	Mod	Mod	Mod	Mod
Jroundi 2014	Mod	Mod	Mod	N/A	Mod	Mod	Mod	Mod
Julien 2020	Weak	Mod	Mod	N/A	Mod	Strong	Mod	Weak
Kelly 2015	Mod	Mod	Strong	Strong	Mod	Strong	Strong	Mod
Kelly 2015	Mod	Mod	Strong	Strong	Mod	Strong	Strong	Mod
Kelly 2019	Mod	Mod	Strong	Strong	Mod	Strong	Strong	Mod
Kenmoe 2019	Strong	Strong	Strong	N/A	Strong	Strong	Strong	Strong
Khuri-Bulos 2020	Weak	Mod	Can’t tell	N/A	Can’t tell	Can’t tell	Can’t tell	Weak
Kim 2019	Mod	Mod	Mod	N/A	Can’t tell	Can’t tell	Can’t tell	Weak
King 2015	Mod	Mod	Mod	N/A	Mod	Mod	Mod	Mod
Korkmaz 2018	Mod	Mod	Weak	N/A	Mod	Can’t tell	Mod	Mod
Ku 2020	Strong	Weak	Weak	N/A	Weak	Can’t tell	Mod	Weak
Kuti 2013	Strong	Mod	Mod	N/A	Mod	Strong	Mod	Mod
Laman 2013	can't tell	can't tell	can't tell	N/A	Can’t tell	Can’t tell	Weak	Weak
Lanaspa 2015	Mod	Mod	Mod	N/A	Mod	Strong	Mod	Mod
Lazzerini 2016	Strong	Mod	Mod	N/A	Mod	Strong	Mod	Mod

le Roux 2015	Weak	Mod	Mod	N/A	Mod	Mod	Mod	Mod
Lima 2015	Strong	Mod	Mod	N/A	Mod	Strong	Strong	Mod
Lozano-Espinosa 2019	Weak	Mod	Can't tell	N/A	Can't tell	Can't tell	can´t tell	Weak
Lufesi 2015	Mod	Mod	Can't tell	N/A	Can't tell	Can't tell	can´t tell	Weak
Ma 2019	Mod	Mod	Mod	N/A	Mod	Strong	Strong	Mod
Macpherson 2019	Strong	Mod	Strong	N/A	Mod	Strong	Strong	Mod
Matthew 2015	Strong	Mod	Mod	N/A	Mod	Strong	Strong	Mod
McCollum 2019	Strong	Strong	Strong	N/A	Strong	Strong	Strong	Strong
McCollum 2020	Strong	Mod	Strong	N/A	Strong	Strong	Strong	Strong
Meligy 2016	Weak	Weak	waek	N/A	Can't tell	Can't tell	Mod	Weak
Mildemberger 2017	Mod	Weak	Can't tell	N/A	Can't tell	Can't tell	Can't tell	Weak
Mohamed 2017	Weak	Mod	Weak	N/A	Mod	Can't tell	Mod	Weak
Morrow 2014	Mod	Mod	Strong	N/A	Mod	Strong	Strong	Mod
Moschovis 2015	Mod	Mod	Mod	N/A	Mod	Strong	Strong	Mod
Moschovis 2013	Mod	Mod	Mod	N/A	Mod	Strong	Strong	Mod
Myers 2019	Mod	Weak	Mod	N/A	Mod	Strong	Mod	Mod
Naheed 2019	Strong	Mod	Mod	N/A	Mod	Strong	Mod	Mod
Nantanda 2014	Mod	Mod	Mod	N/A	Mod	Strong	Strong	Mod
Nathan 2014	Mod	Mod	Mod	N/A	Mod	Mod	Strong	Mod
Negash 2019	Strong	Mod	Mod	N/A	Mod	Strong	Strong	Mod
Nemani 2016	Strong	Mod	Mod	N/A	Mod	Strong	Mod	Mod
Nguyen 2019	Strong	Mod	Mod	N/A	Mod	Strong	Strong	Mod
Nimdet 2017	Mod	Mod	Mod	N/A	Mod	Strong	Mod	Mod
O'Callaghan-Gordo 2011	Mod	Mod	Mod	N/A	Mod	Strong	Strong	Mod
Ofman 2020	Mod	Mod	Mod	N/A	Mod	Strong	Strong	Mod
Olsen 2010	Mod	Weak	Weak	N/A	Mod	Mod	Mod	Weak
Onyango 2012	Mod	Mod	Weak	N/A	Mod	Mod	Mod	Mod
Orimadegun 2013	Mod	Mod	Mod	N/A	Mod	Strong	Strong	Mod
Pagano 2018	Weak	Mod	Can't tell	N/A	Mod	Can't tell	Can't tell	Weak
Pale 2017	Weak	Weak	Weak	N/A	Mod	Strong	Mod	Weak
Pedraza-Bernal 2016	Mod	Mod	Mod	N/A	Mod	Strong	Strong	Mod
Pulsan 2019	Weak	Mod	Mod	N/A	Strong	Strong	Strong	Mod
Rajatonirina 2013	Mod	Mod	Mod	N/A	Mod	Strong	Strong	Mod
Ramachandran 2012	Mod	Weak	Weak	N/A	Weak	Mod	Mod	Weak

Ramakrishna 2012	Mod	Strong	Strong	N/A	Strong	Strong	Strong	Strong
Reed 2012	Strong	Strong	Strong	N/A	Strong	Strong	Strong	Strong
Rose 2010	Weak	Mod	Weak	N/A	Can’t tell	Can’t tell	Mod	Weak
Saghafian-Hedengren 2017	Mod	Mod	Mod	N/A	Mod	Strong	Strong	Mod
Saha 2016	Mod	Mod	Mod	N/A	Mod	Strong	Strong	Mod
Saleh 2018	Mod	Mod	Weak	Weak	Mod	Strong	Mod	Weak
Shan 2019	Mod	Mod	Strong	N/A	Mod	Strong	Strong	Mod
Solis-Chaves 2018	Mod	Mod	Mdoeate	N/A	Mod	Can’t tell	Mod	Mod
Srinivasan 2012	Strong	Strong	Strong	Strong	Strong	Strong	Strong	Strong
Sudarwati 2014	Weak	Mod	Can’t tell	N/A	Weak	Can’t tell	Can’t tell	Weak
Suntarattiwong 2011	Weak	Mod	Weak	N/A	Weak	Weak	Mod	Weak
Sutcliffe 2016	Strong	Mod	Strong	N/A	Strong	Strong	Strong	Strong
Suzuki 2012	Strong	Mod	Strong	N/A	Mod	Mod	Strong	Mod
Tapisiz 2011	Mod	Mod	Mod	N/A	Mod /Weak	Mod	Mod	Mod
Tomczyk 2019	Mod	Mod	Strong	N/A	Mod	Strong	Strong	Mod
Tuti 2017	Strong	Mod	Strong	N/A	Mod	Strong	Strong	Mod
Walk 2016	Mod	Mod	Mod	N/A	Mod	Strong	Strong	Mod
Wandeler 2015	Mod	Mod	Mod	N/A	Mod	Strong	Strong	Mod
Webb 2012	Strong	Mod	Strong	N/A	Mod	Strong	Strong	Mod
Wilson 2017	Mod	Mod	Mod	Weak	Mod	Strong	Strong	Mod
Zabihullah 2017	Strong	Mod	Mod	N/A	Mod	Strong	Strong	Mod
Zampoli 2011	Weak	Mod	Mod	N/A	Mod	Can’t tell	Strong	Mod
Zeeshan 2020	Mod	Mod	Can’t tell	N/A	Mod	Can’t tell	Can’t tell	Weak
Zhang 2013	Mod	Mod	Mdoerate	N/A	Mod	Strong	Strong	Mod
Zhang 2011	Mod	Mod	Mod	N/A	Mod	Strong	Weak	Mod
Zhang 2020	Weak	Mod	Mod	N/A	Mod	Strong	Strong	Mod
Zhu 2012	Weak	Weak	Mod	N/A	Mod	Strong	Can't tell	Weak
Zidan 2014	Weak	Mod	Mod	N/A	Mod	Strong	Strong	Mod
Zurita-Cruz 2020	Mod	Mod	Mod	N/A	Weak	Strong	Strong	Mod

Table S2 – Extended results tables showing summary associations of demographic, clinical and laboratory factors with mortality, treatment failure, hypoxaemia and ICU admission.

1. **Associations with mortality**

Table S2_1a. Associations of mortality with demographic features

Table S2_1b. Associations of mortality with clinical features

Table S2_1c. Associations of mortality with comorbidities

Table S2_1d. Associations of mortality with markers of malnutrition

Table S2_1e. Associations of mortality with aetiology and laboratory findings

Table S2_1f. Other investigated factors for associations with mortality

Table S2_1g. Summary table of number of studies showing positive, no, or negative correlation of specific factors with mortality

2. **Associations with treatment failure**

Table S2_2a. Associations of treatment failure with demographic features

Table S2_2b. Associations of treatment failure with clinical features

Table S2_2c. Associations of treatment failure with comorbidities

Table S2_2d. Associations of treatment failure with markers of malnutrition

Table S2_2e. Associations of treatment failure with aetiology and laboratory findings

Table S2_2f. Other investigated factors for associations with treatment failure

Table S2_2g. Summary table of number of studies showing positive, no, or negative correlation of specific factors with treatment failure

3. **Associations with hypoxaemia**

Table S2_3a. Associations of hypoxaemia with demographic features

Table S2_3b. Associations of hypoxaemia with clinical features

Table S2_3c. Associations of hypoxaemia with comorbidities

Table S2_3d. Associations of hypoxaemia with markers of malnutrition

Table S2_3e. Associations of hypoxaemia with aetiology and laboratory findings

Table S2_3f. Other investigated factors for associations with hypoxaemia

Table S2_3g. Summary table of number of studies showing positive, no, or negative correlation of specific factors with hypoxaemia

4. **Associations with ICU admission**

Table S2_4a. Associations of ICU admission with demographic features

Table S2_4b. Associations of ICU admission with clinical features

Table S2_4c. Associations of ICU admission with comorbidities

Table S2_4d. Associations of ICU admission with markers of malnutrition

Table S2_4e. Associations of ICU admission with aetiology and laboratory findings

Table S42_f. Other investigated factors for associations with ICU admission

Table S42_g. Summary table of number of studies showing positive, no, or negative correlation of specific factors with ICU admission

Table S2a - Summary of number of studies showing positive, no, or negative correlation of specific factors with MORTALITY

Factor	Number of studies showing positive, neutral, or negative association with MORTALITY			Total number of studies
	Positive association (P<0.05)	No association	Negative association (P<0.05)	
**Demographic**				
Age <12m	27	14	0	41
Female sex	9	27	0	36
Inadequate immunisation	9	5	0	14
Low socio-economic status	4	4	0	8
Suboptimal breastfeeding	4	3	0	7
Low parental education	4	3	0	7
Study/hospital site	6	0	0	6
Smoking exposure at home	3	3	0	6
Crowding at home	0	4	0	4
Indoor air pollution	2	2	0	4
Malaria endemic area	3	0	0	3
Ethnicity	0	2	0	2
Limited access to quality water	2	0	0	2
Lack of sewage or latrine at home	1	1	0	2
Rural compared to urban	1	1	0	2
Low maternal age	1	0	0	1
Past parental experience of child loss	1	0	0	1
Maternal disease in pregnancy	1	0	0	1
Maternal TB	1	0	0	1
Antenatal care and birth spacing	0	0	1	1
Late referral	1	0	0	1
Distance of home to health centre	0	0	0	0

Factor	Number of studies showing positive, neutral, or negative association with MORTALITY			Total number of studies
	Positive association (P<0.05)	No association	Negative association (P<0.05)	
**Clinical**				
Hypoxaemia	32	2	0	34
HIV Infected	23	6	0	29
WHO Severe pneumonia	26	2	0	28
Severe malnutrition otherwise defined	16	4	0	20
Comorbidities (any)	14	5	0	19
Decreased conscious level	17	0	0	17
Tachypnoea	13	4	0	17
Anaemia	9	6	0	15
Wheeze	2	2	10	14
Fever	4	7	3	14
Weight for age Z score -2 to -3	10	4	0	14
Weight for age Z score <-3	13	1	0	14
Chest indrawing	8	5	0	13
Congenital Heart Disease	11	2	0	13
Malaria diagnosis	3	7	3	13
Weight for height Z score <-3	8	1	0	9
Cyanosis	7	1	0	8
Convulsions	6	2	0	8
Diarrhoea	5	3	0	8
Pallor	6	1	0	7
Weight for height Z score -2 to -3	6	1	0	7
Crepitations/crackles on auscultation	3	3	0	6
Tachycardia	3	1	2	6
HIV exposed uninfected	3	3	0	6
Ex-preterm or low birth weight	1	5	0	6

Factor	Number of studies showing positive, neutral, or negative association with MORTALITY			Total number of studies
	Positive association (P<0.05)	No association	Negative association (P<0.05)	
**Clinical (continued)**				
Height for age Z score -2 to -3	1	5	0	6
Heart failure	3	1	0	4
Asthma	0	2	2	4
Other chronic lung disease	2	2	0	4
Neurodevelopmental	2	2	0	4
Ex-low birth weight	1	3	0	4
Height for age Z score <-3	3	1	0	4
Hypotension	2	1	0	3
Cough	0	0	2	2
Signs of shock	2	0	0	2
Meningitis	2	0	0	2
Sepsis	1	1	0	2
Previous admission for pneumonia	2	0	0	2
Stridor	1	0	0	1
**Laboratory and aetiology**				
Bacterial (undifferentiated)	9	13	0	22
Viral (undifferentiated)	1	11	4	16
RSV	0	5	5	10
CXR Consolidation	7	2	0	9
Leucocytosis	4	4	1	9
Raised CRP	2	4	0	6
Pneumocystis jirovecii	4	0	0	4
Influenza (undifferentiated)	0	4	0	4
Adenovirus	1	3	0	4
Parainfluenza	2	2	0	4

Factor	Number of studies showing positive, neutral, or negative association with MORTALITY			Total number of studies
	Positive association (P<0.05)	No association	Negative association (P<0.05)	
**Laboratory / aetiology (continued)**				
Human Metapneumovirus	0	2	2	4
Leukopenia	4	0	0	4
Pleural effusion on CXR	3	0	0	3
Pneumococcus	2	1	0	3
Staphylococcus	3	0	0	3
Influenza A	3	0	0	3
H1N1 Influenza	2	1	0	3
Rhinovirus	0	3	0	3
Hypoglycaemia	3	0	0	3
Raised Lactate	3	0	0	3
Enterovirus	0	2	0	2
Pneumothorax on CXR	2	0	0	2
Thrombocytosis	0	2	0	2
Thrombocytopaenia	2	0	0	2
Acidaemia	2	0	0	2
Raised Procalcitonin	2	0	0	2
High IL1RA	2	0	0	2
High IL-6	1	1	0	2
Pertussis	0	1	0	1
Influenza B	1	1	0	1
Measles	0	1	0	1
Low zinc	1	0	0	1
Hyperglycaemia	1	0	0	1
Electrolyte abnormality	1	0	0	1
Low bicarbonate	1	0	0	1
**Factor**	**Number of studies showing positive, neutral, or negative association with MORTALITY**			

	Positive association (P<0.05)	No association	Negative association (P<0.05)	Total number of studies
**Laboratory / aetiology (continued)**				
Raised ESR	1	0	0	1
High IL-8	1	0	0	1
High IL-17	1	0	0	1
High MIP-1a	1	0	0	1
High CK-MB Fraction	1	0	0	1
High copeptin	1	0	0	1
High Angiopoietin 2: Angiopoietin 1	1	0	0	1
Low Angiopoietin-1	1	0	0	1
Low CCL22	1	0	0	1
CK	0	1	0	1
Creatinine	0	1	0	1
AST	0	1	0	1
ALT	0	1	0	1

CRP, C-reactive protein; CXR, chest x-ray; RSV, respiratory syncytial virus; WHO, World Health Organization;

Table S2b – Summary of number of studies showing positive, no, or negative correlation of specific factors with TREATMENT FAILURE

Factor	Number of studies showing positive, neutral, or negative association with TREATMENT FAILURE			Total number of studies
	Positive association (P<0.05)	No association	Negative association (P<0.05)	
**Demographic**				
Age <12m	4	7	0	11
Female sex	2	5	2	9
Inadequate breastfeeding	1	4	1	6
Inadequate immunisation	2	3	0	5
Smoking exposure at home	0	3	0	3
Low socio-economic status	1	0	0	1
Crowding at home	0	1	0	1
Indoor air pollution	1	0	0	1
**Clinical**				
Hypoxaemia	6	2	0	8
Tachypnoea	4	0	0	4
Severe malnutrition otherwise defined	3	1	0	4
WHO Severe pneumonia	3	0	0	3
Fever	0	3	0	3
Comorbidities (any)	2	1	0	3
HIV Infected	3	0	0	3
Anaemia	2	1	0	3
Weight for height Z score -2 to -3	3	0	0	3
Height for age Z score -2 to -3	2	1	0	3
Wheeze	0	0	2	2
Tachycardia	1	1	0	2
Ex-low birth weight	2	0	0	2
Malaria diagnosis	1	1	0	2
Weight for age Z score -2 to -3	2	0	0	2

Factor	Number of studies showing positive, neutral, or negative association with TREATMENT FAILURE			Total number of studies
	**Positive association (P<0.05)**	**No association**	**Negative association (P<0.05)**	
**Clinical (continued)**				
Weight for age Z score <-3	2	0	0	2
Cyanosis	1	0	0	1
Pallor	0	1	0	1
Crepitations/crackles on auscultation	1	0	0	1
Signs of shock	1	0	0	1
Decreased conscious level	1	0	0	1
Congenital Heart Disease	0	1	0	1
Diarrhoea	1	0	0	1
Weight for height Z score <-3	1	0	0	1
Height for age Z score <-3	1	0	0	1
**Laboratory and aetiology**				
RSV	0	4	0	4
Bacterial (undifferentiated)	2	1	0	3
Leucocytosis	0	3	0	3
Influenza A	1	1	0	2
Raised CRP	1	1	0	2
Viral (undifferentiated)	0	1	0	1
Influenza B	0	1	0	1
Parainfluenza	0	1	0	1
Rhinovirus	0	1	0	1
CXR Consolidation	0	1	0	1
Pleural effusion on CXR	1	0	0	1
Raised Lactate	0	1	0	1
CK	0	1	0	1
Creatinine	0	1	0	1
AST	0	1	0	1

Factor	Number of studies showing positive, neutral, or negative association with TREATMENT FAILURE			Total number of studies
	Positive association (P<0.05)	No association	Negative association (P<0.05)	
**Lab / aetiology (continued)**				
ALT	0	1	0	1
Hypoglycaemia	0	1	0	1
Hyperglycaemia	0	1	0	1

No included studies reported associations between ICU admission and the following: Low maternal age, Low parental education, Ethnicity, Past parental experience of child loss, Study/hospital site, Malaria endemic area, Limited access to quality water, Lack of sewage or latrine at home, Rural compared to urban, Maternal disease in pregnancy, Maternal TB, Antenatal care and birth spacing, Late referral, Distance from home to health centre, Chest indrawing, Cough, Stridor, Hypotension, Convulsions, Sepsis, Meningitis, HIV exposed uninfected, Other heart failure, Asthma, Other chronic lung disease, Previous admission for pneumonia, Neurodevelopmental, Ex-preterm, Pnemocystis jiroveki, Pneumococcus, Staphylococcus, Pertussis, H1N1 Influenza, Influenza (undifferentiated), Adenovirus, Enterovirus, Human Metapneumovirus, Measles, Lung infiltrates on CXR, Pneumothorax on CXR, Leukopenia, Thrombocytosis, Thrombocytopaenia, Low zinc, Electrolyte abnormality, Acidaemia, Low bicarbonate, Raised Procalcitonin, Raised ESR, High IL1RA, High IL-6, High IL-8, High IL-17, High MIP-1a, High CK-MB Fraction, High copeptin, High Angiopoeitin 2:Angiopoeitin 1 , Low Angiopoeitin-1, Low CCL22,

CRP, C-reactive protein; CXR, chest x-ray; RSV, respiratory syncytial virus; WHO, World Health Organization;

Table S2c – Summary of number of studies showing positive, no, or negative correlation of specific factors with HYPOXAEMIA

**Factor**	**Number of studies showing positive, neutral, or negative association with HYPOXAEMIA**			**Total number of studies**
	**Positive association (P<0.05)**	**No association**	**Negative association (P<0.05)**	
**Demographic**				
Age <12m	5	4	0	9
Female sex	1	4	0	5
Inadequate breastfeeding	2	0	0	2
Inadequate immunisation	1	0	0	1
Low parental education	0	1	0	1
Crowding at home	0	1	0	1
Smoking exposure at home	0	1	0	1
Maternal diet in pregnancy	1	0	0	1
Living at high altitude	1	0	0	1
Low maternal age	0	0	0	0
Low socio-economic status	0	0	0	0
Ethnicity	0	0	0	0
Study/hospital site	0	0	0	0
Indoor air pollution	0	0	0	0
**Clinical**				
Cyanosis	4	0	0	4
Chest indrawing	3	1	0	4
Wheeze	1	3	0	4
Tachypnoea	3	0	0	3
Comorbidities (any)	3	0	0	3
Severe malnutrition otherwise defined	0	3	0	3
WHO Severe pneumonia	2	0	0	2
Cough	0	2	0	2
Fever	0	2	0	2

Factor	Number of studies showing positive, neutral, or negative association with HYPOXAEMIA			Total number of studies
	Positive association (P<0.05)	No association	Negative association (P<0.05)	
**Clinical (continued)**				
Ex-preterm	1	1	0	2
Weight for age Z score -2 to -3	1	1	0	2
Weight for height Z score <-3	1	1	0	2
Crepitations/crackles on auscultation	0	1	0	1
Tachycardia	1	0	0	1
Convulsions	0	1	0	1
HIV Infected	0	1	0	1
Congenital Heart Disease	0	1	0	1
Anaemia	1	0	0	1
Asthma	0	1	0	1
Other chronic lung disease	0	1	0	1
Ex-low birth weight	0	1	0	1
Diarrhoea	0	1	0	1
Malaria diagnosis	1	0	0	1
Weight for age Z score <-3	1	0	0	1
Weight for height Z score -2 to -3	1	0	0	1
Height for age Z score -2 to -3	1	0	0	1
**Laboratory and aetiology**				
RSV	3	1	0	4
Influenza (undifferentiated)	2	1	0	3
Pleural effusion on CXR	3	0	0	3
Bacterial (undifferentiated)	1	1	0	2
Viral (undifferentiated)	1	1	0	2
Raised CRP	0	2	0	2
Pnemocystis jiroveki	1	0	0	1
Pneumococcus	0	1	0	1

Factor	Number of studies showing positive, neutral, or negative association with HYPOXAEMIA			Total number of studies
	Positive association (P<0.05)	No association	Negative association (P<0.05)	
**Lab / aetiology (continued)**				
Staphylococcus	0	1	0	1
Influenza A	0	1	0	1
Influenza B	0	1	0	1
H1N1 Influenza	0	1	0	1
Adenovirus	0	1	0	1
Enterovirus	0	1	0	1
Human Metapneumovirus	1	0	0	1
Rhinovirus	0	1	0	1
Coronavirus	0	1	0	1
Parechovirus	0	1	0	1
Bocavirus	0	1	0	1
CXR Consolidation	1	0	0	1
Leucocytosis	1	0	0	1
Low vitamin D	1	0	0	1
Low serum albumin	0	1	0	1
Raised Procalcitonin	1	0	0	1
Raised Lactate	1	0	0	1

No included studies reported associations between hypoxaemia and the following: Low maternal age, Low socio-economic status, Ethnicity, Study/hospital site, Indoor air pollution, Pallor, Stridor, Hypotension, Signs of shock, Decreased conscious level, Sepsis, Meningitis, HIV exposed uninfected, Other heart failure, Previous admission for pneumonia, Neurodevelopmental, Height for age Z score <-3, Pertussis, Parainfluenza, Measles, Lung infiltrates on CXR, Pneumothorax on CXR.

CRP, C-reactive protein; CXR, chest x-ray; RSV, respiratory syncytial virus; WHO, World Health Organization;

Table S2d – Summary of number of studies showing positive, no, or negative correlation of specific factors with ICU ADMISSION

**Factor**	**Number of studies showing positive, neutral, or negative association with ICU ADMISSION**			**Total number of studies**
	**Positive association (P<0.05)**	**No association**	**Negative association (P<0.05)**	
**Demographic**				
Age <12m	3	3	0	6
Female sex	0	1	2	3
Low parental education	2	0	0	2
Inadequate breastfeeding	1	0	0	1
Inadequate immunisation	0	1	0	1
Low maternal age	0	1	0	1
Low socio-economic status	1	0	0	1
Crowding at home	0	1	0	1
Smoking exposure at home	0	1	0	1
Indoor air pollution	1	0	0	1
Attends nursery	0	0	1	1
Smoking in pregnancy	0	1	0	1
**Clinical**				
Congenital Heart Disease	5	0	0	5
Ex-preterm	1	3	0	4
Fever	0	1	2	3
Other chronic lung disease	2	1	0	3
Hypoxaemia	1	1	0	2
Chest indrawing	1	1	0	2
Cough	0	1	1	2
Wheeze	2	0	0	2
Comorbidities (any)	0	2	0	2
Anaemia	1	1	0	2
Previous admission for pneumonia	2	0	0	2

Factor	Number of studies showing positive, neutral, or negative association with ICU ADMISSION			Total number of studies
	Positive association (P<0.05)	No association	Negative association (P<0.05)	
**Clinical (continued)**				
Ex-low birth weight	0	2	0	2
Weight for age Z score -2 to -3	2	0	0	2
WHO Severe pneumonia	1	0	0	1
Cyanosis	0	1	0	1
Tachypnoea	1	0	0	1
Crepitations/crackles on auscultation	1	0	0	1
Stridor	0	1	0	1
Sepsis	1	0	0	1
Pulmonary hypertension	1	0	0	1
Asthma	0	1	0	1
Neurodevelopmental	1	0	0	1
Malnutrition otherwise defined	1	0	0	1
**Laboratory and aetiology**				
RSV	2	8	0	10
Bacterial (undifferentiated)	2	2	0	4
Human Metapneumovirus	0	4	0	4
Influenza (undifferentiated)	0	3	0	3
Adenovirus	0	3	0	3
Viral (undifferentiated)	0	2	0	2
H1N1 Influenza	1	1	0	2
Enterovirus	0	2	0	2
Rhinovirus	0	2	0	2
Bocavirus	0	2	0	2
Leucocytosis	1	1	0	2
Raised CRP	2	0	0	2
Pnemocystis jiroveki	0	1	0	1

Factor	Number of studies showing positive, neutral, or negative association with ICU ADMISSION			Total number of studies
	Positive association (P<0.05)	No association	Negative association (P<0.05)	
**Lab / aetiology (continued)**				
Parainfluenza	0	1	0	1
Coronavirus	0	1	0	1
Pneumothorax on CXR	1	0	0	1
Leukopenia	1	0	0	1
Low zinc	1	0	0	1
Electrolyte abnormality	0	1	0	1
Low bicarbonate	0	0	1	1
High IL-1β	1	0	0	1
IL6-174 genotype CC or GC (ref GG)	1	0	0	1
High Pro-adrenomedullin	1	0	0	1

No included studies reported associations between ICU admission and the following: Ethnicity, Pallor, Tachycardia, Hypotension, Signs of shock, Decreased conscious level, Convulsions, Meningitis, HIV Infected, HIV exposed uninfected, Other heart failure, Diarrhoea, Malaria diagnosis, Weight for age Z score <-3, Weight for height Z score -2 to -3, Weight for height Z score <-3, Height for age Z score -2 to -3, Height for age Z score <-3 *Pneumococcus, Staphylococcus, Pertussis, Influenza A, Influenza B, Measles,* CXR Consolidation, Lung infiltrates on CXR, Pleural effusion on CXR, Thrombocytosis, Thrombocytopaenia, Hypoglycaemia, Hyperglycaemia, Acidaemia, Raised Procalcitonin, Raised Lactate, Raised ESR.

CRP, C-reactive protein; CXR, chest x-ray; RSV, respiratory syncytial virus; WHO, World Health Organization;

Table S3 –Extended results tables showing associations of demographic, clinical and laboratory factors with mortality, treatment failure, hypoxaemia and ICU admission, by individual study

5. **Associations with mortality**

Table S1a. Associations of mortality with demographic features

Table S1b. Associations of mortality with clinical features

Table S1c. Associations of mortality with comorbidities

Table S1d. Associations of mortality with markers of malnutrition

Table S1e. Associations of mortality with aetiology and laboratory findings

Table S1f. Other investigated factors for associations with mortality

Table S1g. Summary table of number of studies showing positive, no, or negative correlation of specific factors with mortality

6. **Associations with treatment failure**

Table S2a. Associations of treatment failure with demographic features

Table S2b. Associations of treatment failure with clinical features

Table S2c. Associations of treatment failure with comorbidities

Table S2d. Associations of treatment failure with markers of malnutrition

Table S2e. Associations of treatment failure with aetiology and laboratory findings

Table S2f. Other investigated factors for associations with treatment failure

Table S2g. Summary table of number of studies showing positive, no, or negative correlation of specific factors with treatment failure

7. **Associations with hypoxaemia**

Table S3a. Associations of hypoxaemia with demographic features

Table S3b. Associations of hypoxaemia with clinical features

Table S3c. Associations of hypoxaemia with comorbidities

Table S3d. Associations of hypoxaemia with markers of malnutrition

Table S3e. Associations of hypoxaemia with aetiology and laboratory findings

Table S3f. Other investigated factors for associations with hypoxaemia

Table S3g. Summary table of number of studies showing positive, no, or negative correlation of specific factors with hypoxaemia

8. **Associations with ICU admission**

Table S4a. Associations of ICU admission with demographic features

Table S4b. Associations of ICU admission with clinical features

Table S4c. Associations of ICU admission with comorbidities

Table S4d. Associations of ICU admission with markers of malnutrition

Table S4e. Associations of ICU admission with aetiology and laboratory findings

Table S4f. Other investigated factors for associations with ICU admission

Table S4g. Summary table of number of studies showing positive, no, or negative correlation of specific factors with ICU admission

Table S3a – Associations of mortality with demographic features

YES = significant association (P<0.05), NO = investigated but no significant association found (P>0.05)

Author & Year	Child's age (months)	Female sex	Breastfeeding status	Immunisation Status	Maternal Age	Parental Education	Socio-economic status	Other demographic factors
Agweyu 2018	YES<br><12m CFR 8.0%<br>>12m CFR 2.7%, RR 3.0 (aRR 2.5)	YES - RR 1.4 (aRR 1.5)		YES<br>Not up to date OR 1.6				
Agweyu 2018	YES<br><12m CFR 1.9% OR 4.5<br>>12m CFR 0.4%	NO<br>P 0.8		NO<br>Immunised OR 0.3<br>P 0.06				
Ahmed 2018	YES<br><6 m<br>CFR 19.5%<br>6-11m 22.6%<br>12-23m 13.7%<br>24-59 m 11.6%<br>P 0.049	NO<br>P 0.16						
Araya 2016	YES<br><6m OR 2.2<br>7-59m OR 0.36 (0.2–0.6)<br>60m - 15years OR 2.1							
Atwa 2015	NO<br><2y P 0.77	NO<br>P 0.23						
Ayieko 2012	YES<br>2-11m OR 1 (ref)<br>12-24m OR 0.41 (aOR 0.39)<br>25-59m OR 0.42 (aOR 0.42)	NO						
Bills 2020	YES<br>1m-12m CFR 8.05%<br>1-4y CFR 3% >4y CFR 3.3%<br>P 0.03	NO<br>P 0.7				NO Parental literacy P 0.15	YES - by caste<br>Other caste CFR 2.5%<br>Non-Other Caste CFR 6.4%<br><br>OR 2 aOR 1.87<br><br>NO - Economic status P 0.45.	

Bokade 2015	YES<br><6m higher risk P 0.03	NO<br>P 0.52	YES<br>P0.01	YES<br>incomplete immunisation<br>aOR 12.3			NO<br>P 0.95	
Boukari 2011	YES<br>All deaths in <6m olds							
Champatiray 2017	NO<br>P 0.64	NO<br>P 0.43	NO<br>P 0.77	NO<br>P 0.17			NO<br>P 0.69	
Daga 2014	NO	NO						
Dembele 2019	YES<br>2-5m CFR 7.0% OR 1.73 (aOR 1.77)<br>6-11m CFR 4.5% OR 1.29<br>(aOR 1.29)<br>12-35m CFR 3.4% OR 0.97 (aOR 0.96)<br>36-59m 3.5% OR 1 (ref)	NO<br>P 0.49					NO<br>P 0.49	
Divecha 2019	NO for patients without cardiac disease P 0.84<br><br>YES if cardiac disease P 0.001			YES<br>incompletely immunised CFR 28% (CFR 17.1% for respiratory cases overall)<br>P 0.016				
Emukule 2014	YES<br>3-12m OR 1.6<br>13-23m OR 1 (ref)							
Enarson 2014	YES<br>2-11m CFR 11.9%<br>OR 1.86<br>12-59m CFR 6.4%							
Ezeonu 2015		YES<br>Male CFR 8.6%<br>Female CFR 6.1%<br>P 0.03<br><br>HOWEVER If including all DAMA patients female CFR 14.1%		YES - Inadequate immunisation higher risk<br>P <0.01				

		male CFR 9.3%						
Ferolla 2013	YES - All deaths <12m							
Ferreira 2014	NO<br>< 12m P 0.25	NO<br>P 0.5.						
Gallagher 2020	YES<br>1-11 m OR 1.37 (aOR 2.2)	YES<br>OR 1.76 (aOR 1.99)		NO<br>DTP P0.34<br>Hib P 0.36, PCV P 0.75				
Graham 2011	YES<br><6m CFR 23.6%<br>6-12m CFR 14.1%<br>1-5y CFR 0.8%<br>P <0.0001.							
Hasan 2014	28d-6m CFR 1.4%<br>6-23m CFR 0.3%<br>24-59m CFR 0.2%							
Hooli 2016		YES<br>OR 1.25						
Hutton 2019	NO<br><6m P 0.5	NO<br>P 0.15.	YES (survive vs died)<br>Breastfed aOR 4.38 (0.92 to 20.82) P 0.063.<br><br>Exclusive formula fed OR 0.31 (0.10 to 0.96)<br>P 0.043.<br><br>Mixed feeding OR 0.36 (0.14 to 0.94)<br>P 0.038				YES - Informal vs formal housing<br>OR 2.87<br>aOR 11.87<br><br>NO – Higher vs lower income<br>P 0.695	
Ibraheem 2020		NO<br>P 0.06						
Jain 2018		NO<br>P 0.90						
Jakhar 2018							NO<br>P 0.112	
Julien 2020				YES? Only 2 of 6 deaths fully immunised, relative				

				to 75.7% of participants. No ORs				
Kelly 2019			NO P0.17					
Kuti 2013	YES<br><12m increased risk<br>P 0.005<br>No longer significant on multivariate analysis	NO		NO				
Lazzerini 2016		YES<br>2-11m OR 1·32 (aOR 1·51)<br>12-59m OR 1·27 (aOR 1·35)						
le Roux 2015	YES? – both deaths in infants 6 weeks or less							
Ma 2019	NO<br>P 0.058	NO<br>P0.2						
Macpherson 2019	YES<br>10- 14y CFR 14.05% OR 2.38 (aOR 2.78)<br>5-9y CFR 6.43%	NO						
Matthew 2015	YES<br><12m higher risk							
McCollum 2019		NO<br>P 0.62						
Naheed 2019	YES<br>2-11m OR 1.88 (aOR 1.5)	YES<br>Female CFR 4.7% OR 1.3 (aOR 1.31)<br>Male CFR 3.7%				YES - father completed <10 years of schooling OR 1.93 (aOR 1.77)		
Nantanda 2014	NO	NO	NO			NO		
O'Callaghan-Gordo 2011	NO	NO						
Ofman 2020	YES - all 15 deaths were in children <12 months of age							
Pale 2017	YES - 2 children died, one 6m one 3m.							
Rajatonirina 2013	YES - all patients who died were under 18 months old and 67%							

	were under 6 months old							
Ramachandran 2012	YES<br>1-6m OR 2.18 (aOR 1.15)	NO						
Ramakrishna 2012	YES<br><12 m OR 28.5 aOR 25.8							
Reed 2012	YES<br>3-12 m OR 5.5 (aOR 6.0) (in HIV +ve<br>OR 2.4 (in HIV -ve)	NO		NO				
Saha 2016	YES<br>2-5m<br>OR 3.63, (aOR 3.52);<br>6-11m OR 2.1, (aOR 2.58)<br>12-59m ref	NO		YES - fully vaccinated OR 0.23, partially vaccinated OR 0.58		YES - paternal education level higher than secondary OR 0.23, aOR 0.49, maternal education level higher than secondary OR 0.25		
Shan 2019	YES<br>12m - 24m OR 1.36 (aOR 1.65)<br>6m to 12m OR 1.76 (aOR 1.96) 29d to 6m OR 2.21 (aOR 1.59)							
Sonego 2015**<br><br>Systematic review – individual study data included in synthesis	YES<br><12m pOR 2.35 (ref 12-59m)<br><6m pOR 2.22 (ref 12-59m)<br><6m ref 7-59m non-significant	YES - pOR 1.15 (23 studies)	YES - inadequate vs adequate pOR 1.79 (12 studies)	YES - pOR 0.46 (12 studies)	YES - pOR 1.84 for younger age	YES - pOR 1.43 for lower vs higher	YES - pOR 1.62 for lower income or social class of father vs higher (9 studies)	
Srinivasan 2012	NO							
Sutcliffe 2016	NO							
Tomczyk 2019	NO	NO	YES - Breastfed in first 2 years of life OR 0.6	YES - Received ≥1 dose of DPt/Hib, Hep B vaccine<br>OR 0.6<br><br>NO		YES - parent completed primary school OR 0.6	YES – low family income<br>OR 2.2	NO - Ethnicity

				Received influenza vaccine in past 6 months OR 0.6 P >0.05				
Tuti 2017	YES OR 2.89	YES OR 1.52						
Walk 2016	NO	NO						
Wilson 2017	YES median age 12.5m in those who died vs 17.7m in those who survived P 0.015	NO						
Zabihullah 2017	NO P 0.17 (0.24 adjusted)	NO P 0.15		YES - BCG vaccine received OR 0.39<br><br>>1 dose pentavalent vaccine receieved OR 0.53<br><br>NO - Measles vaccine OR 0.4 but P 0.11		NO - maternal literacy OR 0.56 P 0.17		NO - Ethnicity
Zhang 2013	NO	NO						
Zurita-Cruz 2020		YES OR 1.34						

Table S3b - Associations of mortality with clinical features

YES = significant association (P<0.05), NO = investigated but no significant association found (P>0.05)

**Author & Year**	**WHO defined severe pneumonia**	**Hypoxaemia (SaO2 <90% except where specified)**	**Cyanosis**	**Tachypnoea (per WHO definition except where specified)**	**Chest indrawing**	**Cough**	**Wheeze**	**Other auscultation findings**	**Tachycardia (per WHO definition except where specified)**	**Pallor**	**Fever (temperature ≥38°C except where specified)**	**Altered conscious state**
Abdulkadir 2015		YES<br>CFR 20.5%<br>RR 48.1<br><br>Fatal cases mean SpO2 78.3%<br>Survived cases mean SpO2 91.5%										
Agweyu 2018	YES<br>Severe CFR 14.2%<br>Non-severe CFR 2.7%<br>RR 5.3			YES for RR >10 above upper limit of normal<br>CFR with 10%<br>CFR without 4.1%<br>RR 2.4	YES<br>CFR with 6.3%<br>CFR without 2.8%<br>RR 2.3<br>aRR 2.0					YES for severe pallor<br>CFR with 17.8%<br>CFR without 3.6%<br>RR 4.8<br>aRR 5.6	YES<br>Temp >39 CFR with 7.8%<br>CFR without 4.1%<br>RR 1.9	
Agweyu 2018							NO<br>P 0.24			NO<br>P 0.6		
Alohan 2019		YES<br>OR 11.1										
Araya 2016		YES<br>OR 11 6		YES<br>OR 3.2							YES<br>Apyrexia OR 2.3	YES - Seizures OR 10.6, GCS <13 OR 324,
Atwa 2015	YES<br>OR 1.84											
Ayieko 2012	YES<br>Severe OR 2.59,<br>Very severe OR 9.12,<br>aOR 9.17				YES<br>OR 2.59							

	Non-severe<br>CFR 4.8%<br>Severe CFR 15.1%											
Benet 2017		YES<br>HR 4.8<br>aHR 4.3<br>Median SpO2 91% (Died) vs 95% (Survived)										
Bills 2020		YES for SpO2<93%<br>OR 6.3<br>aOR 2.56		YES<br>OR 2.29	YES<br>*retractions*<br>OR 3.04<br>*Accessory muscle use*<br>OR 4.20<br>*Retractions, accessory muscle use & tachypnoea*<br>OR 5.38<br>*Respiratory distress*<br>OR 6.8<br>aOR 2.67							YES - abnormal mental status<br>OR 7.39 aOR 2.14
Bokade 2015	YES<br>Non-severe<br>CFR 6.3%<br>Severe CFR 15.9%	YES<br>CFR 21.2%<br>P 0.014	YES	YES for RR >10 above upper limit of normal<br>P 0.02	YES<br>CFR 20%					YES - 16/107 died, P0.02		YES - altered consciousness,
Champatiray 2017	YES<br>Severe CFR 38.6%<br>Non-severe CFR 10.7%											

Cohen 2015		YES – for “requiring supplementary-oxygen”										
Cotes 2015												
Daga 2014					NO						NO	YES - Altered conscious level, convulsions
Dembele 2019		YES<br>OR 4.09<br>aOR 5.06	YES<br>OR 28.24<br>aOR 27.58	YES<br>OR 2.04 aOR 1.89	NO<br>P 0.73		YES - OR 1.35<br>aOR 1.55	NO for rales<br>P 1.0	YES<br>OR 1.08 aOR 2.09		YES<br>Temp >38.5 OR 2.15<br>aOR 2.5	YES - Sensorial changes OR 9.61 aOR 8.31<br>GCS <9 OR 45.0
Emukule 2014	YES<br>OR 4.2	YES<br>OR 1.8		YES<br>OR 0.7	YES - OR 3.7 aOR 2.2 (only significant in under 2 year olds)		YES - OR 2.3	YES for stridor<br>OR 2.7				YES - Unconscious OR 3.1 aOR 2.3<br>Lethargic OR 2.1<br>VP or U on AVPU scale OR 13.5 aOR 8.0
Enarson 2015	YES<br>OR 5.36<br>aOR 4.05	YES for "required supplementary oxygen"<br>OR 4.68<br>aOR 2.16										
Enarson 2014	YES<br>Severe CFR 21.9%<br>Non-severe CFR 4.8%											
Fagbohun 2020		YES			YES for presence of respiratory distress					YES - no OR or P given		YES - Convulsions, confusion, coma,

Ferreira 2014	YES<br>OR 1.3<br>aOR 3.2											
Gallagher 2020	YES - at least one danger sign OR 4.80	YES for SpO2 <92%<br>OR 3.18<br>aOR 2.55	YES - OR 2.86	NO	YES<br>OR 0.31	YES - OR 0.43<br>aOR 0.48	YES<br>OR 0.37	NO for crackles or rales or crepitations P 0.3<br>Stridor<br>P0.22	NO		NO	YES – Responds only to voice OR 6.06, Responds only to Pain OR 6.28, Unresponsive OR 17.6. Any decreased responsiveness OR 7.10,
Gowraiah 2014							YES<br>No deaths in patients with wheezy disease					YES - unresponsive or only responds to pain, sensitivity / specificity 87.5/94.2
Graham 2019		YES<br>OR 6.0<br>aOR 7.1										
Graham 2011	YES<br>Severe CFR 21.8%<br>Non-severe CFR 5.9%	YES<br>SpO2 80-89% CFR 11.9%<br>SpO2 ≥90% CFR 3.8%										
Hooli 2016		YES SpO2 <90 OR 5.04<br>SpO2 90-92 OR 1.54					YES<br>OR 0.71					YES - unconscious OR 5.68
Indriyani 2018	YES											
Jain 2018			YES<br>45% of patients who died									YES - Abnormal sensorium OR

			3% of patients who survived<br>OR 26.3<br>aOR 10.2									19.87 (aOR 21.78),
Kelly 2019	YES<br>p=0.003											
Kim 2019	YES	YES										
Kuti 2013		NO<br>OR 3.055, p=0.22	NO								YES<br>*Temp <36.5*<br>33.3% of patients who died<br>2.4% of patients who survived<br><br>Hyperpyrexia non-significant.	YES – somnolence, lethargy,
Laman 2013		YES - all deaths hypoxaemic	YES	YES								YES - drowsiness
Lazzerini 2016	YES<br><br>*2-11m*<br>CFR 16·5%<br>OR 9·44<br>aOR 12·22<br><br>*12-59m*<br>CFR 6·1%<br>OR 4·30<br>aOR 9·38			NO							YES<br>*2-12m*<br>OR 0·94<br>*12-59m*<br>OR 0·83	
Lazzerini 2015**<br><br>Systematic review – individual study data included in synthesis		YES<br>pOR 5.47<br>(13 studies)<br>I2=65.3%<br><br>Subgroup analysis did not affect results.										

Lima 2015	NO<br>P 0.6	YES – SpO2 <92 in 100% of deaths, 48.1% of survivors										
Lufesi 2015	YES<br>Severe CFR 11.8%<br>All patients CFR 6.6%											
Ma 2019	NO<br>P 0.22.	NO - P 0.21		NO<br>P>0.99	NO<br>*Chest indrawing*<br>P 0.79<br>*Subcostal retractions*<br>P0.25	YES - 92% of patients who survived 63% of patients who died	NO<br>P 0.37	NO for crackles<br>P 0.052.	YES<br>36% of patients who died, 63% of patients who survived<br>P 0.03		NO<br>P 0.081	YES –<br>Lethargy, altered level of consciousnes s<br>NO - convulsions P 0.57;
Macpherson 2019	YES<br>Severe CFR 13.21%<br>Non-severe CFR 5.05%<br>OR 2.86	YES<br>CFR 13.97%<br>OR 2.76	YES<br>CFR with 20%<br>CFR without 7.25%<br>OR 3.20<br>aOR 3.27	YES<br>CFR with 8.35%<br>CFR without 4.52%<br>OR 1.92<br>aOR 1.87	YES<br>CFR with 9.14%<br>CFR without 6.5%<br>OR 1.45<br>aOR 1.34		YES – CFR with 3.59%<br>CFR without 8.05%<br>OR 0.43<br>aOR 0.52	YES for crackles<br>CFR with 9.62%<br>CFR without 6.26%<br>OR 1.59<br>aOR 1.42		YES for mild/moderat e pallor in areas with low malaria endemicity<br>aOR 2.65<br><br>YES for severe pallor (any area)<br>CFR with 30.1%<br>CFR without 4.3%<br>OR 9.57<br>aOR 8.06	NO<br>P 0.83	YES - Acute neurological disorder OR 6.29<br>Convulsions OR 2.48<br>Reduced consciousnes s OR 6.65 aOR 3.65
Matthew 2015	YES<br>Severe CFR 15.8%	YES for SpO2<95										

	Non-severe<br>CFR 4.7%											
McCollum 2019		YES		YES<br>Mean RR 58.4 in patients who died<br>62.5 in patients who survived	NO				YES<br>Median HR in patients who died 155.4<br>Median HR in patients who survived 165.5		YES	YES - Blantyre coma score <4
McCollum 2020							YES<br>*pneumonia with chest indrawing*<br>OR 0.37<br>aOR 1.02<br><br>*severe pneumonia*<br>Non signficant<br><br>*1–11 months* CFR with 7.3%<br>CFR without 20.0%<br><br>Trend consistent in subgroup analyses	YES in *children without severe malnutrition*<br>With crackles CFR 18.9%<br>CFR without 8.8%<br>P 0.02				
Naheed 2019	YES<br>OR 8.82<br>aOR 6.52											
Nantanda 2014		YES for SpO2<92 - HR 12.2<br>aHR 10.7									NO	
Nguyen 2019	YES											

	Severe<br>CFR<br>4.37%<br>Non-<br>severe<br>CFR<br>0.18%											
Orimadegun 2013		YES<br>aOR 1.52										
Pagano 2018		YES										
Pulsan 2019		YES<br>Mean pre-CPAP SpO2 77.4 in survived<br>65.3 in died.<br>Mean SaO2 after 1 hour of CPAP<br>92 in survived, 83.3 in died										
Ramachandran 2012				NO	NO		YES<br>*Clinically and radiological ly diagnosed*<br>OR 0.26<br><br>NO for *only clinically or only radiological ly diagnosed*					YES<br>Altered consciousnes s OR 5.1 aOR 1.56 in clinically diagnosed, OR 14.5 (aOR 1.56) in radiologically diagnosed, OR 4.69 in clinically and radiologically diagnosed
Ramakrishna 2012	YES<br>OR 4.48<br>aOR 1.56	YES<br>OR 6.52<br>aOR 2.96										
Reed 2012		YES<br><br>*HIV -ve patients*<br>OR 15.2<br>aOR 20.9		YES<br><br>*HIV -ve*<br>OR 2.4<br>*HIV +ve*	YES<br>*Any chest indrawing* OR 7.0 in HIV -ve<br>aOR 4.6		YES<br><br>*HIV -ve*<br>OR 0.1<br>aOR 0.2	YES for crepitations *HIV -ve*<br>OR 2.6<br>*HIV +ve*	YES<br><br>HR >170<br><br>*HIV -ve* non sig		NO	

		HIV +ve patients<br>OR 5.6<br>aOR 4.8		OR 2.1<br><br>YES - RR>20 above upper limit of normal for age<br><br>*HIV -ve*<br>*OR* 4.2<br>*HIV +ve*<br>OR 2.2	OR 3.3 in HIV +ve<br>aOR 2.2<br><br>*Intercostal recession*<br>OR 8.0 in HIV -ve<br>OR 2.4 in HIV +ve		*HIV +ve*<br>OR0.4<br>aOR 0.6	OR 1.0.<br><br>YES for bronchial breathing *HIV -ve*<br>OR 5.4<br>*HIV +ve*<br>OR 1.4	*HIV +ve* OR 1.4			
Saghafian-Hedengren 2017	YES - all 9 deaths had severe pneumonia											
Saha 2016	YES<br>OR 3.02<br>aOR 2.35											
Shan 2019					YES for "respiratory distress"<br>OR 2.55<br>aOR 1.04		YES<br>OR 0.58<br>aOR 0.61				YES<br>OR 0.72<br>aOR 0.75	
Solis-Chaves 2018	YES - All 3 deaths occurred in severe pneumonia cases											
Sonego 2015**<br><br>Systematic review – individual study data included in synthesis	YES<br>pOR 9.42<br>(12 studies)											
Srinivasan 2012		YES for SpO2 <92<br>RR 2.4<br>aRR 1.6										
Sutcliffe 2016	YES<br>OR 2.81<br>aOR 2.79											

Tuti 2017				YES for RR>10 above upper limit of normal OR 2.49						YES for severe pallor OR 4.37 and for moderate pallor OR 4.36	YES OR 1.98	
Wandeler 2015		YES - 100% of deaths vs 36.5% of survivors										
Webb 2012	YES OR 26.5						YES No child with wheeze died					
Wilson 2017		YES - median SaO2 96 in patients who died 98 in patients who survived		YES median RR 62 in patients who died 56 in patients who survived					YES - median HR 163 in patients who died 152 in patients who survived P 0.019		NO P 0.71	
Zhang 2013		YES 93% of patients who died 42% of patients who survived	YES 90% of patients who died 38% of patients who survived	YES for RR > 10 above upper limit of normal 95% of patients who died 53% of patients who survived			YES 2% in patients who died 38% in patients who survived			NO - 76% of fatal vs 16% of non-fatal but P >0.05		

Table S3c - Associations of mortality with comorbidities

YES = significant association (P<0.05), NO = investigated but no significant association found (P>0.05)

Author & Year	Comorbidities (all)	HIV	Asthma	Other chronic respiratory disease	Congenital heart disease	Other heart failure	Neurodevelopmental	Ex-preterm or low birth weight	Anaemia	Diarrhoea	Malaria
Adewuyi 2012		YES<br>CFR 40% with<br>CFR 9% without									
Agweyu 2015						YES – (1 of 4 deaths)				YES - complications of acute diarrhoea (1 of 4 deaths)	
Al Amad 2019	YES<br>OR 18.3										
Araya 2016	YES (CHD, Down syndrome, malnutrition and GORD)<br>OR 4.9<br><br>(HIV, cancer and severe malnutrition) OR 6.18										
Atwa 2015									NO<br>P 0.55		
Ayieko 2012											NO
Benet 2017		NO<br>P 0.10									
Boukari 2011	YES										
Caggiano 2017	YES (most common malaria, typhoid fever, anaemia)										
Cohen 2015		YES<br>CFR 7% with CFR 1% without									
Cotes 2015	YES underlying disease OR 16.5										
Daga 2014	NO	YES							YES –Hb <7g/dL		

Dembele 2019	YES - "congenital abnormalities"<br>CFR 21.4% compared with CFR 4.6% overall<br><br>OR 5.88<br>aOR 3.12								YES <8 g/dL OR 4.39 aOR 4.04), Mean 112 g/dL in patients who survived<br>Mean 103 g/dL in patients who died		
Divecha 2019				NO<br>P 0.73	YES<br>CFR 52.7% with<br>CFR 17.1% without		NO - "neurological" P 0.656		NO<br>P 0.55		
Durigon 2015	NO P 0.089										
Emukule 2014		YES<br>HIV positive OR 3.3<br>Unknown HIV status OR 2.0<br>Ref negative								YES - OR 1.8	YES<br>OR 0.3<br>aOR 0.2
Enarson 2015									YES<br>OR 1.99 aOR 1.41		YES<br>OR 0.84<br>aOR 1.07
Fagbohun 2020										YES	YES
Ferreira 2014	YES<br>OR 5.5										
Gallagher 2020		NO for HIV exposed uninfected<br>P 0.25	NO<br>P 0.23					NO for ex-preterm<br>P 0.25<br><br>No for LBW<br>P 0.53	YES<br>Hb <9.3g/dL OR 1.70<br>Hb 6 - 9.2 OR 1.44<br>Hb <6g/dL<br>OR 4.60	YES<br>OR 2.37	NO<br>P 0.87
Graham 2011		YES<br><br>ALSO:<br><br>HIV +ve TMPSMX prophylaxis CFR 3%									

		HIV +ve no prophylaxis CFR 17.5%									
Hooli 2016		CFR 6.6% in HIV infected vs 2% overall.									
Hutton 2019	NO<br>"comorbid disease excluding cardiac diagnoses"<br>P 0.48	NO<br>HIV +ve<br>P 0.79<br><br>HIV exposed uninfected<br>P 0.619			YES<br>OR 2.89<br>aOR 1.05			NO for ex-preterm<br>P 0.76.<br><br>NO for LBW<br>P 0.46			
Ibraheem 2020									YES severe anaemia CFR 35.3% with CFR 6.1% all patients		
Indriyani 2018	YES<br>75% of deaths had two or more co-morbidities (most common anaemia)										
Iroh Tam 2018		YES<br>aOR 5.0<br><br>NO for HIV exposed uninfected<br>P 0.75									
Jain 2018									YES –<br>Hb g/dL<br>Mean 8.61 in patients who died, 9.74 in patients who survived.		
Kelly 2015		YES<br>HIV infected (ref unexposed) RR 3.83<br>aRR 3.05									

		HIV exposed uninfected (ref HIV-unexposed, RR 4.78 aRR: 4.31<br><br>Mortality did not differ between HIV-exposed uninfected and HIV-infected children<br>P 0.4<br><br>HIV exposed (ref unexposed) overall RR: 6.05<br><br>Effect significant only in <6 months of age									
Kelly 2019		YES for HIV exposed uninfected						NO for low birth weight<br>P 0.37			
Kenmoe 2019		YES<br>pOR 5.51									
Kim 2019								YES for low birth weight, especially in children <2y old			
Kuti 2013						NO			NO<br>P1.0	NO<br>"gastroenteritis"<br>P1.00	NO
Lazzerini 2016		YES<br>*2-12m*<br>OR 3·93<br>*12-59m*<br>OR 3·43									YES<br>*2-12m*<br>OR 1·03 *12-59m* OR 1·52
le Roux 2015					YES - 1 of 2 deaths in infant with						

					congenital cardiac lesion						
Ma 2019		NO<br>P 0.1									NO
Macpherson 2019		YES<br>CFR 18.23% with<br>CFR 6.81% without<br>OR 3.05<br>aOR 2.31	YES<br>OR 0.11		YES - "cardiac disease"<br>CFR 20% with<br>CFR 7.78% without<br>OR 2.96		NO "chronic neurological disorder<br>CFR 5.22% with CFR 8.16% without<br>OR 0.62			YES - Diarrhoea with dehydration<br>CFR 13.46%<br><br>No diarrhoea with dehydration CFR 7.61%.<br><br>OR 1.89	NO
Matthew 2015										NO<br>P 0.7	
McCollum 2019		NO<br>P0.6							YES median Hb<br>Patients who died 8.9<br>Patients who survived 9.6<br><br>However cutoff of 6g/dL no significant difference		YES – CFR 19.3% with<br>CFR 13.7% without
Meligy 2016	NO<br>P 0.7										
Morrow 2014		YES<br>OR 3.7<br><br>In a separate model only CD4 count <15% was significantly associated with mortality OR 3.6									
Myers 2019		YES<br>100% (19 of 19) survived among HIV non exposed									

		and non-reactive<br>57% survived among HIV exposed or reactive<br>91% survived among unknown HIV status									
Nantanda 2014		NO									NO
Nimdet 2017						YES - all 3 deaths in patients with heart failure					
O'Callaghan-Gordo 2011		YES<br>OR 6.75									NO
Pale 2017		YES - both deaths in children with maternal seropositvity for HIV									
Pulsan 2019	NO<br>1.6	YES<br>OR 12.8			NO	YES<br>CFR 77.8%	YES - 2 of 2 children with Down Syndrome died.		NO<br>P 0.49		
Ramachandran 2012					YES<br>*Clinical pneumonia*<br>OR 2.7<br>aOR 1.38<br>*Radiological pneumonia*<br>OR 5.53<br>aOR 1.39<br>*Clinical & Radiological*<br>OR 3.6						
Ramakrishna 2012		YES<br>HIV negative<br>OR 0.27<br>aOR 0.19									

Reed 2012		YES<br>CFR 17.6% with<br>CFR 1.25% without						YES for ex-preterm *HIV negative patients only*<br>OR 2.2		NO	
Saha 2016	YES<br>OR 6.62<br>aOR 3.19										
Shan 2019			YES<br>OR 0.12<br>aOR 0.27		YES<br>OR 4.25<br>aOR 2.59			NO for ex-preterm			
Sonego 2015**<br><br>Systematic review – individual study data included in synthesis	YES<br>pOR 4.76<br>(12 studies)	YES<br>pOR 4.68 (14 studies)			YES<br>pOR 3.92<br>(6 studies (inc El Kholy 2013, Ramachandran 2012, Rodriguez 2014)			YES for ex-preterm<br>pOR 2.43 (6 studies)<br><br>YES for LBW<br>pOR 2.78 (9 studies)		YES<br>pOR 2.82 (6 studies)	YES - pOR 1.46 (3 studies)
Srinivasan 2012		YES<br>RR 2.7<br>aRR 2.6									
Suntarattiwong 2011	YES - 100% of deaths had chronic illness (2 x BPD, 1x CHD)			YES<br>2 of 3 deaths had BPD (both had RSV)	YES - 1 of 3 deaths						
Sutcliffe 2016		YES<br>OR 3.11 aOR 2.86<br><br>HIV exposed uninfected<br>OR 1.73<br>aOR 1.73									
Tomczyk 2019	YES<br>CFR 15% with<br>CFR 6% without<br>OR 3.0		NO	NO	YES - "Heart disease"<br>OR 4.5			NO for ex-preterm			
Tuti 2017	YES<br>OR 1.91										NO<br>P 0.36

Walk 2016	YES - severe pneumonia with co-morbidity<br>OR 3.52<br><br>Multi-organ failure OR 12.67	YES<br>OR 4.35			NO						
Webb 2012		YES – "HIV or malnutrition" only at 5 days (i.e not at 48 hours) in those with severe pneumonia									
Wilson 2017									NO<br>Median Hb 8.2 in patients who died<br>8.3 in patients who survived<br>P 0.97		YES<br>CFR 3% with<br>CFR 4% without<br>P 0.047
Zabihullah 2017									NO		
Zampoli 2011		YES<br>CFR 35% with<br>CFR 11% without<br>OR 4.5									
Zhang 2013					YES<br>OR 6.28		YES<br>Down syndrome<br>OR 6.82<br>Cerebral Palsy<br>OR 8.69,		YES<br>Hb <8g/dL<br>61% of patients who died<br>8% of patients who survived		
Zhang 2011	YES - 60% (3 out of 5) of patients who died (all 3 CHD)<br>14% of patients who survived				YES - 3 out of 5 deaths had congenital heart disease (2 of whom had anaemia), vs 10				YES<br>2 of 5 patients who died (both also had		

					out of 816 survivors				congenital heart disease) 36 out of 816 patients who survived		
Zurita-Cruz 2020	YES “pathological history”<br>CFR 2.04% with<br>CFR 0.38% without			YES - BPD OR 1.69	YES - OR 3.4			NO<br>Ex-preterm<br>P 0.409			

Table S3d - Associations of mortality with markers of malnutrition

YES = significant association (P<0.05), NO = investigated but no significant association found (P>0.05)

Author & Year	Weight for age Z-score (WAZ) –2 to -3 SD (or where specified <-2)	Weight for age Z-score (WAZ) <-3 SD	Weight for height Z-score (WHZ) -2 to -3 SD (or where specified <-2)	Weight for height Z-score (WHZ) <-3 SD	Height for age Z-score -2 to -3 SD (or where specified <-2)	Height for age Z score <-3 SD	Malnutrition otherwise (or not) specified
Agweyu 2018	YES<br>CFR 7.6%<br>CFR 3.8% of WAZ > -2<br>RR 2.0 aRR 2.1	YES<br>CFR 11.2%<br>ref CFR 3.8% for children WAZ >-2<br>RR 2.9					
Agweyu 2018	NO - WAZ <-2<br>P 0.14						
Bekele 2017							YES
Benet 2017			YES - Median SD<br>−2.1 in patients who died<br>−1.1 in patients who survived	NO<br>P 0.06			
Bokade 2015		YES<br>CFR 22.5<br>aOR 15.51					
Boukari 2011							YES
Caggiano 2017							NO
Chisti 2010							YES – “severe malnutrition”<br>CFR 22.6% with<br>CFR 0% (0/17) without.<br>RR 1.71
Daga 2014							YES
Dembele 2019	YES<br>OR 1.41<br>aOR 1.58	YES<br>CFR 8.9%<br>OR 2.93<br>aOR 3.22			NO<br>P 0.32	NO P 0.32	
Emukule 2014	YES<br>OR 2.3<br>aOR 2.1	YES<br>OR 4.7<br>aOR 3.8	YES<br>OR 1.7	YES<br>OR 4.2	NO<br>P 0.34	YES - OR 2.5	
Enarson 2015							YES (severe wasting or nutritional oedema)<br>OR 2.83 aOR 2.37
Ezeonu 2015							YES - "inadequate nutrition"<br>CFR 20%
Fagbohun 2020							YES - "malnutrition”

Ferreira 2014							YES<br>OR 4.1
Gallagher 2020	YES<br>OR 2.39	YES<br>OR 4.43	YES<br>OR 2.54<br>aOR 2.45	YES<br>OR 4.55<br>aOR 3.57<br>(ref >-2 SD)	NO<br>OR 1.28 (0.72-2.28)	YES<br>OR 1.72<br>(1.04-2.85)<br>(ref >-2 SD)	
Hasan 2014	NO – WAZ <-2<br>P 0.36						
Hooli 2016							YES<br>*MUAC 11.5-13-5*<br>OR 1.73<br>*MUAC <11.5*<br>OR 4.63
Hutton 2019			NO<br>P 0.1	YES<br>OR 4.99 aOR<br>8.25			
Ibraheem 2020							YES<br>Protein energy malnutrition CFR 28.6%<br>All patients CFR 6.1%
Iroh Tam 2018							YES<br>Low MUAC aOR 0.96
Jain 2018				YES<br>CFR 25%<br>OR 7.46 aOR<br>8.02			
Kelly 2019							YES
Ku 2020							YES - "patients with all forms of malnutrition (mild, moderate, and severe). All cases of Kwashiorkor were considered severe malnutrition."<br>CFR with 8.7%<br>CFR without 3.7%
Kuti 2013	NO WAZ < -2SD<br>P 0.5			YES - 8 of 15 (53.3%) of patients who died<br>69 of 374 (18.4%) of patients who survived<br>aOR 5.052	NO<br>HAZ < -2SD<br>P 1.0		
Lazzerini 2016	YES	YES		YES			

	2-11m<br>OR 2·60 aOR 2·27<br>*12-59m*<br>OR 2·07 aOR 1·70	*2-12m*<br>OR 4·24<br>aOR 3·42<br>*12-59m*<br>OR 5·00<br>aOR 3·23		*2-12m*<br>OR 6·62 aOR<br>2·85<br>*12-59m*<br>OR 10·04 aOR<br>4·19<br>CFR 34·8%			
Lufesi 2015							YES - WHO severe undernutrition<br>CFR 15.4%<br>All pts CFR 6.6%
Ma 2019		NO<br>P 0.22					
Macpherson 2019		YES<br>aOR 2.99					YES<br>MUAC <115mm CFR 17.02%<br>MUAC >115mm CFR 5.11%<br>OR 3.81<br><br>"severe malnutrition" CFR 16.95%<br>Absence of severe malnutrition CFR 6.98%<br>OR 2.72 aOR 1.89
McCollum 2019							YES<br>MUAC mean 11.9 in patients who died<br>MUAC mean 12.8 in patients who survived.<br><br>Severe acute malnutrition without HIV infection or exposure CFR 20.7%
Moschovis 2015					NO HAZ <-2 SD<br>RR 2.32 (95% CI 0.99, 5.45)		
Naheed 2019		YES<br>OR 3.25 aOR<br>1.79					
Nantanda 2014			YES - WHZ < -2<br>HR 6.4 aHR 5.7				
Negash 2019		YES<br>OR 13.5					
Olofin 2013**<br><br>Systematic review – individual study data included in synthesis				YES<br>HR 9.68			
Onyango 2012		YES – WAZ <-4					

		3 of 4 influenza deaths					
Pulsan 2019							NO<br>4 out of 5 “severe malnutrition” died<br>OR 3.6 but did not reach significance
Ramachandran 2012	NO - WAZ -2 to -3 not significant<br><br>HOWEVER - WAZ <-2<br>*Clinically diagnosed pneumonia*<br>OR 1.8<br>aOR 1.12<br><br>*Clinically &radiologically diagnosed pneumonia*<br>OR 2<br>aOR 1.13	YES<br>OR 2.25					
Ramakrishna 2012							NO
Reed 2012	YES only in *HIV negative patients* OR 4.8 aOR 2.5	YES<br>*HIV -ve*<br>OR 14.5 aOR 6.0<br>*HIV +ve*<br>OR 1.5	YES *only in HIV negative patients*<br>OR 3.5	YES<br>*HIV -ve*<br>OR 7.9<br>*HIV +ve*<br>OR 1.3	YES<br>In HIV negative patients only<br>OR 2.7	YES<br>In HIV negative patients only<br>OR 2.3	
Saha 2016	YES "low weight for age"<br>OR 6.3 aOR 4.77						
Sonego 2015**<br><br>Systematic review – individual study data included in synthesis							YES<br>Severe vs no malnutrition pOR 4.27 (21 studies).<br>Moderate vs no malnutrition pOR 2.46 (18 studies)
Sutcliffe 2016			YES<br>OR 1.72<br>aOR 1.57	YES<br>OR 2.23 aOR 1.85			
Tomczyk 2019	YES - WAZ <-2<br>CFR 86% with<br>CFR 40% without<br>OR 9 aOR 7						
Tuti 2017	YES<br>OR 2.08	YES<br>OR 3.66					
Webb 2012							NO – not significant severe malnutrition with negative HIV test

							HOWEVER In those with very severe pneumonia at 5 days, those with HIV or severe malnutrition vs those without had a significant increase in mortality.
Zabihullah 2017	YES - WAZ <-2<br>CFR 20.6% with<br>CFR 10% without<br>OR 2.06						

Table S3e - Associations of mortality with aetiology and laboratory findings

YES = significant association (P<0.05), NO = investigated but no significant association found (P>0.05)

Author & Year	Bacterial	Viral	PjP	Leucocytes	Platelets	CRP	Procalcitonin	Lactate	Other laboratory findings
Acuna 2018	YES<br>Bacteraemia RR 2.88<br>Staphylococcus RR 2.23			YES Leukopenia (< 4500/mm3)<br>RR 2.34					
Adewuyi 2012		NO – though trend towards higher CFR in CMV (20% of positive, 12% of negative)							
Al Amad 2019		YES<br>Influenza B OR 6.8							
Ali 2013		YES<br>H1N1 Influenza CFR 14.8%<br>OR 12.9							
Araya 2016	NO	NO		YES - Leucopenia (<4000/mm3)<br>OR 6.5<br><br>Leucocytosis (>15,000/mm3)<br>OR 0.15					pH<7.2 OR 77<br>HCO3<15<br>OR 26.7
Atwa 2015				YES - Leucopenia<br>OR 2.119<br><br>Leucocytosis non-significant P 0.79	YES – Low platelets<br>OR 1.92<br><br>Thrombocytosis non-significant<br>P 0.18	NO P 0.84			
Awasthi 2018									YES<br>Higher serum levels of Interleukin-1 receptor antagonist (IL-1RA)
Barger-Kamate 2016	NO<br>Pertussis CFR 23.1%<br>P 0.43								
Benet 2017	YES – Blood PCR positive<br>HR 4.6 aHR 4.0	YES<br>Parainfluenza nasal swab/aspirate<br>HR 23.6					YES - ng/mL, median 71.5 in patients who died, 1.6 in		

	S. pneumoniae in respiratory samples - not significant.<br>No difference among S. pneumoniae serotypes						patients who survived<br><br>≥ 50 ng/mL HR 22.4		
Berkley 2010		YES - Patients with RSV were “less likely” to die. Of the 2 deaths among children with RSV, both had congenital heart disease.<br>Other viruses – no association							
Bokade 2015				YES - total white count > 20000/mm2 CFR 19.2%					YES - hypoglycaemia
Caggiano 2017	NO								
Champatiray 2017	NO - "bacteriology" P0.22								
Cohen 2015	YES for pneumococcus	YES - lower case fatality in RSV							
Daga 2014									YES pH less than 7.3
Dembele 2019	YES<br>CFR 16.7%<br>OR 7.02	YES<br>Human metapneumovirus<br>OR 0.25<br>RSV OR 0.33<br>Adenovirus<br>OR 4.18<br>H1N1 Influenza<br>OR 14.09<br><br>Otherwise no association with virus positivity overall, influenza overall, rhinovirus, enterovirus, parainfluenza		YES<br>Mean WCC in patients who died died 13.1<br>Mean WCC in patients who survived 12.2<br><br>Mean neutrophil count in patients who died 9.86<br>Mean neutrophil count in patients who survived 7.17	NO<br>P 0.85				
Diez-Padrisa 2010	YES<br>CFR 24% bacterial<br>CFR 0% (0/87) viral	YES<br>CFR 24% bacterial<br>CFR 0% (0/87) viral				YES<br>Mean 161.3 mg/l in patients who died<br>Mean 60.95mg/l	YES<br>Median 19.75 ng/ml in patients who died<br>Median 1.08 ng/ml in patients who survived		

						in patients who survived			
Divecha 2019				YES – leukocytosis or leukopaenia<br>CFR 43.5%<br>CFR 17% for all patients					
Do 2011		One of two fatal cases was diagnosed with influenza A (H5N1) (of 2 total patients with H5N1)							
Durigon 2015		Six of the nine infants (66%, relative to 53% of patients overall) that died had a respiratory virus detected, distributed as follows: PIV 1 (n=2); hMPV (n=1); RSV (n=1); ADV (n=1) and one coinfection ADV+RSV. All six had an underlying condition							
Feikin 2017		YES<br>Influenza A was the only virus with higher mean viral load in fatal compared with surviving cases							
Fischer Langley 2013		NO<br>RSV P 0.68							
Gallagher 2020				NO P0.42		NO CRP > 40mg/L<br>P 0.11			
Graham 2011	NO		YES<br>CFR 73%<br>Bacterial CFR 4%						
Hasan 2014	YES<br>CFR bacterial 3.5%<br>CFR viral 0.1%	YES<br>CFR bacterial 3.5%<br>CFR viral 0.1%							
Hatem 2019		NO<br>RSV - P 0.474<br>Adenovirus P 0.963<br>Influenza P 0.202							

Hutton 2019	YES - (from blood culture, bronchoalveolar lavage, sputum or histology)<br>OR 4.01 (1.53 to 10.57)<br>aOR 3.61 (0.65 to 20.00)	NO							
Indriyani 2019									YES – ESR<br>Every increase in one log ESR decreased log odds of death about 3.3
Jain 2018				NO P 0.051	YES - (lac/mm3)<br>Mean 0.82 in patietns who died<br>Mean 2.98 in patients who survived,<br><br>(<0.7 lac/mm3)<br>OR 10.71				
Jroundi 2014	YES								
Julien 2020		One of 3 deaths in which nasopharyngeal washing was collected positive for co-infection by B. pertussis, parainfluenza virus, and influenza virus							
Kelly 2015		YES<br>RSV RR: 0.09 ref. other viruses (though “treatment failure” risk significantly higher than non-RSV)<br>Did not significantly differ from viral negative							
Kim 2019				YES<br>Neutrophil count ≥10x10.9/L					
Kuti 2013	NO – bacteraemia<br>P1.0								
Lanaspa 2015			YES						

			significantly higher						
Lazzerini 2016			YES<br>*2-12m*<br>OR 19·93<br>aOR 21·35<br>*12-59m*<br>OR 5·26<br>aOR 6·25						
le Roux 2015	YES - 1 of 2 deaths in infant with pneumococcal sepsis and empyema								
Ma 2019								YES<br>Median 7.2mmol/L in patients who died<br>Median 2.4mmol/L in patients who survived<br><br>Lactate <2<br>0% of patients who died<br>9.8% of patients who survived<br>Lactate 2-4mmol/l 45% of patients who died<br>70% of patients who lived<br>Lactate >4<br>55% of patients who died<br>20% of patients who survived<br><br>32% with slow lactate	YES - glucose <3 27% of patients who 4.5% of patients who survived.

								clearance<br>died<br>11% of patients with<br>efficient lactate clearance died	
Matthew 2015	NO<br>25 deaths, organisms distributed in a pattern similar to the 428 children who surived. 76% deaths among children with NPA PCR results were pneumococcus, compared with 77% of all children with positive results	NO							
Meligy 2016		NO for any virus including RV, Parainfluenza, Boca, Corona, H1N1, enterovirus,							
Mohamed 2017									YES<br>Copeptin<br>Mean 1811.8 in patients who died<br>745.4 in patients who survived
Morrow 2014			YES<br>CFR 32.1% with<br>CFR 17.2% without<br>RR 1.87 (on multivariate analysis only HIV infection predictive)						
Naheed 2019	YES<br>OR 2.7<br>aOR 1.94								
Negash 2019	NO<br>P0.12								

	Organisms isolated from children who died were S. aureus, K. pneumoniae, and Kocuria kristinae								
O'Callaghan-Gordo 2011		Viruses found among 19 fatalities were listed as follows: 8 RV, 2 ADV, 2 RSV, 2hMPV, 1 Flu, and 4 dual detections (3 RV and ADV; 1 RV and hMPV)							
Olsen 2010	NO	NO							
Pulsan 2019		NO							
Rajatonirina 2013	NO	NO including RSV and Influenza							
Ramachandran 2012		NO for measles							
Ramakrishna 2012	NO							YES<br>Median lactate in patients who died 4.2<br>Median lactate in patients who survived 2.6<br><br>92% of patients who died had lactate >2 mmol/l, ref. 61% of patients who survived<br><br>Lactate >2.0 mmol/l OR 7.48<br>aOR 4.68	
Reed 2012						NO - CRP >40			
Saghafian-Hedengren 2017									YES<br>IL-1RA, IL-6, IL-8, IL-17, MIP-1α higher in non-survivors

									CCL22 lower in non-survivors
Saleh 2018									YES<br>Serum Zinc 29.9 in non-survivors, 50.3 in survivors
Shan 2019				YES Leukocytosis<br>OR 1.77 aOR 1.68		YES CRP<br>>8mg/l<br>OR 1.44,<br>aOR 1.49			
Sonego 2015**<br><br>Systematic review – individual study data included in synthesis	NO	YES<br>RSV pOR 0.46 (6 studies)	YES<br>pOR 4.79 (5 studies)						
Srinivasan 2012	NO								
Sudarwati 2014	YES<br>All 6 deaths had CONS, 2 of which also had viral infection (coxackie and rhinovirus)	2/6 deaths virus positive<br>- 1 rhino, one coxackie, RSV, adenovirus							
Suntarattiwong 2011		NO for influenza and RSV<br>P0.34							
Suzuki 2012	YES – positive blood culture<br>CFR 16.1% with<br>CFR 10.6% without<br>3.8% blood cultures were positive<br>Organisms isolated from children who died were:<br>3 x B. cepacia (out of 9 overall) and 2 x MRSA (out of 4 S. aureus of which 2 were MRSA)	YES - Flu A OR 4.3<br>Cases with at least one virus less likely to be fatal than virus-negative cases (8.6% vs 13.9%), borderline signficance OR 0.62 (0.5-1)<br>NO – RSV, Adenovirus, hMPV & HRV							
Tomczyk 2019	NO	YES<br>Any virus OR 0.5<br>Parainfluenza OR 1.9<br>Human metapneumovirus OR 0.5<br>RSV OR 0.6 aOR: 0.5							

		Influenza A OR 1.8<br><br>NO – Adenovirus, Influenza B							
Zhang 2013	NO	NO		NO		NO		YES >382 U/l in 73% of fatal vs 54% of non-fatal cases	YES<br>Glucose >6.2mmol/l or <2.5mmol/l in 95% of fatal, 56% of non-fatal cases<br>CK-MB fraction >26 U/l in 63% fatal, 45% non-fatal cases.<br><br>CK, Creatinine, AST, ALT all non-significant
Zhang 2020									YES<br>Angiopoietin-1 lower in fatal cases (8.5 vs 18 ng/ml)<br>Angiopoietin-2 /angiopoietin -1 ratio higher in fatal cases (0.36 vs 0.077)<br>(Angiopoietin -2:angiopoietin-1 also found to be higher in pneumococcal vs viral pneumonia)
Zidan 2014									NO for serum IL-6

Table S3f - Other investigated factors for associations with mortality

Author & Year	Chest X-Ray Consolidation	Other Chest X-Ray findings	Other investigated factors
Agweyu 2018			YES - area of high malaria prevalence RR 1.3
Agweyu 2018			YES - Provincial vs District Hospital OR 2.7, aOR 2.2
Al Amad 2019			YES – Study site OR 5.4
Araya 2016	YES - Multilobar consolidation OR 4.9<br><br>Patchy consolidation OR 0.1<br><br>Unilobar consolidation non significant P 0.18	YES<br>Pneumothorax OR 15<br>Pleural effusion OR 2.6	YES - Hypotension OR 48.7, Seizures OR 10.6, GCS <13 OR 324, Hepatic impairment OR 19, Renal impairment OR 15.2,
Awasthi 2018			YES - IL1RA gene genotype A1/A1 OR 0.05, A2/A2 OR 58. A1 (vs other alleles) OR 0.06, A2 (vs other alleles) OR 21.72. A3, A4 not sig.
Ayieko 2012			YES - case fatality rates varied significantly between hospitals (LR $\chi 2 = 52.19$; $p<0.001$), ranging from 3.1% (95%CI, 0.9 to 5.4) in H7 to 13.2% (95%CI, 6.9 to 19.5) in H5
Azab 2016			YES - Interleukin-10 -1082, GG genotype or G allele associated with higher mortality, AA genotype or A allele associated with lower mortality
Bekele 2017			YES - smoker in the house, previous pneumonia,
Benet 2017			YES - inabilty to drink HR 2.7
Bills 2020			YES<br>Only child, Smoker in home, Open cook stove OR 2.44 aOR 2.40 (1.36 - 4.24), urban vs rural OR 2.74 aOR 1.84, Lower level facility OR 3.67 aOR 1.67, abnormal mental status OR 7.39 aOR 2.14
Bokade 2015			YES<br>Residence in urban slum, Grunting, altered consciousness, refusal to feed, shock
Champatiray 2017			NO - exposure to smoke P 0.36
Daga 2014			YES - Altered conscious level, convulsions
Dembele 2019	YES<br>OR 4.23 aOR 4.11	YES<br>Overall CXR positive CFR 6.0%<br>Overall CXR negative CFR 2.6%<br><br>Endpoint pneumonia<br>OR 2.43 aOR 2.67<br>Infiltrates<br>OR 1.52 aOR 1.71<br>Pleural effusion<br>OR 6.96 aOR 6.85	YES – Which hospital admitted in, CFR range from 2.1 to 5.7%<br>Past experience of child loss OR 4.9 aOR 5.42<br>Decreased breath sounds OR 3.2 aOR 5.36<br>Grunting OR 16.72 aOR 14.27<br>Sensorial changes OR 9.61 aOR 8.31<br>GCS <9 OR 45.0<br><2 siblings CFR 1.5%, 2-3 siblings CFR 1.8%, 4 or more siblings CFR 4.4%<br>neck rigidity CFR 25%<br>Apnoeic episode<br>Nasal flaring

			NO - Type of cooking fuel used P0.78
Divecha 2019			YES - PRISM score P<0.001, shock P <0.001 sepsis P <0.001, electrolyte abnormality P 0.005<br>NO - TB or past TB P 0.46, TB contact P0.39
Emukule 2014			YES - Enrolled in clinical research studies OR 0.2<br>Diagnosed TB OR 3.3<br>Previous hospitalisation with same illness OR 4.9<br>Unconscious OR 3.1 aOR 2.3<br>Lethargic OR 2.1<br>Unable to drink OR 3.2 aOR 1.8 (only significant in <2y olds)<br>Night sweats OR 0.4 aOR 0.5<br>Nasal flaring OR 3.4<br>VP or U on AVPU scale OR 13.5 aOR 8.0
Enarson 2015			YES - Meningitis OR 4.82 aOR 2.49<br>NO - sepsis
Enarson 2014			NO - region of country (south, central or north)
Fagbohun 2020			YES - Convulsions, confusion, coma, inability to feed
Fancourt 2017	YES<br>CFR 13.5%	YES<br><br>Infiltrates only<br>26.7% of patients who died<br>52.0% of patients who lived	
Ferreira 2014			
Gallagher 2020		YES<br>"abnormal"<br>OR 1.95<br>"uninterpretable"<br>OR 2.56	YES - Study site – CFR 26% Zambia, 1.5% Bangladesh, others in between<br>ORs (ref. South Africa 1.0) 0.47 (Bagladesh) to 10.6 (Zambia). aORs 0.77 (Thailand) to 12.3 (Zambia) - ONLY significant for Zambia on aOR<br><br>YES – Responds only to voice OR 6.06, Responds only to Pain OR 6.28, Unresponsive OR 17.6. Any decreased responsiveness OR 7.10, Grunting OR 2.77 aOR 2.48, Inability to feed OR 4.63, CRT 2-3s OR 2.61, CRT >3s OR 5.42<br><br>NO - Deep breathing, head nodding, vomiting P0.46, Convulsions, nasal flaring P 0.62, ear discharge P0.95, Crowding at home P 0.95, Solid fuel use P0.07, Smoke exposure at home P0.23.
Gowraiah 2014			YES - unresponsive or only responds to pain, sensitivity / specificity 87.5/94.2
Hasan 2014		YES - radiographically confirmed pneumonia<br>CFR 0.8% with<br>CFR 0.2% without	
Hooli 2016			YES - unconscious OR 5.68
Hutton 2019			YES - Duration in hospital (days) median in patients who died 8.35, median in patients who survived 12.9<br>NO - smoke exposure at home
Ibraheem 2020		YES<br>Pleural effusion CFR 30.3%<br>Pneumothorax CFR 20%.	YES - duration of hospital stay<br>< 4 days CFR 8.6%<br>>4 days CFR 2.5%

		All patients CFR 6.1%	
Jain 2018			YES - Abnormal sensorium OR 19.87 (aOR 21.78), Any Complications (one or more of: pyogenic meningitis, blood culture positive bacteremia, pleural effusion, acute respiratory distress syndrome, pyopericardium or septic arthritis) OR 3.68
Kim 2019	YES		
Kuti 2013	NO<br>P 0.19	NO<br>P1.0<br>Cardiomegaly on CXR<br>P1.0	YES – Grunting, convulsions (aOR 16.6), somnolence, lethargy, meningitis<br>NO - Head nodding, inability to feed, kwashiorkor and marasmic kwashiorkor (P0.3)
Laman 2013			YES - Head nodding, drowsiness
Lazzerini 2016			YES<br>Previous hospital admission for pneumonia 2-12m OR 1·17, 12-59m OR 0·83.<br>Wet season (vs dry) 2-12m OR 0·90, 12-59m OR 1·08 aOR 1·57
Ma 2019	NO<br>P 0.57	NO<br>P 0.57	YES – Lethargy, altered level of consciousness<br>NO - convulsions P 0.57; vomiting P 0.77, unable to feed/drink P 0.17. Nasal flaring P>0.99
Macpherson 2019			YES - High malaria transmission region OR 1.71<br>Acute neurological disorder OR 6.29<br>Sepsis OR 5.89<br>Convulsions OR 2.48<br>Grunting OR 2.15<br>Acidotic breathing OR 2.21<br>Reduced consciousness OR 6.65 aOR 3.65<br>Cannot eat or drink OR 3.42 aOR 1.82<br>NO - Readmitted P 0.06, Sickle cell disease, Renal disease, Diabetes
Matthew 2015	YES		
McCollum 2019			YES - grunting, apnoea, inability to feed, vomiting everything, convulsion, Blantyre coma score <4<br>NO - head nodding or tracheal tug, nasal flaring
Naheed 2019			YES - Delay in presentation from illness onset OR 1.14 (1.04 - 1.12)
O'Callaghan-Gordo 2011			NO Season (rainy vs dry)
Pagano 2018			YES - Among malnourished children Near Infrared Spectroscopy (NIRS) testing of peripheral oxygen tissue saturation index (TSI) - TSI decline after 40s of the arterial occlusion provided the best NIRS related prediction of mortality from pneumonia.
Pulsan 2019			YES - septic shock - 100% (8/8) died.<br>100% (2/2) meningitis died.
Ramachandran 2012		YES<br>CFR 12.4% with both clinical and radiological diagnosis<br>CFR 5.6% in only clinical or only radiological diagnosis	YES<br>Altered consciousness OR 5.1 aOR 1.56 in clincally diagnosed, OR 14.5 (aOR 1.56) in radiologically diagnosed, OR 4.69 in clinically and radiologically diagnosed<br><br>Convulsions OR 11.15 in radiologically diagnosed, non signfiicant in clinically or clinically &radiologically diagnosied.<br><br>Shock OR 2.3 in clinically diagnosed, non significant in radiologically or clinically &radiologically diagnosed

Ramakrishna 2012		YES - evidence of interstitial infiltrate and/or hyperinflation vs consolidation OR 5.0	YES<br>Capillary refill time >2s OR 7.26 (aOR 2.89 non sig)
Reed 2012	YES – Only in HIV negative patients<br>OR 4.5	YES<br>OR 0.6 in HIV positive patients only	
Saha 2016			YES<br>Unsafe water OR 2.49, aOR 2.17<br>Non hygeinic latrine not significant<br>Number of family members in household not signficant
Shan 2019		YES<br>OR 0.65 aOR 0.75	
Sonego 2015**<br><br>Systematic review – individual study data included in synthesis			YES<br>Disease in pregnancy OR 2.62 in one study<br>Maternal TB OR 4.36 in one study<br>Lack of sewage/latrine pOR 1.82 (3 studies)<br>Lower quality drinking water pOR 2.85 (3 studies)<br>Crowding pOR non signficant (9 studies)<br>Second hand smoke exposure pOR 1.52 (8 studies)<br>Indoor pollution pOR 3.02 (6 studies)<br>Rural vs urban residence pOR non significant<br>Antenatal care and birth spacing vs no: pOR 0.5 (3 studies)<br>Maternal child card present OR 0.02 (1 study)<br>Child ever visited welfare clinic OR 0.13 1 study<br>Lack of identification of ALRI by caregivers OR 2.13 1 study<br>Non-institutional barriers to care OR 3.12 1 study<br>Late referral by caretakers OR 20 1 study<br>Late referral from primary care to hospital OR 7.56 1 study.
Sutcliffe 2016			YES - "pneumonia season" OR 0.61 aOR 0.85 not significant
Tomczyk 2019	YES<br>Lobar consolidation and/or effusion<br>OR 2.6 aOR 2.5		NO - High crowding index not significant
Tuti 2017			YES - Hospital in malaria endemic area OR 1.3
Webb 2012			YES<br>Signs of shock on admission OR 9.99<br>History of convulsions OR 6.83,
Wilson 2017			NO - Blood pressure no sig difference
Zeeshan 2020			YES - PRISM III score >10
Zhu 2012			YES<br>ARDS RR 9.7
Zidan 2014			YES

			CFR 30.8% in patients with IL6-174 CC genotype<br>CFR 5.4% in patients with CG genotype<br>CFR 0% (0 out of 32) in GG genotype
Zurita-Cruz 2020			YES<br>Clinical pneumonia diagnosis (ref. bronchiolitis diagnosis OR 4.25<br>In hospital complications OR 7.87<br>Invasive medical procedures OR 11.17

Table S3g – Associations of treatment failure with demographic features

YES = significant association (P<0.05), NO = investigated but no significant association found (P>0.05)

Author & Year	Child's age (months)	Female sex	Breastfeeding status	Immunisation Status	Maternal Age	Parental Education	Socio-economic status	Other demographic factors
Agweyu 2015				NO				
Basnet 2015	YES<br>2-6 m<br>OR 1<br>7-11 months Non sig<br>P 0.06<br>12-23m<br>OR 0.37<br>24-35m<br>OR 0.22<br>P 0.049	NO	NO<br>P 0.37					
Jakhar 2018	NO<br>P 0.1	NO	NO<br>P 0.47					
Kelly 2019			NO<br>P 0.38					
King 2015	NO			NO (pentavalent or PCV 13)				
Moschovis 2015	NO P 0.53	YES<br>aRR 1.25	NO P 0.67	YES - Immunized appropriately RR 0.74				
Moschovis 2013	NO	YES<br>Male sex aOR 0.48 at high altitude, not sig at low altitude	YES<br>Breastfed per WHO standards- high altitude<br>OR 2.34, not sig at low altitude	YES - Immunized appropriately - low altitude OR 0.45, non sig at high altitude				
Nguyen 2019	YES<br>12-23m (ref 2-11m)<br>OR 0.6<br>aOR 0.7<br>24-59m OR 0.3<br>aOR 0.6	YES<br>Male sex<br>OR 1.3<br>aOR 1.3	YES<br>OR for any breastfeeding 0.6<br>aOR 0.8	NO				
Rajatonirina 2013	YES	NO					YES	

	aOR 5.3 for 0-5months, non sig for other age groups.						Monthly income <$182<br>OR 4.4<br>aOR 4.1<br>Monthly income $182-455 OR 3.5<br>aOR 4.3	
Tapisiz 2011	NO	YES<br>Male sex<br>OR 1.82<br>aOR 4.31						
Walk 2016	NO	NO						
Webb 2012	YES<br>OR 1.66							
Zhang 2013	NO	NO						

Table S3h - Associations of treatment failure with clinical features

YES = significant association (P<0.05), NO = investigated but no significant association found (P>0.05)

**Author & Year**	**WHO defined severe pneumonia**	**Hypoxaemia (SaO2 <90% except where specified)**	**Cyanosis**	**Tachypnoea (per WHO definition except where specified)**	**Chest indrawing**	**Cough**	**Wheeze**	**Other auscultation findings**	**Tachycardia (per WHO definition except where specified)**	**Pallor**	**Fever (temperature ≥38°C except where specified)**
Agweyu 2015							YES<br>OR 0.18<br>aOR 0.21				
Basnet 2015		YES<br>OR 1.91									No for >38.5<br>P0.8
Gowraiah 2014				YES					YES (>166/min)		
Jakhar 2018		NO<br>P 0.07		YES for RR >10 above upper threshold of normal<br>aOR 19.94				YES - Crepitations			
Kelly 2019	YES<br>P <.0001										
King 2015		NO for SaO2 <95%									NO
Moschovis 2015		YES<br><br>Per each 10% increase in SaO2<br>aRR 0.71									
Moschovis 2013		YES<br><br>Per each 10% increase in SpO2<br>High altitude OR 0.69<br>Low altitude OR 0.74							NO		
Nguyen 2019	YES<br>OR 10<br>aOR 7.4										
Tapisiz 2011				YES – average RR 50 in success group, 58 in treatment failure group							NO<br>P 0.43

Wandeler 2015		YES<br>P = 0.006									
Webb 2012	YES<br>48 hours<br>OR 1.93<br>Day 5<br>OR 2.93	YES – SaO2 <95<br>48 hours<br>OR 1.78<br>5 days<br>OR 2.88									
Zhang 2013		YES - 75% of patients with treatment failure vs 37% without	YES - 72% of patients with treatment failure vs 32% without	YES for RR >10 above upper threshold of normal<br>- 95% of patients with treatment failure vs 45% without			YES - 5% of patients with treatment failure vs 45% without			NO	

Table S3i - Associations of treatment failure with comorbidities

YES = significant association (P<0.05), NO = investigated but no significant association found (P>0.05)

Author & Year	Comorbidities (all)	HIV	Asthma	Other chronic respiratory disease	Congenital heart disease	Other heart failure	Neurodevelopmental	Ex-preterm or low birth weight (LBW)	Anaemia	Diarrhoea	Malaria
Agweyu 2015										YES<br>OR 2.4	NO
Jakhar 2018									NO<br>Mean Hb in failed treatment 9.40 ± 1.96<br>P 0.230		
Kelly 2015		YES<br>RR 2.55<br>aRR: 2.08									
Kelly 2019								YES for LBW			
King 2015											YES<br>OR 1.62
Moschovis 2013									YES<br><br>Not significant on univariate analysis<br><br>At high altitude multivariate analysis<br>aRR: 4.07<br><br>At low altitude non significant		
Nguyen 2019								YES for LBW<br>OR 2.4<br>aOR 1.8			
Rajatonirina 2013	YES<br>OR 3.9<br>aOR 4.6										
Tapisiz 2011	NO - "underlying disease" no signficance										
Walk 2016	YES - very severe pneumonia with vs	YES<br>OR 4.35			NO						

	without co-morbidity OR 3.52.<br>Multi-organ failure OR 12.67										
Webb 2012		YES<br><br>Positive HIV vs negative HIV, in non-malnourished patients<br>48 hours<br>OR 3.45<br>5 days<br>Non significant<br><br>Positive HIV with severe malnutrition non significant									
Zhang 2013									YES<br>Hb <8g/dL in 42% of failure vs 3% of non-failure		

Table S3j - Associations of treatment failure with markers of malnutrition

YES = significant association (P<0.05), NO = investigated but no significant association found (P>0.05)

Author & Year	Weight for age Z-score (WAZ) –2 to -3 SD (or where specified <-2)	Weight for age Z-score (WAZ) <-3 SD	Weight for height Z-score (WHZ) -2 to -3 SD (or where specified <-2)	Weight for height Z-score (WHZ) <-3 SD	Height for age Z-score -2 to -3 SD (or where specified <-2)	Height for age Z score <-3 SD	Malnutrition otherwise (or not) specified
Agweyu 2015			YES<br>OR 2.91				
Ahmed 2016							YES<br>Severe malnutrition CFR 5%<br>Moderate Malnutrition CFR 2.52%<br>No Malnutrition CFR 1%
Basnet 2015			YES<br><-2 WHZ<br>OR 1.49		NO for <-2 HAZ<br>P0.46		
Gowraiah 2014		YES					
Jakhar 2018				YES			
Kelly 2019							NO<br>P0.09
King 2015							YES<br>MUAC <13.5cm OR 1.88
Moschovis 2015					YES<br><-2 HAZ<br>RR 1.24<br>aRR 1.28<br><br>For every standard deviation increase in height-for-age, risk of treatment failure decreased by 7% (RR = 0.93)<br><br>Treatment failure by 5 days aRR 1.62		
Moschovis 2013	YES<br>Per point increase in WAZ<br>OR 0.80 at high altitude<br>OR 0.81 at low altitude						

Olofin 2013**<br><br>Systematic review – individual study data included in synthesis	YES<br>WAZ -1 to -2<br>HR 1.85<br>WAZ -2 to -3<br>HR 3.11	YES<br>HR 10.10<br>(ref WAZ > -1)	YES<br>WHZ -1 to -2<br>HR 1.92<br>WHZ -2 to -3<br>HR 4.66		YES- HAZ -3 to -2 HR 2.18<br>WHZ -2 to -1 HR 1.55 (ref >-1)	YES – HAZ <-3<br>HR 6.39 (ref >-1)	
Webb 2012							YES<br>In HIV negative patients<br>OR 1.94 at 48 hours<br>Non significant at 5 days.

Table S3k - Associations of treatment failure with aetiology and laboratory findings

YES = significant association (P<0.05), NO = investigated but no significant association found (P>0.05)

Author & Year	Bacterial	Viral	PJP	Leucocytes	Platelets	CRP	Procalcitonin	Lactate	Other laboratory findings
Basnet 2015		NO - Influenza A, Influenza B, Parainfluenza PIV1, Parainfluenza PIV2, Parainfluenza PIV3, RSV							
Jakhar 2018	YES – for positive nasopharyngeal aspirate culture and positive blood culture<br>aOR 15.24 (2.53–91.67)			NO for total lymphocyte count (>11,000/mm3)					NO - vit D P 0.2
Kelly 2015		YES for RSV vs non-RSV viruses RR 1.85 (however inpatient mortality significantly lower in RSV)<br><br>NO for RSV vs no virus detected.							
Ofman 2020		NO for RSV							
Rajatonirina 2013	NO	YES<br>Influenza A significant on multivariate analysis aOR 0.4.<br><br>NO - viral infection vs bacterial or unknown non-significant<br>Rhinovirus & RSV non-significant							
Tapisiz 2011				NO		YES<br>aOR<br>1.07			
Webb 2012	YES<br>Bacteraemia on admission<br>OR 3.06 at 48 hours<br>OR 4.91 at day 5								
Zhang 2013				NO		NO		NO	NO - glucose, CK, creat, ALT, AST, all non signficant

Table S3l - Other investigated factors for associations with treatment failure

Author & Year	Chest X-Ray Consolidation	Other Chest X-Ray findings	Other investigated factors
Basnet 2015		YES OR 2.22	NO - Zinc Supplementation
Jakhar 2018		YES - Abnormal Chest X- ray 15/15 (100%) failed treatment	YES - lethargy or unconsciousness aOR 114.2, unable to drink<br>NO – overcrowding, smoking family member P 0.39
Moschovis 2013			YES - high altitude and anaemia<br>NO – altitude alone
Nguyen 2019			YES - cooking with biomass fuel OR 1.4, aOR 1.2; Daycare attendance OR 0.4, aOR 0.6, ARI readmission OR 2.0, aOR 1.5, recent TB contact OR 1.9, aOR 2.0.<br><br>NO - cigarette smoke exposure OR 1 aOR 1
Rajatonirina 2013			YES - Clinical diagnosis of bronchiolitis aOR 0.5<br>NO - passive smoking, no of rooms in home
Tapisiz 2011	NO - lobar infiltrates and "bronchopneumonia"	YES - pleural effusion aOR 13.23	
Webb 2012			YES - signs of shock on admission OR 2.52 at 48 hours, OR 2.66 at 5 days. Failure at 48 hours predicted failure at 5 days (at 5 days treatment failure observed in 31% of those who failed treatment at 48 hours and 2.6% of those who responded. OR 17.0
Zhang 2013			YES - Grunting/groaning 89% in failure vs 47% in non-failure, Head nodding 59% in failure vs 11% in non-failure

Table S3m – Associations of hypoxaemia with demographic features

YES = significant association (P<0.05), NO = investigated but no significant association found (P>0.05)

Author & Year	Child's age (months)	Female sex	Breastfeeding	Immunisation Status	Maternal Age	Parental Education	Socio-economic status	Other demographic factors
Alohan 2019	YES<br><12 months OR 2.56	NO						
Benet 2017	YES<br>median age hypoxaemic 12m, median age non-hypoxaemic 15m<br><br>NO - age category 2-11m 19.5% 12-23m 20.8%<br>24-60m 12.1%<br>P 0.13	NO<br>P 0.49		YES<br>DPT-HepB-Hib<br><br>NO<br>PCV<br>P 0.67				
Ferolla 2013	NO	NO	YES<br>OR 0.535<br>aOR 0.447			NO		NO – for house materials tin/mud, dirt floor, sewage, heating, smoking home, crowding
Hasan 2014	YES "received O2"<br>40.7% of 28d - 6m<br>24.4% of 6-23m<br>15.9% of 24-59m							
Khuri-Bulos 2020	YES - "younger age"	YES	YES<br>"lack of breastfeeding"					
Lima 2015	NO<br>P 0.46							
Nemani 2016	NO<br>P 0.543							
Orimadegun 2013	YES - 41.1% of <2 months<br>35% of 2-12m<br>22% of 12-24m<br>23% in 24-59m.	NO						

Table S3n - Associations of hypoxaemia with clinical features

YES = significant association (P<0.05), NO = investigated but no significant association found (P>0.05)

Author & Year	WHO defined severe pneumonia	Hypoxaemia (SaO2 <90% except where specified)	Cyanosis	Tachypnoea (per WHO definition except where specified)	Chest indrawing	Cough	Wheeze	Other auscultation findings	Tachycardia (per WHO definition except where specified)	Pallor	Fever (temperature ≥38°C except where specified)
Benet 2017			YES<br>14.3% of hypoxaemic pts<br>4.8% of non-hypoxaemic<br>P 0.003	YES<br>Rate median 58 in hypoxaemic, 56 in non-hypoxaemic patients P0.009	YES<br>90% of hypoxaemic patients<br>77.2% of non-hypoxaemic patients<br>P 0.02	NO<br>P0.99	NO<br>P 0.08	NO<br>Crackles P 0.7<br>Ronchi<br>P 0.18	YES - mean HR hypoxaemic 151<br>Mean HR non-hypoxaemic 140<br>P 0.001		NO<br>Median temperature P 0.5
Caggiano 2017							YES<br>P <0.05				
Nemani 2016	YES<br>100% of severe pneumonia hypoxaemic<br>19.8% of non-severe		YES<br>59.3% of patients with hypoxia<br>0 patients without<br>P <0.001		YES<br>100% of those with hypoxia<br>64.2% of those without<br>P <0.001	NO<br>P 0.09	NO<br>P0.73				
Nguyen 2019	YES<br>68% of severe required O2, 10.2% of non-severe, and 3.8% of clincally diagnosed pneumonia not meeting WHO criteria										
Orimadegun 2013			YES<br>OR 5.41	YES<br>fast/difficult breathing OR 7.26	NO						
Wandeler 2015			YES<br>OR 19<br>aOR 26	YES for RR >10 above ULN<br>OR 4.5<br>(non-significant on multivariate analysis)	YES for severe chest indrawing OR 14<br>aOR 9.9		NO				NO

Table S3o - Associations of hypoxaemia with comorbidities

YES = significant association (P<0.05), NO = investigated but no significant association found (P>0.05)

Author & Year	Comorbidities (all)	HIV	Asthma	Other chronic respiratory disease	Congenital heart disease	Other heart failure	Neurodevelopmental	Ex-preterm or low birth weight	Anaemia	Diarrhoea	Malaria
Alohan 2019											YES OR 0.55
Benet 2017		NO P 0.57	NO P 0.43	NO P 0.35	NO P 0.46					NO P0.08	
Caggiano 2017	YES P <0.05 (most common malaria, typhoid fever, anaemia										
Ferolla 2013	YES OR 3.34 Not significant on multivariate analysis P 0.15							NO for both			
Khuri-Bulos 2020	YES							YES "lower gestational age"			
Nemani 2016									YES Hb <7g/dl OR 8.5 Hb 7-9.9g/dl OR 3.89		
Onyango 2012					YES 1 of 4 influenza deaths had "chronic heart disease"						

Table S3p - Associations of hypoxaemia with markers of malnutrition

YES = significant association (P<0.05), NO = investigated but no significant association found (P>0.05)

**Author & Year**	**Weight for age Z-score (WAZ) –2 to -3 SD (or where specified <-2)**	**Weight for age Z-score (WAZ) <-3 SD**	**Weight for height Z-score (WHZ) -2 to -3 SD (or where specified <-2)**	**Weight for height Z-score (WHZ) <-3 SD**	**Height for age Z-score -2 to -3 SD (or where specified <-2)**	**Height for age Z score <-3 SD**	**Malnutrition otherwise (or not) specified**
Alohan 2019							NO
Benet 2017	YES for WHZ <-2<br>29.2% hypoxaemic<br>P 0.05			NO<br>P 0.12			
Chisti 2010							NO<br>P 0.85
Ferolla 2013	NO	YES<br><75% expected weight for age<br>OR 3.03<br>aOR 3.46					
Moschovis 2015					YES<br>RR 1.35		
Nemani 2016			YES<br>OR 7.56	YES<br>OR 86.1			
Wandeler 2015							NO

Table S3q - Associations of hypoxaemia with aetiology and laboratory findings

YES = significant association (P<0.05), NO = investigated but no significant association found (P>0.05)

Author & Year	Bacterial	Viral	PjP	Leucocytes	Platelets	CRP	Procalcitonin	Lactate	Other laboratory findings
Benet 2017	YES for pneumococcus serotypes 6AB and 10A from respiratory samples. 6AB 22.9% hypoxic, 9.5% non-hypoxic P = 0.002; 10A 7.1% hypoxic, 1.5% non-hpyoxic P = 0.006<br><br>NO for:<br>S. aureus P 0.99 if in respiratory sample, P 0.13 if in blood sample<br>M. pneumoniae P 0.43<br>Chlamidya spp. P 0.65<br>S. pneumoniae overall P 0.69 if in respiratory sample, P 0.64 if in blood sample.<br>Hib P 0.83 if in respiratory samples, P 0.94 if in blood samples	YES for:<br>Human metapneumovirus OR 2.3, aOR 2.4 (1.0–5.8)<br>RSV OR 2.3 aOR 2.5<br><br>NO for:<br>Coronavirus, enterovirus, parechovirus, rhinovirus, parainfluenza, influenza (including A, B, H1N1), Boca, Adenovirus		YES for:<br>Median 22 in hypoxaemic, and 11.5 in non-hypoxaemic patients P < 0.001<br><br>WCC > 20×10^9 cells/L 55.2% of hypoxaemic, 30.2% of non-hypoxaemic patients P < 0.001<br><br>NO for neutrophils P 0.7		NO P 0.58	YES<br>Median 4.3 ng/ml in hypoxic patients, 1.6ng/ml in non-hypoxic patients<br><br>Procalcitonin > 50 ng/mL 16.3% of hypoxaemic patients, 5.2% of non hypoxaemic patients, P 0.002		
Diez-Padrisa 2010	NO<br>P 0.377								
Ferolla 2013		YES for RSV<br>OR 1.394<br>aOR 2.073							
Fischer Langley 2013		NO for RSV P 0.13							
Graham 2011			YES – “received O2”<br>73% vs 25% of bacterial pneumonia<br>P0.001						
Hasan 2014	YES? – “received O2”<br>42.1% of bacterial vs 29.9% of viral received O2<br>No P value provided								
Khuri-Bulos 2020		YES							YES for low vitamin D level

Nemani 2016						NO - P 0.07			NO for serum albumin
Onyango 2012		YES for influenza OR 1.78							
Ramakrishna 2012								YES SaO2 <80 mean lactate 3.6, vs 2.9 for SaO2 80-90, 2.4 for SaO2 >90	
Rose 2010		NO							
Suntarattiwong 2011		YES for influenza 46.2% hypoxaemic vs 69.2% no virus deteted<br>YES for RSV 77.9% hypoxaemic vs 69.2% with no virus detected P0.006							

Table S3r - Other investigated factors for associations with hypoxaemia

Author & Year	Chest X-Ray Consolidation	Other Chest X-Ray findings	Other investigated factors
Benet 2017	YES for generalized, dense, homogenous opacification 43.5% of hypoxaemic patients, 27.5% of non-hypoxaemic patients P 0.009	YES - pleural effuision 7.3% of hypoxaemic patients, 23.4% of non-hypoxaemic patients P 0.003	YES<br>Conjunctivitis 5.8% of hypoxaemic, 1.2% of non-hypoxaemic patients, P 0.01.<br><br>NO - Previous TB P 0.52, Contact with TB P 0.63, Prostration or lethargy P 0.13, inability to drink P 0.4, convulsions P 0.86
Ferolla 2013			YES for frequent maternal ingestion of carbohydrates during pregnancy - strong association with SaO2 87 or less in children with ARI<br><br>55.6% of infants with pneumonia born to mothers with the highest pregnancy sugar-intake scores presented with SaO2 87% or lower compared with 12.7% of all infants with ARI.<br><br>Dose-dependent significant association found between maternal carbohydrate ingestion in pregnancy and SaO2 87 or less in ARI patients.<br><br>Frequent maternal ingestion of fruits and vegetables during pregnancy had negative association with SaO2 87 or less.<br><br>NO significant association found with maternal protein or fat intake in pregnancy, maternal asthma, IUGR, smoking during pregnancy
Lima 2015		YES<br>Pleural effusion P <0.01	
Moschovis 2013			YES<br>High altitude (>2000m above sea level) - median SaO2 74 vs 89 in low altitude sites. At high altitude, 86% of patients had an SpO2 <80%, compared with 11% of patients at low altitude, RR: 7.7, P < .0001
Nemani 2016		YES<br>Pleural effusion 37% hypoxaemic vs 21% non-hypoxameic. P 0.041	NO for nasal flaring P 0.135.
Orimadegun 2013			YES<br>Grunting OR 5.10, head nodding (only 4 patients, all hypoxaemic)<br><br>NO<br>Nasal flaring, inability to feed, lethargy
Wandeler 2015			YES<br>"poor general status" OR 20, Inability to cry OR 5.9 (non-significant on multivariate analysis), nasal flaring OR 10, grunting OR 9.4<br><br>NO<br>Inability to drink, head nodding

Table S3s – Associations of ICU admission with demographic features

YES = significant association (P<0.05), NO = investigated but no significant association found (P>0.05)

Author & Year	Child's age (months)	Female sex	Breastfeeding status	Immunisation Status	Maternal Age	Parental Education	Socio-economic status (SES)	Other demographic factors
Cotes 2015							YES – lower SES based on state of social security affiliation<br><br>OR 2.5	
Do 2011	NO	NO						YES for exposed to cooking smoke 87% of ICU patients vs 20% of ward patients P0.001
Hasan 2014	YES<br><br>Intubation rates:<br>4.6% of 28d - 6m<br>0.7% of 6-23m<br>0.6% of 24-59m.							
Mildemberger 2017						YES for low father's education level		
Nathan 2014	NO							YES - attends nursery OR 0.37 (for "death or ICU admission")
Ofman 2020	YES for age <6m<br>OR 4.51		YES for "never breastfed"<br><br>OR 2.49	NO	NO	YES for no/incomplete high school education<br><br>OR 2.86		NO - for crowding at home, tobacco smoking at home, smoking in pregnancy
Pedraza-Bernal 2016	NO	YES<br>24.5% of males and 16.7% of females						

		P 0.048<br><br>(not significant on multivariate analysis)						
Shan 2019	YES<br><br>29d to 6m OR 4.88, aOR 4.19<br><br>6m to <12m OR 2.14, aOR 1.93<br><br>NO: 12m to 24m no significant difference<br><br>(all ref 24m to <60m)	YES<br>2.6% of males, 2.1% of females admitted to ICU<br><br>(male sex)<br>OR 1.21<br>aOR 1.18 (aOR not significant)						

Table S3t - Associations of ICU admission with clinical features

YES = significant association (P<0.05), NO = investigated but no significant association found (P>0.05)

Author & Year	WHO defined severe pneumonia	Hypoxaemia (SaO2 <90% except where specified)	Cyanosis	Tachypnoea (per WHO definition except where specified)	Chest indrawing	Cough	Wheeze	Other auscultation findings	Tachycardia (per WHO definition except where specified)	Pallor	Fever (temperature ≥38°C except where specified)
Do 2011		NO for oxygen requirement	NO	YES<br><br>71% of ICU admissions vs 46% of ward patients<br><br>P 0.01	NO	NO	YES<br><br>82% of ICU patients 16% of ward patients<br><br>P 0.001	YES for crepitations<br><br>70% of ICU patients<br>18% of ward patients<br>P 0.001<br><br>NO for stridor			YES for temp > 38.5<br><br>32% of ICU patients<br>69% of ward patients<br><br>P 0.001
Nathan 2014		YES for "signs of hypoxia"<br><br>56.5% of "life threatening (ICU admission or death)<br>5.2% of non life-threatening<br><br>OR 23.8 aOR 17.05<br><br>(hypoxaemia defined as SaO2 <92, not clear what "signs of hypoxia" means)									YES<br>60.1% of "life threatening (ICU admission or death)<br>82.6% of non life-threatening<br><br>OR 0.35
Nguyen 2019	YES for ICU admission<br><br>54% of severe<br>6.6% of non-severe, 3.2% of "clincally diagnosed but not meeting WHO pneumonia criteria"<br><br>YES for assisted ventilation										

	16.7% of severe 1.1% of non severe<br>0.86% of “clincally diagnosed but not meeting WHO pneumonia criteria”										
Shan 2019					YES for respiratory distress<br><br>OR 12.33 aOR 12.1	YES<br><br>OR 0.26, aOR 0.28	YES<br><br>OR 1.1 (non-sig) aOR 1.25 (P 0.017)				NO

Table S3u - Associations of ICU admission with comorbidities

YES = significant association (P<0.05), NO = investigated but no significant association found (P>0.05)

Author & Year	Comorbidities (all)	HIV	Asthma	Other chronic respiratory disease	Congenital heart disease	Other heart failure	Neurodevelopmental	Ex-preterm or low birth weight	Anaemia	Diarrhoea	Malaria
Do 2011								NO for low birth weight			
Lozano-Espinosa 2019					YES for congenital heart disease with hemodynamic repercussion or pulmonary hypertension<br><br>RR 2, 269						
Meligy 2016	NO<br>P 0.46										
Nathan 2014				YES<br>Chronic lung disease 17.4% of “life threatening” (death or ICU admission)<br>2.0% non life-threatening<br><br>OR 10.17<br>aOR 5.89	YES – 14.3% of “life threatening” (death or ICU admission)<br>4.3% of non life threatening<br><br>OR 4.63		YES for "genetic/metabolic disease”<br><br>17.4% of “life threatening” (death or ICU admission)<br>3.5% of non life-threatening<br><br>OR 5.85	NO for preterm<br><br>NO for low birth weight	NO		
Ofman 2020				NO for “recurrent wheeze"<br><br>NO for “ventilated at birth”	YES<br>OR 2.82			NO for preterm<br>“degree of prematurity not significant” (all patients in study were ex-preterm)	YES for haematocrit less than population mean of 30.6%<br>OR 1.99		
Pedraza-Bernal 2016				YES for recurrent wheezing<br>aOR 1.76<br><br>NO for broncho-pulmonary dysplasia	YES<br>43% patients admitted to ICU<br>P 0.01<br>(not significant on multiviariate analysis)			NO for preterm			
Shan 2019			NO		YES<br>OR 4.33<br>aOR 3.03			YES for preterm<br><br>OR 2.41, aOR 1.99			
Zhang 2011	NO										

Table S3v - Associations of ICU admission with markers of malnutrition

YES = significant association (P<0.05), NO = investigated but no significant association found (P>0.05)

Author & Year	Weight for age Z-score (WAZ) –2 to -3 SD (or where specified <-2)	Weight for age Z-score (WAZ) <-3 SD	Weight for height Z-score (WHZ) -2 to -3 SD (or where specified <-2)	Weight for height Z-score (WHZ) <-3 SD	Height for age Z-score -2 to -3 SD (or where specified <-2)	Height for age Z score <-3 SD	Malnutrition otherwise (or not) specified
Nathan 2014	YES for weight <3rd centile (not specified if for age or height) 19.6% of "life threatening" (death or ICU admission)<br>8.4% of non-life-threatening<br><br>OR 2.72						
Ofman 2020	YES for "underweight"<br>OR 3.7						
Pedraza-Bernal 2016							YES<br><br>37.5% of ICU patients<br>P 0.002<br><br>(not significant on multivariate)

Table S3w - Associations of ICU admission with aetiology and laboratory findings

YES = significant association (P<0.05), NO = investigated but no significant association found (P>0.05)

Author & Year	Bacterial	Viral	PjP	Leucocytes	Platelets	CRP	Procalcitonin	Lactate	Other laboratory findings
Ali 2013		YES for H1N1 influenza<br>RR 5							
Awad 2020		NO for RSV							
Do 2011		NO for viral aetiology overall, or for bocavirus, HMPV, enterovirus, rhinovirus, RSV, influenza		NO					
Evelyn 2019		NO (for mechanical ventilation) for HMPV<br>P 0.88							
Fischer Langley 2013		YES for RSV<br>P 0.001<br>(Not significant after controlling for age (P = 0.06).<br><br>RSV NOT significantly associated with mechanical ventilation P0.54							
Hasan 2014	YES?<br>6.9% of patients with bacterial aetiology intubated<br>0.6% of patients with viral aetiology intubated								
Hatem 2019		NO for RSV, adenovirus, influenza							
Korkmaz 2018									Pro-adrenomedullin median value 8.46 vs. 1.8 nmol/L<br>P<0.001<br><br>IL-1β median value 19.69 vs. 8.25 pg/mL<br>P<0.001
Lozano-Espinosa 2019	YES for "bacterial pneumonia"	YES for RSV<br>RR 2.5							

	RR 3.0								
Meligy 2016		NO for rhinovirus, parainfluenza, bocavirus, coronavirus, H1N1 Influenza, enterovirus, HMPV RSV, Adenovirus							
Morrow 2014			NO - for PICU admission; ventilation requirements and duration; or length of PICU stay						
Nathan 2014	NO	NO for RSV				YES - mean CRP 2.8mg/l in “life threatening” (death or ICU admission) 1.4mg/l in non life-threatening<br><br>P 0.04			YES - neutrophils mean 7.8 in “life threatening” (death or ICU admission), vs 5.9 in non-life threatening<br>P 0.04<br><br>Bicarbonate 21 in life threatening, 20 in non life-threatening<br>P 0.02<br><br>NO - hyponatraemia
Ofman 2020		NO for HMPV & RSV							
Pedraza-Bernal 2016		NO for viral aetiology overall, RSV, and Adenovirus							
Saleh 2018									YES for serum zinc level on admission mean 43.5 ug/dl in ventilated vs 63.6 in non-ventilated P 0.001
Shan 2019				YES - "abnormal WCC count” OR 1.39<br>aOR 1.27		YES for CRP >8mg/l<br><br>OR 1.44<br>aOR 2.2			
Suntarattiwong 2011		NO for RSV and influenza							
Zhang 2011	NO								

Table S3x - Other investigated factors for associations with ICU admission

Author & Year	Chest X-Ray Consolidation	Other Chest X-Ray findings	Other investigated factors
Do 2011			YES - previous hospitalisation with respiratory disease 36% ICU 53% ward P 0.008, other family members sick at home 26% ICU 13% ward P0.01,
Nathan 2014			YES - previous pneumonia OR 2.97; previous admission OR 2.05; History of shortness of breath, OR 5.0, History of apnoea OR 9.35 aOR 5.5, signs of lethargy OR 3.91
Ofman 2020			YES - apnoea OR 6.61, sepsis OR 92.8, pneumothorax OR 35.4, clinical pneumonia diagnosis OR 2.18<br>NO - bronchiolitis diagnosis, perinatal infection
Pedraza-Bernal 2016			YES - Pulmonary hypertension aOR 3.62
Shan 2019		NO	
Zidan 2014			YES IL6-174 genotype CC 69.2% admitted to ICU, vs 23.6% in GC genotype and 15.6% in GG genotype

Table S4 – Additional characteristics of studies

Author & Year	Risk factor identification was major aim of study?	Study type	Data collection period	Setting	Country (City/ Region)	WHO Region	World Bank Category	Participant number	Number of paediatric pneumonia patients	Median Age, months	Male %	Immunisation Status (% up to date per schedule or otherwise where specified)	Treatment Failure %	WHO Severe Pneumonia (per 2014 definition) %	Hypoxaemia %	PICU %	Number of deaths	Case fatality rate %
Abdulkadir 2015	NO	Cross-sectional study		Urban tertiary hospital	Nigeria (Ilorin)	AFRO	LMIC	200	200	14.3	59.5				41		17	8.5
Acuna 2018	YES	Retrospective observational	2004-2016	Urban tertiary hospital	Paraguay (Asuncion)	PAHO	UMIC	222	222	38	63					40 MV	42	18.9
Adewuyi 2012	NO	Observational		Urban tertiary hospital	South Africa (Pretoria)	AFRO	UMIC	107	107								18	16.8
Agweyu 2015	NO	Randomised control trial	09/2011 - 08/2013	6 hospitals with paediatric inpatient units including 4 district hospitals and 2 provincial general hospitals	Kenya (3 sites central Kenya, 3 sites Western Kenya)	AFRO	LMIC	527	527	13	57		11.2	6.3			4	0.8
Agweyu 2018	NO	Retrospective cohort	09/2011 - 08/2013	6 hospitals with paediatric inpatient units including 4 district hospitals and 2 provincial general hospitals	Kenya (3 sites central Kenya, 3 sites Western Kenya)	AFRO	LMIC	1709	16162	12	56			22			832	5.2
Agweyu 2018	YES	Retrospective cohort	03/2014 - 02/2016	14 district hospitals	Kenya (14 sites)	AFRO	LMIC	16162	1,709	13							18	1.1
Ahmed 2016	NO	Prospective cross-sectional	01/2014 - 12/2014	Urban tertiary hospital	Pakistan (Karachi)	EMRO	LMIC	540	540		61.3						46	8.5
Ahmed 2018	NO	Retrospective cohort	01/2011 - 12/2014	National referral hospital	Mauritania, Nouakchott (capital city)	AFRO	LMIC	665	665		44.5						120	18.1
Al Amad 2019	NO	Retrospective cohort	2011 - 2016	2 urban hospitals	Yemen, Aden city and Sana'a	EMRO	LIC	1413	1413							40	126	9
Ali 2013	NO	Prospective surveillance study	08/2009 - 09/2011	Tertiary private hospital	Pakistan, Karachi	EMRO	LMIC	812	812	9.7							13	1.6
Alohan 2019	NO	Cross sectional study	09/2016 - 09/2017	3 secondary health facilities	Nigeria (Southwest)	AFRO	LMIC	379	379	14	48				35.6		25	6.6
Araya 2016	YES	Retrospective observational	01/2004 - 06/2013	Urban tertiary hospital	Paraguay (Asuncion)	PAHO	UMIC	860	860	34	56					11.9 ICU 8.9 MV	56	6.5
Atwa 2015	YES	Prospective Cross sectional	10/2012- 08/2014	Tertiary hospital	Egypt	EMRO	UMIC	242	242	17	55.8						9	3.7

Awad 2020	NO	Prospective surveillance study	15/1/2016 - 15/4/2016	2 Tertiary referral hospitals	Irbid, North Jordan	EMRO	LMIC	479	479	10.4	63				8.1	10.2	3	0.6
Awasthi 2018	NO	Prospective cohort study with nested case-control	02/2014 - 06/2016	Urban tertiary hospital	India (Lucknow)	SEARO	LMIC	350	350								24	6.9
Ayieko 2012	NO	Cross sectional study	03/2007 - 03/2008	9 rural district hospitals	Kenya	AFRO	LMIC	3372	3372	12	53.2			16.1			195	5.9
Azab 2016	NO	Case control	08/2013-10/2015	university hospital (tertiary urban)	Egypt (Zagazig)	AFRO	LMIC	100	100	41	53					25	8	8
Azab 2014	NO	Prospective longitudinal cohort study	08/2009-6/2013	university hospital (tertiary urban)	Egypt (Zagazig)	AFRO	LMIC	1470	1470	65	60.6	66.50%			16.4		237	16
Barger-Kamate 2016	NO	Case control	08/2011 - 01/2014	9 centres	Kenya, Zambia, South Africa, Mali, The Gambia, Bangladesh, and Thailand	AFRO, SEARO	LIC, LMIC, UMIC	4200	4200 cases and 5196 controls									
Basnet 2015	NO	Prospective cohort study	02/2006-06/2008	1 tertiary referral urban children's hospital	Nepal (Kathmandu)	SEARO	LMIC	610	610	6	61		35	49.2	62	1.1	4	0.7
Bekele 2017	YES	Cross sectional study		Urban tertiary hospital	Ethiopia (Jimma)	AFRO	LIC	107	107		54.2	52.3% fully 39.3% partially 8.41% none					5 died, 4 DAMA, 4 unknown	4.7% died, 3.7% DAMA 3.7% unknown
Benet 2017	YES	Prospective longitudinal	05/2010 - 06/2013	Five hospitals	India, Madagascar, Mali, Paraguay	AFRO, SEARO, PAHO	LIC, LMIC, UMIC	405	405	14	58				17.3		14	3.5
Berkley 2010	NO	Prospective observational and case-control study	01/2007 - 12/2007	Rural district hospital	Kenya (Kilifi)	AFRO	LMIC	759	759	9	59						24	3.2
Bezerra 2011	NO	Prospective Cross sectional	04/2008-03/2009	1 Public teaching hospital	Recife, Pernambuco, NE Brazil	PAHO	LMIC	407	407	8	58			10.3	10.3		3	0.7
Bills 2020	YES	Prospective observational	06/2016 - 09&2016	Public ambulances	India (Andhra Pradesh, Assam, Gujarat, Himachal Pradesh, Karnataka,	SEARO	LMIC	1433	1433	24	54.2						94/1215	7.7

					Meghalaya and Telangana)													
Bjorklund 2019	NO	Prospective, non-blinded, non-randomised interventional study	Historical data from 04/2015-06/2015. Trial enrolment from 07/2015-06/2016.	1 Public government referral hospital		AFRO	LMIC	83	83	15.6	53						8	9.6
Bokade 2015	YES	Observational	2010-2012	Tertiary hospital	India	SEAR O	LMIC	290	290		65.9					23.8	25	8.6
Boukari 2011	NO	Retrospective observational	10/2008 - 10/2010	Urban hospital	Algeria (Blida)	AFRO	LMIC	221	221	11.9	54.3	87% ()% PCV)					7	3.2
Caggiano 2017	NO	Prospective observational study		Rural hospital	Tanzania (Itigi)	AFRO	LMIC	100	100	33	47			24			11	11
Champatiray 2017	NO	Prospective observational	09/2013 - 08/214	Urban tertiary hospital	India (Cuttack)	SEAR O	LMIC	141	141	5	61	97.16%		40.4			31	22
Chisti 2010	NO	retrospective chart review	05/2005 - 04/2006	Urban tertiary hospital	Dhaka, Bangladesh	SEAR O	LMIC	48	48	3							7	14.6
Chowdury 2020	NO	Case control	04/2015-12/2017	1 Urban referral hospital	Dhaka, Bangladesh	SEAR O	LMIC	360	360	8	62	87% BCG 37% PCV					40	11.1
Cohen 2015	NO	Prospective surveillance study	02/2009 - 12/2012	1 urban, 1 periurban and 2 rural hospitals	South Africa (Gauteng Province, KwaZulu-Natal Province, Mpumalanga Province)	AFRO	UMIC	8723	8723			78% Hib, 46% PCV, 0.07% influenz		53	35% receive d O2		150	1.8
Cotes 2015	NO	Retrospective case study	04/2000 - 11/2006	1 urban paediatric hospital and 2 urban general hospitals	Colombia (Bogota, Manizales)	PAHO	UMIC	535	535	8	53.6						19	3.6
Daga 2014	NO	Observational		Tertiary care centre	India (Pune)	SEAR O	LMIC	616	616								140	22.2
Dembele 2019	YES	Prospective observational	06/2008 - 03/2016	Two secondary-care hospitals, one tertiary-care hospital and one urban research centre	Philippines (Biliran, Palawan, Manila, Tacloban)	WPR O	LMIC	4305	4305								198	4.6
Diez-Padrisa 2010	NO	Prospective observational	09/2006 - 09/2007	Rural district hospital	Mozambique (Manhiça District)	AFRO	LIC	176	176		63.6						17	9.7
Divecha 2019	YES	Prospective observational	01/2011 - 06/2012	Tertiary hospital PICU	India, Mumbai	SEAR O	LMIC	293	293	18.7	61.77	66.9% fully				100 PICU,	90	30.7

																63 MV		
Do 2011	NO	Prospective descriptive	11/2004-01/2008	Urban referral hospital	Vietnam (Ho Chi Minh City)	WPRO	LMIC	309	309						12.6	26 PICU, 0.3 MV	2	0.6
Durigon 2015	NO	Prospective surveillance study	03/2008 - 02/2010	Urban tertiary referral hospital	Brazil (São Paolo)	PAHO	UMIC	622	715							14 ICU, 13 MV	9	1.2
Emukule 2014	YES	Observational	08/2009 - 07/2012	Rural district hospital	Kenya (Siaya)	AFRO	LMIC	3581	3581								218	6
Enarson 2015	YES	Prospective cohort	10/2000 - 06/2003	16 district hospitals	Malawi (whole country)	AFRO	LIC	15709	15,709								1633	10.4
Enarson 2014	NO	Prospective interventional, non-randomised stepped wedge design	10/2000 - 12/2005	24 district hospitals including 3 tertiary referral hospitals	Malawi (whole country)	AFRO	LIC	47228	47 228								4605	9.8
Evelyn 2019	NO	Cross-sectional Study	10/2015 - 12/2017	Urban Tertiary hospital	Colombia (Bogota)	PAHO	UMIC	420	420	43	56.4					24 MV	30	7.1
Ezeonu 2015	NO	Retrospective case note review	01/2005 - 01/2010	Rural tertiary hospital	Nigeria (Abakaliki)	AFRO	LMIC	239	239		58.6						18	7.5
Fagbohun 2020	NO	Observational		secondary health centres with limited facilities	Nigeria (Southwest)	AFRO	LMIC	519	519		49.5						43	8.3
Fancourt 2017	NO	Observational	08/2011 - 01/2014	9 centres	Kenya, Zambia, South Africa, Mali, The Gambia, Bangladesh, and Thailand	AFRO, SEARO	LIC, LMIC, UMIC	3587	3587					30.8			373	8.8
Feikin 2017	NO	Observational	08/2011 - 01/2014	9 centres	Kenya, Zambia, South Africa, Mali, The Gambia, Bangladesh, and Thailand	AFRO, SEARO	LIC, LMIC, UMIC	1733	1733 cases and 4986 controls		56.4			29.2				
Ferolla 2013	NO	Prospective observational		Urban hospitals	Argentina (Buenos Aires, La Plata)	PAHO	UMIC	1,293	1,293		54				12.7		22	1.7

Ferreira 2014	YES	longitudinal, hospital-based observational study,	01/1996 - 12/2011	Urban tertiary referral hospital	Brazil (Rio de Janeiro)	PAHO	UMIC	871	860		55						26	3
Fischer Langley 2013	NO	Prospective, active hospital-based surveillance	11/2007 - 07/2010	3 referral hospitals	Guatemala (Guatemala City, Queteltenango, Santa Rosa)	PAHO	UMIC	2193	2193	9.2							70	3.2
Gallagher 2020	YES	Observational	08/2011 - 11/2012	9 centres	Kenya, Zambia, South Africa, Mali, The Gambia, Bangladesh, and Thailand	AFRO, SEARO	LIC, LMIC, UMIC	1802	1802	9	57						120	6.6
Gowraiah 2014	NO	prospective observational	10/2012 - 04/2013	4 urban public hospitals - outpatients and inpatients	India (Lucknow, Bangalore)	SEARO	LMIC	524	524	11	63.9						8	1.6
Graham 2019	YES	Prospective, clinical descriptive		Urban Tertiary referral hospital	Malawi (Blantyre)	AFRO	LIC	327	4133						37.3		149	7.2
Graham 2011	NO	Prospective cohort study nested within a stepped-wedge trial	11/2015 - 10/2017	12 secondary-level hospitals	Nigeria (Southwest)	AFRO	LMIC	2073	327	11	54	>90%					33	10.1
Hasan 2014	NO	surveillance	2005 - 2010	2 provincial, 16 district, 2 military hospitals (10-140 inpatient beds)	Thailand (Sa Kaeo and Nakhon Phanom provinces)	SEARO	UMIC	28543	28,543		59				9% hypoxic SaO2<90%, 22% received O2	1.1 MV	98	0.3
Hatem 2019	NO	surveillance	02/2010 - 02/2014	Urban tertiary referral hospital	Egypt (Cairo)	EMRO	UMIC	961	981							18.1 ICU, 6.7 MV	13	1.3
Hooli 2016	NO	Retrospective observational	10/2011 - 06/2014	7 district hospitals	Malawi (Mchinji and Lilongwe)	AFRO	LIC	14665	14,665								464	3.2
Hutton 2019	YES	retrospective cohort study	01/2012 - 12/2012	Urban tertiary hospital, PICU	South Africa (Cape Town)	AFRO	UMIC	358	265	4	59.3					100 PICU 72.5 MV	34	12.8
Ibraheem 2020	NO	retrospective descriptive cross-sectional study	01/2013 - 12/2017	Urban teaching hospital	Nigeria (Ilorin)	AFRO	LMIC	971	971		51.3						81	8.3

Indriyani 2018	NO	Prospective observational	01-2018 - 10/2018	Urban regional hospital	Indonesia (West Nusa Tenggara Province)	SEARO	UMIC	90	392		57.1			2.3			24	6.1
Indriyani 2019	NO	Retrospective observational	01/2015 - 12/2016	Urban regional hospital	Indonesia (West Nusa Tenggara Province)	SEARO	UMIC	392	90		59							
Iroh Tam 2018	NO	Analysis of data from 2 existing cohort studies	11/2012 - 12/2013	Urban regional referral hospital	Uganda (Mbarara)	AFRO	LIC	382	382									
Jain 2018	YES	Prospective observational	08/2014 - 07/2015	Urban tertiary hospital	India (Lucknow)	SEARO	LMIC	152	152	19.5	60.5	63.2% fully, 11.8% partial					11	7.2
Jakhar 2018	NO	Prospective cohort	10/2012 - 09/2013	Urban tertiary hospital	India (Delhi)	SEARO	LMIC	120	120	11.9	61.7	59% fully, 33% partially, 7.5% none	12.5				0	
Jroundi 2014	NO	Prospective observational	10/2010 - 12/2011	Urban tertiary referral hospital	Morocco (Rabat)	EMRO	LMIC	689	700	21.4	64	87%; 97.8% at least one dose Hib, 16.7% at least one dose PCV					28	4
Jroundi 2014	NO	Surveillance	10/2010 - 12/2011	Urban tertiary referral hospital	Morocco (Rabat)	EMRO	LMIC	700	689	21.4	64	86%; 97.4% at least 1 dose Hib		27.4		8	28	4.1
Julien 2020	NO	Prospective observational	07/2017- 06/2018	Urban referral hospital	Bhutan (Thimpu)	SEARO	LMIC	189	189	10.8	57.7	75.7% fully, 22.7% partially, 1.6% unknown		79.4	75% received O2	15.9	6	3.2
Kelly 2015	NO	Prospective cohort	04/2012 - 10/2013	Urban referral hospital	Botswana (Gaborone)	AFRO	UMIC	238	310	6.1	55		34	34	60	2 MV	18	5.8
Kelly 2015	NO	Prospective cohort and case-control studies	04/2012 - 08/2014	Urban referral hospital	Botswana (Gaborone)	AFRO	UMIC	310	238	6	55		33		61	2 MV	14	5.9
Kelly 2019	NO	Prospective cohort	04/2012 - 06/2016	Urban referral hospital	Botswana (Gaborone)	AFRO	UMIC	390	390	7.4	57	99.4% both Hib and PCV13	32	33	36% hypoxic, 61% received O2	3 MV	19	5.4
Khuri-Bulos 2020	NO	surveillance	03/2010 - 03/2013		Jordan	EMRO	UMIC	3168	3168	3.5	60				32% received O2	9 ICU, 4 MV	31	1
Kim 2019	NO	Prospective cohort		Provincial hospital	Vietnam (Central)	WPRO	LMIC	3817	3817 with clinically diagnosed, 2199 WHO criteria.								189 death or ICU admission	8.6 of those meeting WHO criteria

King 2015	NO	Prospective cohort study (nested within a larger parent study assessing the impact of PCV introduction in Malawi.)	09/2013 - 06/2014	Rural primary care & community health workers	Malawi (Mchinji and Lilongwe districts)	AFRO	LIC	769	769	21.7			14.8					
Korkmaz 2018	NO	Prospective observational	01/2014 - 02/2015	Tertiary hospital	Turkey (Samsun)	EURO	UMIC	66	66	42	59.1					19.7 ICU, 9.1 MV	1	1.5
Ku 2020	NO	Retrospective Observational	01/2010 - 07/2014	Rural emergency department	Uganda (Rukungiri district)	AFRO	LIC	1238	1238									
Kuti 2013	YES	Observational	11/2010 - 04/2011	Rural referral hospital	Gambia (Basse)	AFRO	LIC	420	420	18	55.2	81.9%, including at least one dose PCV-7					15	3.6
Laman 2013	YES	Observational		Urban Tertiary referral hospital	Papua New Guinea (Port Moresby)	WPR O	LMIC	77	77						26% <90, 13% <85		4	5.2
Lanaspa 2015	NO	Observational / surveillance	09/2006 - 09/2007	District hospital	Mozambique (Manhiça)	AFRO	LIC	926	834	10.5							102	12.2
Lazzerini 2016	YES	Retrospective Observational	2001-2012	22 of 23 district hospitals, three of four central hospitals, and 16 of 37 Christian Hospital Association of Malawi facilities	Malawi (whole country)	AFRO	LIC	113154	113 154								6903	6.6 in 2001 4·5 in 2012
le Roux 2015	NO	Prospective cohort	05/2012-05/2014	Community	South Africa (Cape Town)	AFRO	UMIC	109	141								2	1.4
Lima 2015	NO	Prospective and descriptive study	10/2010 - 09/2013	teaching hospital	Brazil (Recife)	PAHO	UMIC	452	452		51.8	50.4% PCV10			48.4	3.8	7	1.5
Lozano-Espinosa 2019	NO	Cohort-type analytical study	08/2017 - 06/2018	Paediatric referral hospital	Colombia (Bogota)	PAHO	UMIC	217	217					2.4			?	
Lufesi 2015	YES	Prospective cohort	2001 - 2012	40 facilities	Malawi, multiple sites	AFRO	LIC	105413	105413								6903	6.6
Ma 2019	NO	Prospective cohort study	09/2013 - 07/2015	1 Regional Referral Hospital and 1 District Hospital	Uganda (Jinja, Kambuga)	AFRO	LIC	155	155	11	42						22	14.2
Macphers on 2019	YES	Retrospective cohort	3/2014 - 02/2018	13 purposely selected public county hospitals, situated in regions of	Kenya, 13 sites	AFRO	LMIC	1832	1832	84	55.9			32.7	22.17		145	7.9

				high and low malaria transmission, which are representative of district-level health facilities in Kenya.														
Matthew 2015	NO	surveillance	04/2011 - 03/2013	Urban tertiary hospital	India (Chandigarh)	SEAR O	LMIC	2345	2345		71.7			13.3			108	4.6
McCollum 2019	NO	open-label, randomised, superiority trial	06/2015 - 03-2018	Rural district hospital	Malawi (Salima)	AFRO	LIC	644	644	7.7	53.6						88	13.7
McCollum 2020	NO	Case control	12/2012 - 01/2014	Hospitals	Bangladesh, The Gambia, Kenya, South Africa, Thailand, and Zambia	AFRO, SEAR O	LICs, LMICs, UMICS	618	618					34.8			69	11.2
Meligy 2016	NO	Prospective descriptive	10/2013 - 03/2014	Urban university hospital	Egypt (Cairo)	EMR O	UMIC	44	44	9	54.5				100	72.7 ICU, 50 MV	11	25
Mildember ger 2017	NO	Observational	2010 - 2015	Urban university hospital	Brazil (Curitiba)	PAHO	UMIC	184	184	Approx. 2 years						22.2		
Mohamed 2017	NO	Prospective case control	01/2016 - 12/2016	Urban tertiary hospital	Egypt (Minia)	EMR O	LMIC	40	40	12.6	57.5							
Morrow 2014	NO	Prospective observational	11/2006 - 08/2008	Urban tertiary hospital	South Africa (Cape Town)	AFRO	UMIC	202	202		45.5				100		51	25
Moschovis 2015	NO	Secondary analysis of clinical trial data	08/2000 - 04/2004	8 urban tertiary hospitals	Dhaka, Bangladesh (4 m); Guayaquil, Ecuador (4 m); Multan, Pakistan (122 m); Chandigarh, India (350 m); Rawalpindi, Pakistan (500 m); Lusaka, Zambia (1300 m); Sana'a, Yemen (2250 m); and Mexico City, Mexico (2420 m)	AFRO, PAHO , EMR O, SEAR O	LIC, LMICs, UMICs	958	2542	7.5	61.8	81.50%						

Moschovis 2013	NO	Secondary analysis of clinical trial data (APPIS and SPEAR trials)	05/1999 - 04/2004	16 tertiary hospitals	Bogota, Columbia; Cape Town, South Africa; Durban, South Africa; Ho Chi Minh City, Vietnam; Islamabad, Pakistan; Kumasi, Ghana; Mexico City, Mexico; Nagpur, India; Ndola, Zambia; Chandigarh, India; Dhaka, Bangladesh; Guayaquil, Ecuador; Lusaka, Zambia; Mexico City, Mexico; Multan, Pakistan; Rawalpindi, Pakistan; Sana'a, Yemen	AFRO, PAHO, EMRO, SEARO	LIC, LMICs, UMICs	2542	958	5.4	61.4	67.30%			26			
Myers 2019	NO	Observational	02/2014 - 04/2014	Urban tertiary hospital	Malawi (Lilongwe)	AFRO	LIC	62	62					62			8	12.9
Naheed 2019	NO	Surveillance	05/2004 - 12/2008	7 Tertiary teaching hospitals, 6 urban, 1 rural. 3 government, 3 private	Bangladesh (Dhaka, Chittagong, Tangail)	SEARO	LMIC	6856	6856	10.1	65			19.9	65.6	4.7	276	4
Nantanda 2014	NO	Prospective observational	08/2011 - 06/2012	Urban Tertiary referral hospital	Uganda (Kampala)	AFRO	LIC	614	614		56.5						22	3.6
Nathan 2014	YES	retrospective observational	11/2010 - 11/2011	Urban tertiary referral hospital	Malaysia (Kuala Lumpur)	WPRO	UMIC	391	391	8		85% Hib, 0% PCV, 1.8% influenza	11.8		59.3	4.3 MV	5	1.3
Negash 2019	NO	Prospective observational study	09/2016 - 08-2017	2 large urban hospitals	Ethiopia (Addis Ababa)	AFRO	LIC	549	549	9	58.5						13	2.37
Nemani 2016	NO	Prospective observational	07/2013 - 06/2014	Urban tertiary teaching hospital	India (Lucknow)	SEARO	LMIC	135	135	18.3				25.2	40			

Nguyen 2019	NO	Prospective descriptive	07/2017 - 06/2018	Urban secondary referral hospital	Vietnam (Da Nang)	WPRO	LMIC	4206	4206				18.7		11.1	8 PICU, 2 MV	16	0.4
Nimdet 2017	NO	retrospective cohort	06/2011 - 06/2014	Urban Provincial hospital	Thaialnd (Surat Thani)	SEARO	UMIC	135	135							100	3	2.2
O'Callaghan-Gordo 2011	NO	Surveillance	09/2006-09/2007	Rural district hospital	Mozambique (Manhiça District, Maputo Province)	AFRO	LIC	835	835	11							33	9 (of children with virus detected and known outcome)
Ofman 2020	YES	prospective, population-based, cross-sectional study	2011-2013	Urban hospitals	Argentina (Buenos Aires, La Plata)	PAHO	UMIC	664	664							9	15	2.26
Olsen 2010	NO	Surveillance	09/2003 - 08/2005	Two provincial hospital, sixteen district hospitals, two military hospitals in 2 provinces (mix of urban and rural)	Thailand (Sa Kaeo and Nakhon Provinces)	SEARO	UMIC	1325	1325								3	0.23
Onyango 2012	NO	Surveillance	01/2007 - 12/2010	Rural District Hospital and health clinics	Kenya (Kilifi)	AFRO	LMIC	2429	2429	9	50			20.7			158	6.5
Orimadegun 2013	NO	Cross-sectional Study	04/2010 - 03/2011	Urban tertiary hospital	Nigeria (Ibadan)	AFRO	LMIC	333	313		60				49.2		33	10.5
Pagano 2018	NO	Observational		Urban Hospitals x2	Uganda (Mbarara)	AFRO	LIC	185	185								6	3.24
Pale 2017	NO	Surveillance	01/2015 - 01/2016	Urban Tertiary hospital	Mozambique (Maputo)	AFRO	LIC	450	450	6	51.5				4.2		2	0.44
Pedraza-Bernal 2016	YES	Prospective cohort study	01/2014 - 01/2015	Urban Tertiary university hospital	Colombia (Bogota)	PAHO	UMIC	416	416	6	51					20.7, 16.1 MV	0	0
Pulsan 2019	NO	Prospective observational study	03/2014 - 08/2016	Urban tertiary referral hospital	Papua New Guinea (Port Moresby)	WPRO	LMIC	64	48	3				75			27	56.3
Rajatonirina 2013	YES	Prospective cohort study	11/2010 - 07/2012	Urban tertiary referral hospital	Madagascar	AFRO	LIC	290	290	13	56.2	87% BCG, 77% Hib, 0% PCV, 0.7% influenza					9	3
Ramachandran 2012	YES	Retrospective chart review	01/2006 - 12/2008	Urban tertiary referral hospital	India (Chennai)	SEARO	LMIC	4375	4375		58						357	8.2
Ramakrishna 2012	NO	Analysis of data from a prospective cohort study	07/2005 - 11/2006	Urban referral hospital	Malawi (Blantyre)	AFRO	LIC	233	233	11				26			25	10.7

Reed 2012	YES	Secondary analysis of clinical trial data	1998-2001	Urban tertiary hospital	South Africa (Soweto)	AFRO	UMIC	4148	4148								298	7.18
Rose 2010	NO	Prospective cohort study		Urban paediatric hospital	Brazil (Recife)	PAHO	UMIC	457	457									
Saghafian-Hedengren 2017	NO	Nested cohort study in broader surveillance study	04/2011 - 02/2013	Community and hospital	India (Chandigarh)	SEARO	LMIC	196	196					49.5			9	4.6
Saha 2016	YES	Surveillance	01/2011 - 12/2013	2 x Urban children´s referral hospitals	Bangladesh (Dhaka)	SEARO	LMIC	3639	3639					61			63	2
Saleh 2018	NO	Prospective cohort study	01/2016 - 10/2017	Urban university hospital	Egypt (Menoufia)	(1) age beyond 2 months up to 59 months; (2) parental consent; (3) all the cases clinically diagnosed with pneumonia and classified according to the revised WHO guidelines	LMIC	480	480		44.2			66.7		66.7 MV	128	26.7

Shan 2019	YES	Retrospective observational	01/2010 - 12/2014	Urban tertiary Children's hospital	China (Suzhou)	WPRO	UMIC	28043	28043		62.4					2.4	359 (includes died and not cured at discharge)	1.28
Solis-Chaves 2018	NO	Case control	01/2010 - 01/2015	Urban tertiary Children's hospital	Costa Rica (San Jose)	PAHO	UMIC	160	160	18.2 (mean)	56.9			50			3	1.9
Srinivasan 2012	NO	Randomized double blind placebo-controlled clinical trial	09/2006 - 03/2007	Urban national teaching and referral hospital, paediatric ward	Uganda (Kampala)	AFRO	LIC	352	352	17.9 (mean)	56.2						28	8
Sudarwati 2014	NO	Surveillance	2007-2009	Hospital	Indonesia	SEARO	UMIC	160	160								6	3.75
Suntarattiwong 2011	NO	Prospective observational	12/2007 - 08/2009	Paediatric hospital Thailand (Bangkok)	Thailand (Bangkok)	SEARO	UMIC	354	354	7 (mean)	64				70		3	0.85
Sutcliffe 2016	NO	Prospective interventional	10/2011 - 03/2014	Urban university teaching hospital	Zambia (Lusaka)	AFRO	LMIC	693	693		53	75.6% at least one dose Hib, 5.3% at least one dose PCV					126	18.2
Suzuki 2012	NO	Prospective observational	05/2008 - 05/2009	Tertiary government hospital	Philippines (Tacloban City)	WPRO	LMIC	819	819	9	54.5			44.4			88 (including 18 likely died at home)	10.7 (of which 2.2 presumed died at home)
Tapisiz 2011	NO	Retrospective study	2000 - 2008	Urban university teaching hospital	Turkey, Ankara	EURO	UMIC	501	501	37	55.3		6.2			2.9	1	0.2
Tomczyk 2019	YES	Retrospective review	09/2007 - 12/2013	3 hospitals, 2 urban, 1 rural	Guatemala (Guatemala City, Queteltenango, Santa Rosa)	PAHO	UMIC	4109	4109								174	4
Tuti 2017	YES	Retrospective cohort	02/2014 - 02/2016	14 Public Hospitals	Kenya	AFRO	LMIC	10687	10687		54.8						252	2.36
Walk 2016	NO	Prospective observational	07/2012 - 09/2012	Urban referral hospital	Malawi (Kamazu)	AFRO	LIC	77	77	5	43		47				36	47
Wandeler 2015	NO	Prospective observational	06/2002 - 01/2003	Rural hospital	Senegal (Ndioum)	AFRO	LMIC	70	70	17.4	53				43		7	10
Webb 2012	NO	Prospective cohort study	05/2007 - 05/2008	Rural district hospital	Kenya (Kilifi)	AFRO	LMIC	568	568	11	57			29			34	6

Wilson 2017	NO	Open-label, cluster, crossover trial	01/2014 - 12/2015	2 non-tertiary hospitals (one district hospital, one municipal hospital)	Ghana (Mampong, Kintampo)	AFRO	LMIC	2200	2200								70	3.21
Zabihullah 2017	YES	Prospective observational	12/2012 - 03/2013	700 bed regional referral hospital	Afghanistan (Mazar-e Sharif)	EMR O	LIC	639	639	5	64.3						75	12.1
Zampoli 2011	NO	Prospective observational	12/2006 - 06/2008	Urban Tertiary referral hospital	South Africa (Cape Town)	AFRO	UMIC	202	202	3.1	45					54	51	25
Zeeshan 2020	YES	Retrospective cohort	01/2013 - 03/2018	PICU in university hospital	Pakistan (Karachi)	EMR O	LMIC	187	187		65.2	52% complete vaccinated, 34.8% partial vaccination						
Zhang 2013	NO	Prospective cohort study	09/2013 - 07/2015	One district and one regional hospital	Uganda (Jinja, Kambuga)	AFRO	LIC	65	707	4	63	No patients had received Hib or PCV vaccines	21		100		41	5.8
Zhang 2011	NO	Prospective, observational	10/2004 - 10/2005	University Hospital, Paediatric department	China (Lanzhou)	WPR O	UMIC	853	853	28	56					12 PICU, 2.6 MV	5	0.6
Zhang 2020	YES	Prospective observational	01/2007 - 12/2010	10 Bed PICU in urban University Children's Hospital	China (Suzhou)	WPR O	UMIC	10836	65	19	57				100		4	6.2
Zhu 2012	NO	Prospective descriptive	01/2009 - 12/2009	23 PICUs	China, 23 urban PICUS	WPR O	UMIC	276	276								72	26.1
Zidan 2014	NO	Prospective cohort	05/2011 - 06/2013	Urban University Hospital	Egypt (Zagazig)	EMR O	LMIC	100	100	25	52		35			27	7	7
Zurita-Cruz 2020	NO	Cross-sectional Study	01/2013 - 12/2017	All IMSS health facilities in the country, including 1st, 2nd and 3rd level facilities	Mexico (whole country)	PAHO	UMIC	66304	66304	14.7 weeks (mean)	61					0.5	371	0.56
